# Molecular evolution of genes encoding allergen proteins in the peanuts genus *Arachis*: Structural and functional implications

**DOI:** 10.1371/journal.pone.0222440

**Published:** 2019-11-01

**Authors:** Khidir W. Hilu, Sheena A. Friend, Viruthika Vallanadu, Anne M. Brown, Louis R. Hollingsworth, David R. Bevan

**Affiliations:** 1 Department of Biological Sciences, Virginia Tech, Blacksburg, VA, United States of America; 2 Research and Informatics, Virginia Tech, Blacksburg, VA, United States of America; 3 Department of Biochemistry, Virginia Tech, Blacksburg, VA, United States of America; Midwestern University, UNITED STATES

## Abstract

Food allergies are severe immune responses to plant and animal products mediated by immunoglobulin E (IgE). Peanuts (*Arachis hypogaea* L.) are among the top 15 crops that feed the world. However, peanuts is among the “big eight food allergens”, and allergies induced by peanuts are a significant public health problem and a life-threatening concern. Targeted mutation studies in peanuts demonstrate that single residue alterations in these allergen proteins could result in substantial reduction in allergenicity. Knowledge of peanut allergen proteins is confined to the allotetraploid crop and its two progenitors. We explored frequencies and positions of natural mutations in the hyperallergenic homologues *Ara h 2* and *Ara h 6* in newly generated sequences for 24 *Arachis* wild species and the crop species, assessed potential mutational impact on allergenicity using immunoblots and structural modeling, and evaluated whether these mutations follow evolutionary trends. We uncovered a wealth of natural mutations, both substitutions and gaps, including the elimination of immunodominant epitopes in some species. These molecular alterations appear to be associated with substantial reductions in allergenicity. The study demonstrated that *Ara h 2* and *Ara h 6* follow contrasting modes of natural selection and opposing mutational patterns, particularly in epitope regions. Phylogenetic analysis revealed a progressive trend towards immunodominant epitope evolution in *Ara h 2*. The findings provide valuable insight into the interactions among mutations, protein structure and immune system response, thus presenting a valuable platform for future manipulation of allergens to minimize, treat or eliminate allergenicity. The study strongly encourages exploration of genepools of economically important plants in allergenicity research.

## Introduction

Peanuts (*Arachis hypogaea* L.) are an important global commodity, being among the top 15 crops that feed the world and second only to soybean in terms of legume crop global production (FAOSTAT, www.fao.org/faostat). However, peanuts are one of the most common causes of severe food allergies being one of the “big eight food allergens, with worldwide prevalence of 0.5–2%, and are on the rise [1–3]. Allergies induced by peanuts are a significant public health problem and a life-threatening concern. The number of reported cases of children with food allergies rose by an alarming 18% from 1997 to 2007 [4], with allergies persisting beyond childhood [5], further accentuating the allergenicity problem. The symptoms of peanut allergy range from mild oral allergy syndrome to anaphylactic reactions to death [6, 7]. At present, there is no cure for peanut allergies. Immunotherapy has been used for gradual increase in tolerance to peanut allergenicity [8], but elevated sensitivity to treatment dosages at extremely small quantities could be hazardous. This has resulted in an agro-economic setback since peanuts seeds are valuable source of protein and oils, and used commercially in various industries [9, 10].

Food allergies are severe immune responses mediated by immunoglobulin E (IgE) to specific foods [1]. Over 50% of plant allergens, including peanuts allergens, belong to four structural protein families: prolamin superfamily, cupin superfamily, profilins, and Bet v-1-related proteins [7, 11–13]. The peanut species *Arachis hypogaea* is reported to contain 17 allergens (Ara h 1–17), with Ara h 4 being recently renamed Ara h 3.02, a total number of 16 allergens in peanut seeds are recognized (Allergen Nomenclature Sub-Committee of the World Health Organization/International Union of Immunological Societies, WHO/IUIS). *Ara h 2* and *Ara h 6* (prolamin superfamily, 2S albumin family) and Ara h 1 and Ara h 3 (cupin superfamily) are regarded as the major allergens in peanuts [14, 15], being recognized by >50% of allergenic people. *Ara h 2* is considered to be the most potent allergen protein [14, 16, 17] since it is recognized by serum IgE of over 90% of the peanut-sensitive patients [16]. *Ara h 6* shares similar immunological features with *Ara h 2* [9, 18, 19], rendering these two proteins as the most powerful of all peanut allergens. Zhuang and Dreskin (2013) [20] noted that removing *Ara h 2* and *Ara h 6* allergen proteins together significantly reduced the effector activity of the peanut seeds crude protein extract, underscoring the reasons for our choice of these two homologues in this study. Further, Kulis et al. (2012)[21] and Bernard et al. (2015)[17] have emphasized the attractiveness of these homologues in therapeutic applications.

In addition to *Ara h 2* and *Ara h 6*, the 2S albumin family is represented by *Ara h 7*, a less well-known allergen immunologically, with three isoforms reported in the literature [22–24]. Kelber-Janke et al.[23] detected sensitization to the *Ara h 7*.*0101* isoform in 43% of peanut-sensitive subjects compared to 85% for *Ara h 2*. In an immunoassay study focusing on peanuts allergen reactions in three groups of patients, Cordreanu et al.[25] showed that only 43% of patients were sensitive to *Ara h 7*.*0101* compared with 96% and 92% were sensitive to *Ara h 2* and *Ara h 6*, respectively. Therefore, although Ara h 7 is an albumin-based allergen, it is not a major allergen compared with *Ara h 2* and *Ara h 6*.

The peanut crop, *A*. *hypogaea*, is an allotetraploid comprising two genomes, AABB, with the A genome being contributed by *A*. *duranensis* and the B genome by *A*. *ipaensis* [26–28]. Our knowledge of allergenicity [29] in peanuts has been limited to the resolution of the primary and tertiary structures in the allotetraploid peanut crop species *A*. *hypogaea* and the two putative diploid progenitors [14, 28, 30]. Although the current knowledge represents a valuable foundation, it is limited and constrained by the strong sequence similarities among the allergen proteins of these three highly related species.

Herein, we examine *Ara h 2* and *Ara h 6* proteins of representative species from across the genus *Arachis* and seek to provide an in-depth understanding of the potential impact on structure and allergenicity of point mutations and loss/gain of sequence motifs, particularly those in the immunodominant epitopes. A number of studies have demonstrated that a single residue mutational event could significantly impact allergenicity in peanut species [14, 30–32]. For example, in *Ara h 2*, a targeted single amino acid mutation can result in a non-IgE binding epitope [14], and polymorphism in immunodominant epitope 7 in *A*. *duranensis* population resulted in 59–99% reduction in IgE binding activity [30]. Similarly, an increase in number of immunodominant epitopes is correlated with higher degrees of allergenicity[17]. Using newly generated nucleotide sequences of *Ara h 2* and *Ara h 6* from 24 of the approximately 80 *Arachis* wild species from clades spanning the entire tree, we determined naturally-occurring single nucleotide polymorphism (SNPs) and loss/gain of sequence motifs in the open reading frames (ORF) and their subsequent impact on amino acid composition. We placed these mutational events within a phylogenetic platform using the a phylogenetic tree of Friend et al. (2010)[33] to evaluate their modes of evolution, and determined potential selection pressure operating on them. To test the impact of mutational events on immunological reactivity, we conducted immunoblot experiments using *Ara h 2* antigens from selected *Arachis* wild species. Further, we identified epitopes, generated tertiary structures for species with prominent mutations using homology modeling, and mapped relevant epitopes to the protein structures via sequence alignments. In total, this work provides new allergen protein sequence data for *Arachis* species and begins to connect this sequence information to protein structure and subsequently to allergenicity within a predicted evolutionary framework.

## Materials and methods

### Genomic DNA extraction, gene amplification and sequencing

**Fig 1:**
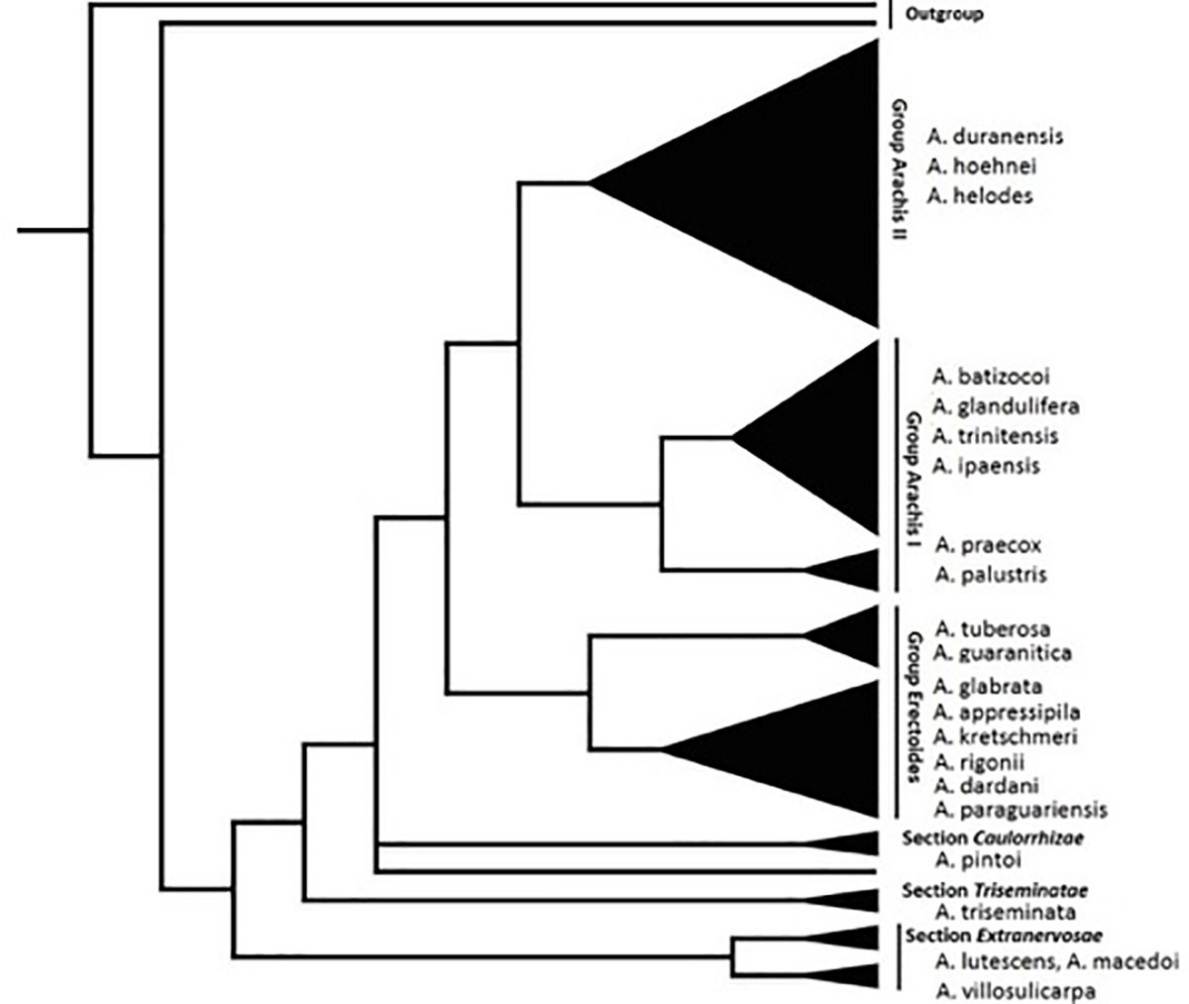
A summarized phylogeny of the genus Arachis based on a Bayesian inference of ITS sequence data (Friend et al. (2010) [33]. Labeled triangles represent monophyletic groups with species examined in this study noted. Lineages received 1.0 posterior probability support except for Group Arachis II that received 0.97 support. Arachis hypogaea is represented by two cloned alleles corresponding to its respective progenitors A. duranensis and A. ipaensis.

Genomic DNA was isolated from fresh leaf material of 24 *Arachis* species (S1 Table) following Milla et al. (2005)[34]. Those species represent all the phylogenetic lineages of the genus depicted in Friend et al. (2010, summarized in Fig 1)[33]. *Ara h 2* was amplified from 24 species and *Ara h 6* from 23 species (*Ara h 6* amplification failed in *A*. *tubersosa*) using the polymerase chain reaction (PCR) method with primers designed based on a sequence from *A*. *hypogaea* (GenBank accession AY007229). The *Ara h 2* open reading frame (ORF) was amplified with primers ARAH2STARt (5’ ATGGCCAAGCTCACCATA 3’) and ARAH2-526 (5’ AGCTTGCCTTAGTTAACACG 3’), whereas the *Ara h 6* ORF was amplified with primers ARAH6F (5’ ATGGCCAAGTCCACCATC 3’) and ARAH6R 556 (5’ CTATCACCACATTCATACAG 3’). Amplifications were conducted with 1x ThermoPol Buffer (New England Biolabs, Ipswich, MA), 200 μM dNTPs, 20 pmol of each primer, and 1.5 U *Taq* DNA polymerase (New England Biolabs, Ipswich, MA) in 25 μl reactions. PCR amplifications were carried out in a PTC-100 thermocycler (MJ Research, Waltham, MA) with 2 minutes of initial denaturing at 94°C, 25 cycles of 2 minutes denaturing at 94°C, 1 minute of primer annealing at 54°C, and 90 seconds extension at 72°C, followed by a final extension of 5 minutes at 72°C. Preparations of the PCR sequence reaction mixtures for *Ara h 6* amplification followed the same protocol as in *Ara h 2*. The PCR conditions were 2 minutes of initial denaturing at 94°C, 39 cycles of 2 minutes denaturing at 94°C, 1 minute of primer annealing at 51°C, 90 seconds extension at 72°C, and a final extension of 15 minutes at 72°C. PCR product cleaning and sequencing followed Voshell et al. (2011)[35].

### Sequence alignment

Nucleotide sequences in the phenograms were examined and manually edited in 4Peaks. Multiple alignments for *Ara h 2* and *Ara h 6* were generated initially using MUSCLE software [36] on CIPRES Science Gateway portal V. 3.3 (https://www.phylo.org/). The *Ara h 6* alignment did not require manual editing whereas the *Ara h 6* alignment was manually edited for accuracy in QuickAlign v. 1.03 [37] due to the presence of a large number gaps and and introduced frame shifts. Indels (insertions/deletions) were introduced in the alignment at a minimal cost of two substitutions [38] with special attention being paid to maintaining ORF integrity. The nucleotide alignments were translated into amino acids in MacClade Version 4.08a [39].

### Amino acid and side chain composition

MEGA 6 [40] was used to determine the nucleotide composition of the *Ara h 2* and *Ara h 6* data sets. Excluded from both nucleotide and amino acid sequence composition analyses were *A*. *helodes*, *A*. *trinitenses*, *and A*. *monticola* to avoid data skewing since these nucleotide sequences start at position 103 compared with the other *Arachis* species. To avoid data duplication, we also excluded the allotetraploid *A*. *hypogaea* because its genomic composition is represented by the progenitors *A*. *ipaensis* and *A*. *duranensis*. To generate completely overlapping data sets of 19 species, we excluded *A*. *tuberosa* in *Ara h 2* due to lack of an *Ara h 6* sequence.

The frequencies and percentages of amino acids were computed for the two alleles across the 19 *Arachis* species using software on the website of the Department of Chemistry, Central Connecticut State University (http://db.systemsbiology.net:8080/proteomicsToolkit/IsotopeServlet.html). To compensate for the differences in ORF lengths of *Ara h 2* and *Ara h 6*, the percentage values were used in determining side chain compositions. We followed Kyte and Doolittle (1982) [41] in assigning amino acids to their respective five groups: Hydrophilic and Uncharged: H, N, Q, S, T; Hydrophobic: A, C, G, I, L, M, P, V; Aromatic: W, Y, F; Charged, Acidic: D, E; Charged, Basic: K, R. The means, standard deviations and statistical *p* values for significance of difference were calculated in Microsoft Excel version 15.36, 2017.

### Sequence identity

The SIAS software of the Immunomedicine Group, Universidad Complutense, Madrid (SIAS, http://imed.med.ucm.es/Tools/sias.html) was used for the sequence identity analyses of nucleotide and amino acids. The Sequence Identity (ID) module calculates pairwise sequence identity percentages from multiple sequence alignments using the equation: ID = Identical Residues/Sequence Length x 100. Aligned matrices were used as input files and the Smallest-Length Sequences option was chosen. Gaps were included in the computations.

### Assessment of molecular evolution

To assess potential signature of natural selection on *Ara h 2* and *Ara h 6*, three metrics were used and compared for evidence of selection pressure signature. In one, we used the proportions of synonymous (dS) and nonsynonymous (dN) mutations as an estimated measure of ω as computed in SNAP [42]; www.hiv.lanl.gov). This computation follows the distance-based method of Nei and Gojobori (1986) [43] which estimates the numbers of dS and dN substitutions per site implementing equal weight for all codon substitutions, and incorporates a statistic developed by Ota and Nei (1994) [44]. Computed here are average behaviors of each codon in pairwise comparisons for indels, synonymous and nonsynonymous mutations.

We further explored two other matrices using HyPhy as implemented in Datamonkey portal (http://www.datamonkey.org): one assesses selection at a gene-wide scope while the other determines selection signatures at a per-site level. BUSTED (Branch-Site Unrestricted Statistical Test for Episodic Diversification [45] was used to test for gene-wide (not site-specific) positive selection. All branches in the tree were selected as the foreground for testing natural selection on the entire phylogeny. BUSTED fits a codon model with three rate classes (ω_*1*_ ≤ ω_*2*_ ≤ ω_*3*_, unconstrained model) and tests for positive selection against a constrained null model (ω = 1, disallowing positive selection) on the foreground branches. We used FUBAR (Fast, Unconstrained, Bayesian Approximation [46] to test positive and negative selection at a per-site basis. FUBAR uses the Bayesian algorithm and the posterior probabilities to infer dN and dS on a per-site basis using a provided phylogeny. The method assumes that selection pressure at each site is constant along the entire phylogeny. Maximum likelihood analysis was carried out using RAxML and executed in the CIPRES portal (www.phylo.org) implementing the default options. The phylogeny generated was used in the above analyses for testing evidence of natural selection. Since we do not have sequences for an outgroup species to root the tree, we opted to use *A*. *macedoi* for rooting the tree because it emerged at the base of the *Arachis* phylogeny in the Friend et al. 2010 study [33].

### Characterization and structural modeling of *Ara h 2* and *Ara h 6*

Secondary structure prediction and analysis was performed for all *Ara h 2* and *Ara h 6* sequences via the PSIPRED Protein Sequence Analysis Workbench [47]; (bioinf.cs.ucl.ac.uk/psipred). Prediction methods utilized include PSIPRED v.3.3, DISOPRED3, DOMPRED, and FFPred v3. This analysis suite provided insight into secondary structure and function prediction, disordered regions, and disordered protein binding regions for all sequences.

Homology models were generated for *A*. *hypogaea* 2.01 and 2.02, and *A*. *triseminata* in order to map the predicted epitope regions to structure. We chose to model the two isoforms *Ara h* 2.01 and *Ara h 2*.*02* to represent the allergenic components of the peanut crop and selected *A*. *triseminata* because of its basal phylogenetic position and lack of the immunodominant epitopes. Conformer 1 of the NMR structure of *Ara h 6* from *A*. *hypogaea* was used as template (PDB ID: 1W2Q, [37]). We opted to use the Ara h 6 NMR structure despite the availability of a crystal structure for *Ara h 2* because the *Ara h 2* crystal structure (PDB ID: 3OB4) is based on a fusion of maltose binding protein (MBP) with *Ara h 2* and lacks the region containing epitopes 6 and 7 of this allergen. This immunologically important region is present in the NMR structure of *Ara h 6*. The missing residues in the *Ara h 2* crystal structure (see PDB ID: 3OB4, 1057–1083 missing residues) were essential to our analysis of epitopes 6 and 7, which was a factor that influenced our template choice. An overlay of the available template structures and the model created are shown in the supplemental material and highlight the influence of the missing residues of epitope 6 and 7 on the Ara h 2 model structure, further justifying the selection of the Ara h 6 template for generating the model. Barre (2005) [15] and *Power* (2012) [48] generated homology models of *Ara h 2* using the castor bean allergen Ric c 3 as template, but Mueller et al. (2011) [49] illustrated that *Ara h 2* is a better structural match to *Ara h 6* than to the Ric c 3 allergen, thereby further validating our approach.

The homology models of *A*. *hypogaea Ara h 2*.*01* and *Ara h 2*.*02*, and *A*. *triseminata* were built and energy minimized in Molecular Operating Environment (MOE) [50] with Amber12EHT [51] parameters and validated with ProSA [52], Ramachandran plots [53], ANOLEA [54], and QMEAN using ProSA-web [55], RAMPAGE [53], and the SwissModel [56] suite of tools. We are confident in the models given the structure validation analysis metrics showed that all three models had favorable energy throughout most of the proteins based on ANOLEA, with minimal regions of unfavorable energy in the N-terminal region and in loop regions (residues 50–60, 115–120). Ramachandran plots indicated most residues had favorable dihedral angles (*A*. *hypogaea* 2.01–98%, *A*. *hypogaea* 2.02–76%, and *A*. *triseminata* - 78%). Overall protein structure z-scores, which consider torsion, side chain position, and overall fitness, fell into a range that indicated a similarity to known, high-quality NMR protein structures for all three structures. To relate potential tertiary structure and epitope placement, we used DiscoTope 2.0 [57] to predict linear and conformational epitopes based on the 3D structures of *Ara h 2* from *A*. *hypogaea Ara h 2*.*01* and *Ara h 2*.*02*, and *A*. *triseminata* [58, 59]. DiscoTope predictions combine computational methods proposed by Nishikawa and Ooi (1980) [60] to measure the amino acids located at the protein surface. Additionally, the NACCESS program [61] has been incorporated with DiscoTope to determine the residues that are accessible to the solvent.

### Dot immunoblots for epitopes of conglutin proteins

Seven peanut-allergic and four non-allergic subjects (18–34 years old) were recruited. Serum samples were isolated by centrifugation at 3000 rpm for 10 min from the 10 mL of whole blood donated by subjects and stored at -80° C until use. Among the 10 IgE-binding epitopes identified *for Ara h 2* [14], epitopes 6 and 7 contain the immunodominant DPYSPS motif, and thus we focused on these two epitopes in this study. We generated 15–18 oligomer peptides (GenScript, Piscataway, NJ) to represent the portions with the DPYSPS motif or its variants. As a control, we used purified *Ara h 2* protein derived from *A*. *hypogaea* contributed by Soheila Maleki (USDA, New Orleans, LA). Hydrated peptides were spotted directly onto Protan nitrocellulose membrane and allowed to dry. Dot immunoblots were incubated in 5% non-fat milk in Tris-buffered saline with 0.5% Tween-20 (TBS-T; pH 7.5) for one hour at room temperature with shaking. The blots were incubated overnight at 4°C in either 1:6000 diluted chicken α-*Ara h 2* antibody or in 1:20 diluted sera from peanut allergic individuals and washed three times with TBS-T for 10 min after antibody incubation. The dot immunoblots probed with chicken α-*Ara h 2* antibodies were subsequently incubated with rabbit anti-chicken IgG horseradish peroxidase (HRP)-conjugated antibodies (1:2500; Bethyl Laboratories Inc., Montgomery, TX) for one hour at room temperature. The immunoblots incubated with human sera were incubated with rabbit anti-human IgE HRP-conjugated antibodies (1:1000; Bethyl Laboratories Inc.). ECL Plus detection reagents were used for the HRP-conjugated secondary antibodies for chemiluminescent detection with a Fuji LAS-300 camera using the FUJIFILM Multi Gauge version 3.X.

## Results and discussion

**Fig 2:**
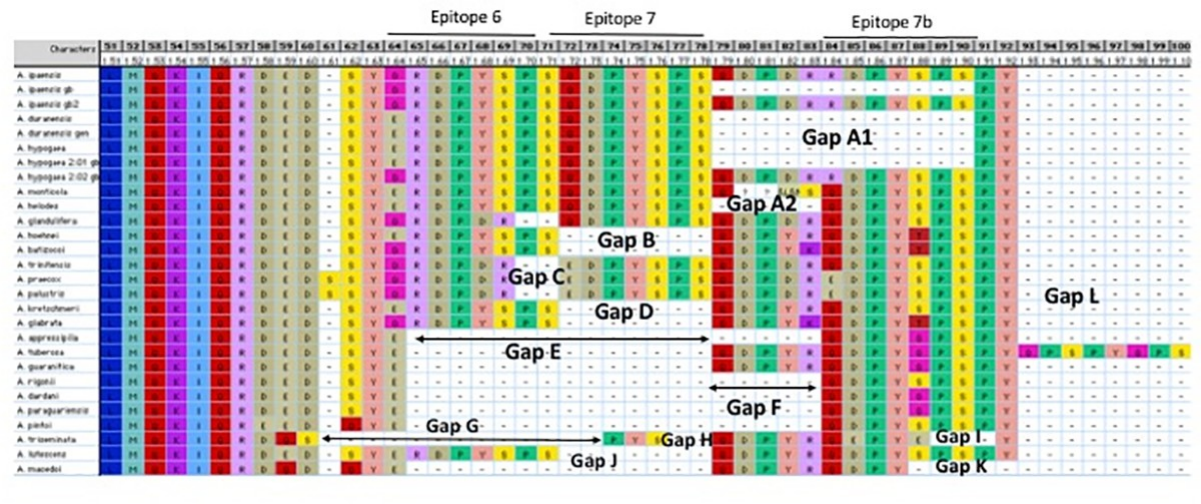
*Ara h 2* amino acid alignment section containing the immunodominant epitopes 6, 7 and 7b. Gaps and epitopes are labeled. Numbers above the columns denote alignment positions.

Since our knowledge on peanut allergy has been limited to the crop species and its two genomic ancestors, and considering the findings that single amino acid substitutions could have substantial impact on allergenicity [14, 31, 32], it is pertinent to explore single amino acid substitutions in the wild species of *Arachis* and evaluate their impact on allergenicity. To do so, we sequenced the two immunodominant allergens *Ara h 2* and *Ara h* 6 from 24 species representing the major lineages in *Arachis* and used multiple approaches to characterize molecular alterations and determine their impact on allerginicity. Three striking outcomes from this study have emerged. One, the two allergen homologues studied, *Ara h 2* and *Ara h 6*, followed very contrasting evolutionary pathways. Specifically, *Ara h 2* has undergone a higher proportion of amino acid substitutions compared with *Ara h 6* and has accumulated by far a greater number of losses and gains of motifs ranging from 1–24 amino acids (S1 Fig). Two, and quite importantly, these molecular alterations were mostly concentrated in the immunodominant epitope-rich regions, specifically in Epitopes 6 and 7 (Fig 2, S1 Fig). Three, the mutational events (substitutions and insertions/deletions) particularly in *Ara h 2* appear to follow phylogenetic trends from the base of the *Arachis* tree to the terminal branches. These findings raise the questions of the underlying factors that differently affected *Ara h 2* and *Ara h 6* modes of evolution and whether such molecular alterations are linked to potential accentuation of allergenicity. To address this curious biological situation, we will compare the two homologues at various molecular tiers and discuss the findings in a phylogenetic/evolutionary framework.

### Comparative molecular composition across the genus *Arachis*

**Fig 3:**
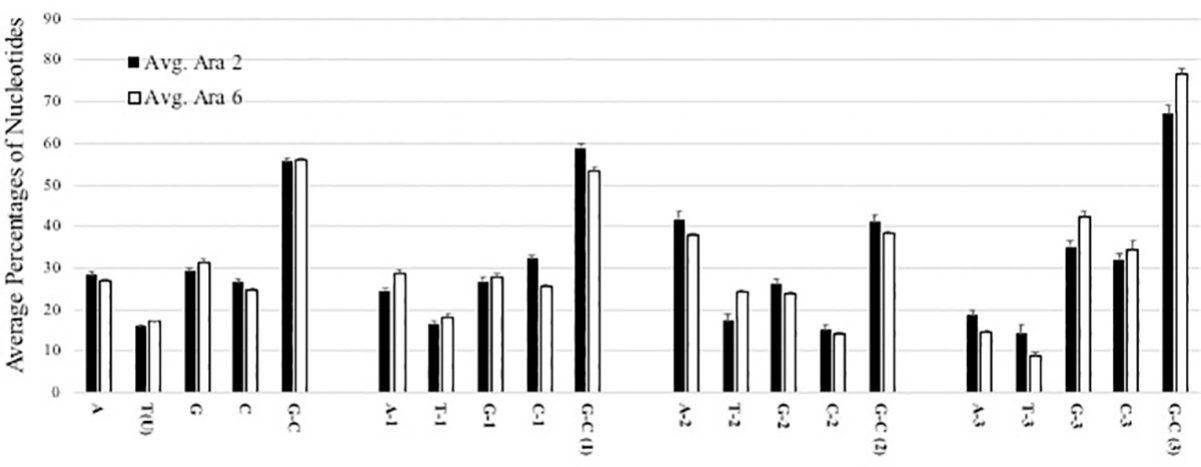
Average percentages of Adenine (A), Thymine (T), Guanine (G), Cytosine, and GC ratios. Calculations were done for overall DNA sequences and individual codon positions of *Ara h 2* and *Ara h 6* from the Arachis species. The notations 1, 2 and 3 denote 1^st^, 2^nd^, and 3^rd^ codon positions. Data are presented as mean ± SD. A two-way ANOVA with Sidak post-test was applied. The two homologues were not statistically different in nucleotide composition.

One of the prominent findings in this study is that *Ara h 2* and *Ara h 6* homologs from wild *Arachis* species differ substantially in mutational patterns and ORF length. To shed light on the underlying factors that might account for these differences and assess the impact of this contrasting mode of evolution on these proteins, we evaluated molecular compositions at two tiers: nucleotide and amino acid levels. The nucleotide compositions for the entire ORFs of *Ara h 2* and *Ara h 6* homologs as well as the nucleotide frequencies per codon position are presented in Fig 3. Overall, the two homologues did not differ significantly in their nucleotide composition across the genus since the t-test rejected the null hypothesis. This underscores notable constraints on their mode of nucleotide substitutions to maintain similar compositions.

It has been demonstrated that higher GC content correlates positively with rates of substitutions [62, 63]. A recent study focusing on *A*. *duranensis* estimated an average GC content of 31.8% for the whole genome, which is in line with most legumes examined [9]. The overall GC ratio for both homologues was highly similar (*Ara h 2* = 55.7± 0.8 *Ara h 6* = 56.1± 0.5, (Fig 3). The average 56% GC content of *Ara h 2* and *Ara h 6*, being substantially higher (75%) than that reported for the whole *A*. *duranensis* genome points to a suitable genetic landscape for accelerated rates of mutations in both. Nevertheless, the frequency of amino acid substitutions remained lower in *Ara h 6*. One notable difference is that *Ara h 2* exhibited relatively higher GC ratios in its 1^st^ and 2^nd^ codon positions contrasted with *Ara h 6* (Fig 3). This difference may account in part for the elevated amino acid substitutions in *Ara h 2* since mutations in 1^st^ and 2^nd^ codon positions are translated into 96% and 100% nonsynonymous substitutions, respectively, compared with 31% for the 3^rd^ position [64].

**Fig 4:**
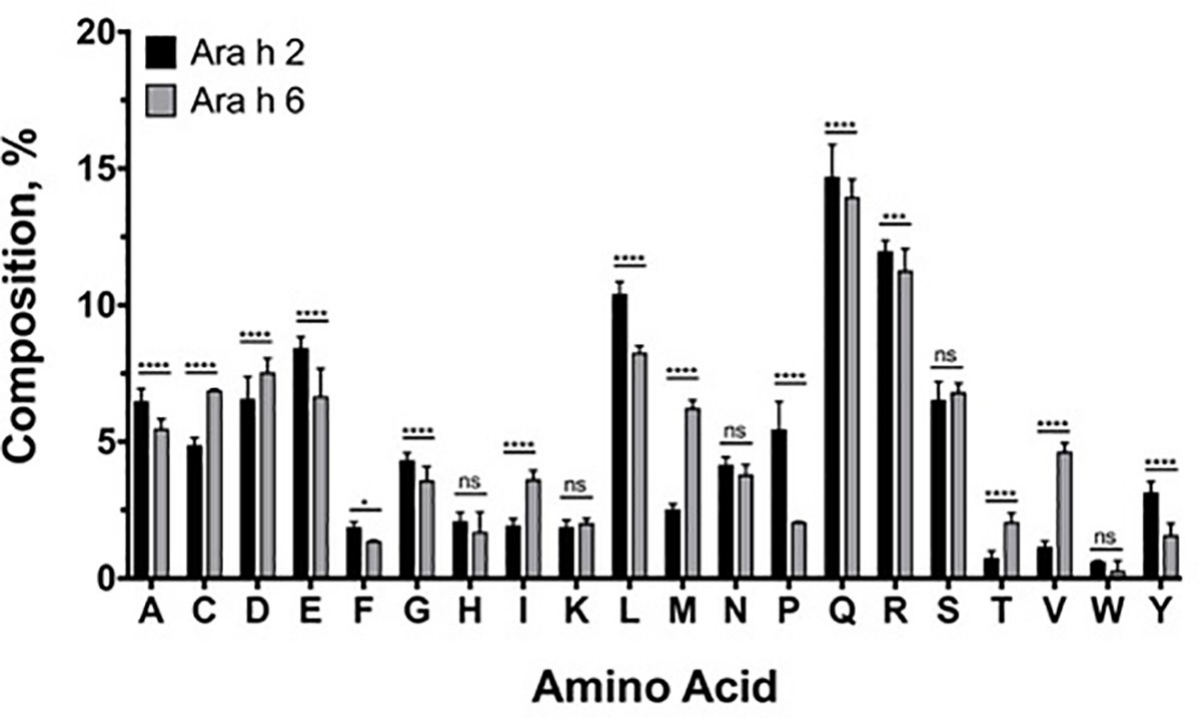
Percent deduced amino acid composition of Ara h 2 and Ara h 6 proteins from the *Arachis* species. A two-way ANOVA with Sidak post-test was applied. All data are presented as mean ± SD. *p < 0.05, **p < 0.01, ***p < 0.001, ****p<0.0001.

The two homologues, although being highly allergenic, display disparity in their number and modes of amino acid substitutions (Fig 4, S1 Fig). Reciprocal amino acid sequence identities for *Ara h 2* across the *Arachis* species ranged between 65.5% to 99.4% (mean 89.4%± 8.2) compared with 75.7%-99.3% for *Ara h* 6 (mean 93.2%± 5.3), underscoring the higher frequency of substitutions in the former. In the case of *Ara h 2*, the ORF length ranges from 156 to 181 amino acids (S1 Table). The amino acid alignment of *Ara h 2* required the insertion of 17 gaps ranging from 1 to 29 amino acids, resulting in 203 alignment positions (S1 Fig). Importantly, amino acid positions 59–90 in epitope 5 and the immunodominant epitopes 6, 7 and the newly recognized 7b display residue substitutions and losses/gains that may potentially impact allergenicity (Fig 2). Thirteen gaps in the sector that includes epitopes 6, 7 and 7b are identified and labeled A-L (Fig 2). The mutation in position 64 presents a molecular marker for the genomic ancestry of the tetraploid crop since it is a glycine (G) in all three accessions of *A*. *ipaensis* and glutamic acid (E) in all *A*. *hypogaea* and *A*. *duranensis* accessions (Fig 2).

The *Ara h 6* ORF, in comparison, displayed a narrower range of amino acid residues across the genus, 145 to 147 (S1 Table). Only two sequence gaps were required for the alignment, corresponding to a methionine at position 22 and an arginine at position 69 (S1 Fig). Interestingly, this apparent in-frame initiation codon at position 22 evolved in species that emerged after the early diverging *A*. *triseminata*, *A*. *paraguariensis* and *A*. *macedoi*. This methionine residue could not be considered as an alternate initiation codon since it occurs downstream of the signal peptide region. Remaining amino acid differences were due to replacements, underscoring the contrasting patterns of evolution undergone by *Ara h 2* and *Ara h 6* across the genus. The infrequent loss/gain of amino acid residues in *Ara h 6* across the genus *Arachis* compared with *Ara h 2* is intriguing and could be attributed to structural/functional limitations on this allergen protein.

**Fig 5:**
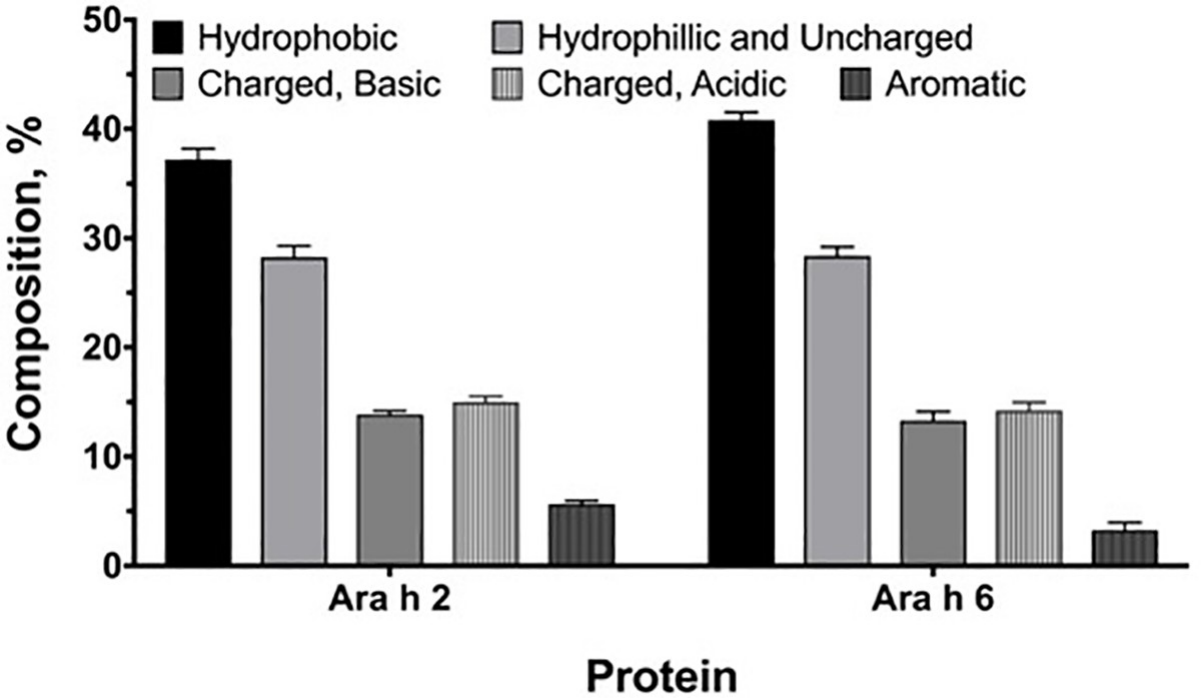
Percentages of side chain composition of deduced amino acids in *Ara h 2* and *Ara h 6* proteins. The means, standard deviations (noted as bars) and the statistical p values for significance of difference were calculated in Graphpad Prism. A two-way ANOVA with Sidak post-test was applied. The two homologues were not statistically different in sidechain compositions.

To assess potential heterogeneity in amino acid composition across the *Arachis* genus evolutionary history due to the noted mutational events, we analyzed amino acid compositions of the *Ara h 2* and *Ara h 6* homologs. The two were comparable in amino acid composition across the genus, and were low in tryptophan (W) and high in arginine (R) and glutamine (Q) (Fig 4 and S2 Table). These findings are in agreement with previous studies on peanuts [65]. Tryptophan was lacking in *Ara h 2* of three species and the *Ara h 6* of 14 species (S2 Table). It is observed that *Ara h 2* accommodated higher variation across the genus *Arachis* in glutamine (Q), proline (P), and aspartate (D), whereas *Ara h 6* displayed higher variation in glutamate (E), histidine (H) and arginine (R) (S2 Fig and S2 Table). Despite the contrasting patterns of variability, the two homologues are similar in composition in terms of the five amino acid physicochemical groups and they are rich in hydrophobic amino acids and low in aromatic amino acids (Fig 5). Therefore, mutational events in the two homologues appear to be constrained to maintain the physicochemical groups composition in order to conserve the functional and structural integrity of these proteins.

**Fig 6:**
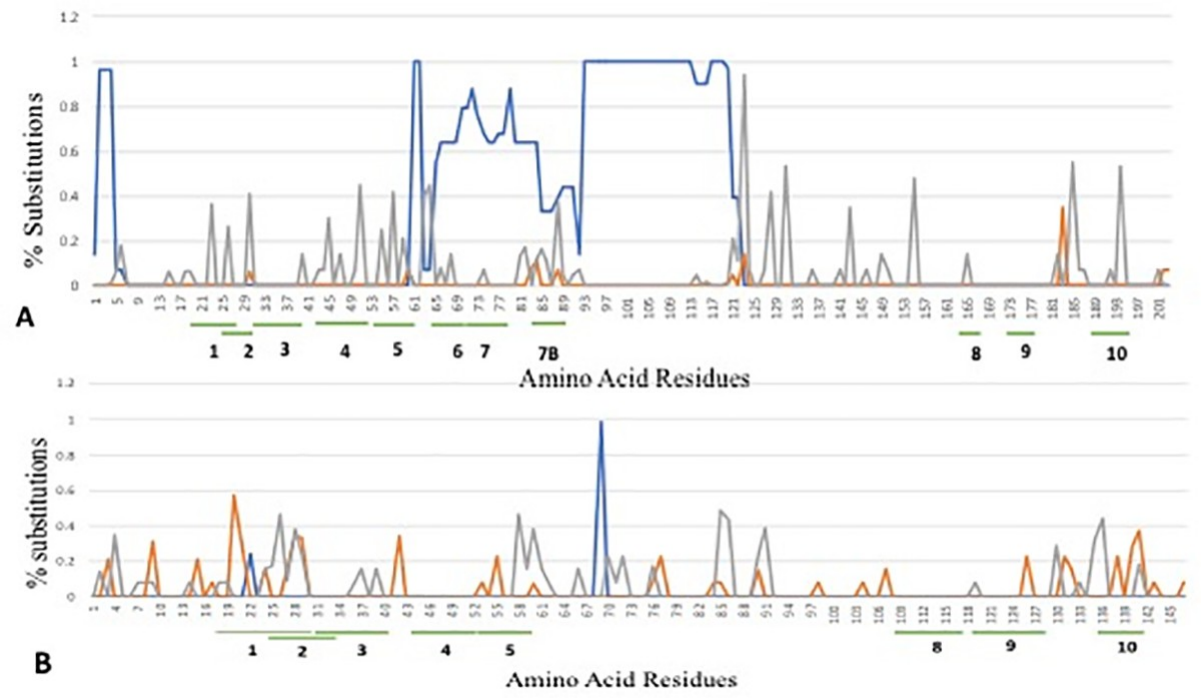
SNAP analysis of *Ara h 2* and *Ara h 6* amino acid sequences. Shown are a per-codon distribution of synonymous mutations (red), nonsynonymous mutations (gray) and indels (blue). IgE-binding epitopes are highlighted beneath the amino acid residues. 5A. Ara h 2 homologue. 5B. Ara h 6 homologue.

The hydrophobic nature of the protein is important for the functionality of the signal peptides of these allergens [30]. *Ara h 2* and *Ara h 6* are synthesized in the cytoplasm as precursor proteins with an additional N-terminal amino acid signal peptide required for transport to the vacuoles [19, 30, 66]. The signal peptides (amino acid residues 1–7 [29] or residues 1–21 [67] contain 2-residue gaps in *Ara h 2* and display a number of substitutions in both *Ara h 2* and *Ara h 6* (Fig 6 and S1 Fig). Despite these mutations, the region remained highly constrained to maintain the hydrophobic physicochemical property required for its crucial function (S1 Fig). In summary, although the two allergen proteins have undergone contrasting patterns of amino acid substitutions and losses/gains over the evolutionary history of the genus, they maintained comparable amino acid composition and physicochemical groups.

### Signatures of natural selection in *Ara h 2* and *Ara h 6*

The contrasting mutational patterns observed in *Ara h 2* and *Ara h 6* (Figs 4 and 6, S1 Fig) raise the question of whether these evolutionary modes are driven by natural selection, positive-diversifying or negative-purifying. Assessment of signatures of natural selection operating on coding genomic regions is a major challenge. Murrell et al. (2015) [45] noted that there is not yet an uncontroversial way to answer the question of whether a gene evolved under positive selection. Underlying factors that contribute to the difficulties in determining positive selection signature include intrinsic patterns of gene substitutions, biological assumptions made, and models used. To assess potential natural selection signatures acting on *Ara h 2* and *Ara h 6*, given the prominent differences in mutational patterns, we selected and contrasted results of three independent methods (distance–based, BUSTED, and FUBAR) that adopt different biological assumptions and use metrics for site-specific or whole gene estimation of natural selection signatures.

One of the simplest methods is that of Nei and Gojobori (1986) [43] that focuses on the detection of natural selection using the rates of synonymous vs. nonsynonymous substitutions in protein coding genes. The algorithms compute variance and covariance of dS (synonymous) and dN (nonsynonymous) mutations and the proportions of synonymous (pS) and nonsynonymous (pN) differences. Using SNAP for these computations, the averages of all pairwise comparisons for the *Ara h 2* data set showed dS = 0.0142, dN = 0.0475, dS/dN = 0.3319, and pS/pN = 0.3379. In contrast, the *Ara h 6* data set revealed dS = 0.0831, dN = 0.0247, dS/dN = 5.8627, and pS/pN = 5.5914. The 0.332 dS/dN for *Ara h 2* implies that it has experienced positive selection along the genus evolutionary history whereas the 5.863 dS/dN calculated for *Ara h 6* indicates purifying selection. A dS/dN value below 1 implies positive/diversifying selection while above 1 signifies purifying selection (*SNAP output is presented as dS/dN not dN/dS*). BUSTED, a gene-wide and positive selection-focused method, reported evidence of gene-wide episodic diversifying selection in *Ara h 2* across the branches of the phylogeny (*p* = 0.005 ≤ 0.05) but no evidence of episodic diversifying selection in *Ara h 6* (*p*-value = 0.151 ≥ 0.05). Therefore, the BUSTED finding is in agreement with the Nei and Gojobori (1986) approach, with both showing that *Ara h 2* is experiencing positive selection. *Ara h 6*, on the other hand, was shown to be under purifying selection using Nei and Gojobori (1986) approach (BUSTED tests for positive selection).

The site-specific, Bayesian-based FUBAR method detected in *Ara h 2* diversifying selection and purifying selection at 5 sites each with 0.9 posterior probability. For *Ara h 6*, this method detected diversifying selection at 2 sites but purifying selection at 8 sites (posterior probability 0.9). The detected sites are highlighted in S3 Table. FUBAR (Murrell et al. 2013) [46] has more power for detecting weak positive selection (at low *ω*>1 values). Contrasting the gene-wide and the site-specific methods used here, it is believed that the gene wide methods are more effective in detecting natural selection signatures. Murrell et al. (2013) [46] noted that when positive selection is undetectable on any one codon site or branch in isolation, pooling evidence for positive selection across multiple sites and branches can render their cumulative effect more evident. Similar inferences were reached by others [68, 69]. This seems to be the case in the detection of positive selection by the gene-wide methods used in this study. In conclusion, the gene-wide approaches have shown that *Ara h 2* is evolving under positive/diversifying selection. The Nei and Gojobori (1986) [43] and the FUBAR approaches (BUSTED tests for positive-selection) demonstrated that *Ara h 6* is evolving under negative/purifying selection. FUBAR detected sites under positive selection in both *Ara h 2* and *Ara h 6* but more sites under purifying selection in *Ara h 6* (2 vs. 8 sites, respectively). These findings raise the curious questions of whether positive selection in *Ara h 2* played a role in the emergence of immunodominant motifs, whereas purifying selection in *Ara h 6* maintained a status que for a gene that has emerged initially with strong allergenic features when *Arachis* diverged from its common ancestry.

To evaluate the potential consequences of the positive selection detected in *Ara h 2*, two concepts will need to be addressed. One relates to the detection of progressive increase in allergenicity across the evolutionary history of the genus and the other pertains to its potential adaptive advantages for allergenicity. Insight into the relationship between the progressive expansion of the region encompassing the immunodominant epitopes 6, 7 and 7b, including the progressive additions of DPYSPS motifs in *Ara h 2* (Fig 2) and the enhancement of allergenicity is provided by the immunoblot experiments. These experiments (detailed later) revealed a progressive increase in allergenicity from the basal species *A*. *triseminata* to the terminal species in the phylogenetic tree, *A*. *hypogaea*. The changes correspond to expansions of the immunodominant motifs region (Fig 2), pointing to a possible association between the two phenomena. The second point to be addressed is the availability of evidence for adaptive advantages allergenicity may confer on peanut seeds. Direct experimental studies addressing this question in the wild species of *Arachis* are unavailable in the literature. However, some work exists that links the allergen proteins to defense against insects and pathogens. *Ara h 2* is a trypsin inhibitor [7] and this function has been shown to control insect attacks, a feature that is being used in breeding programs for insect tolerance during seed storage [70]. The 2S albumin protein family, to which *Ara h 2* and *Ara h 6* belong, was demonstrated to be effective in inhibiting the growth of fungi and bacteria in radish seeds and four other members of the mustard family Brassicaceae [71]. Similarly, antifungal properties are known for a 2S albumin-homologous protein from passion fruit seeds [72]. Considering these findings in peanuts and other flowering plants, positive/diversifying selection pressure that promotes the augmentation of allergenicity may be advantageous to increase the chances of seed survival via protection from insects and fungi. The patterns of molecular evolution documented in *Ara h 2* and the corresponding progressive increase in allergenicity resolved in the immunoblots encourages direct studies on wild species seeds to evaluate selective advantages for protein allergenicity.

### Phylogenetic and evolutionary assessment of epitopes

The pattern of substitution (mutations and indels), particularly nonsynonymous substitutions, could have dual structural and functional implications in protein coding genes. In allergen genes, nonsynonymous substitutions in epitope-rich sections or duplications of allergenic epitopes may substantially impact allergenicity [14, 17, 30]. It has been shown in peanuts that a single residue substitution expresses up to 99% reduction in allergenicity by impacting the IgE-binding ability [14, 31, 32]. We will discuss within a phylogenetic platform the mutational events in the allergenic epitopes of the two homologues. Identification of the *Ara h 2* and *Ara h 6* epitopes across the genus is based on epitopes recognized in the crop and its putative ancestors by Staley et al. (1997) and Ratnaparkhe et al. (2014) [14, 29].

### The *Ara h 2* homologue

**Fig 7:**
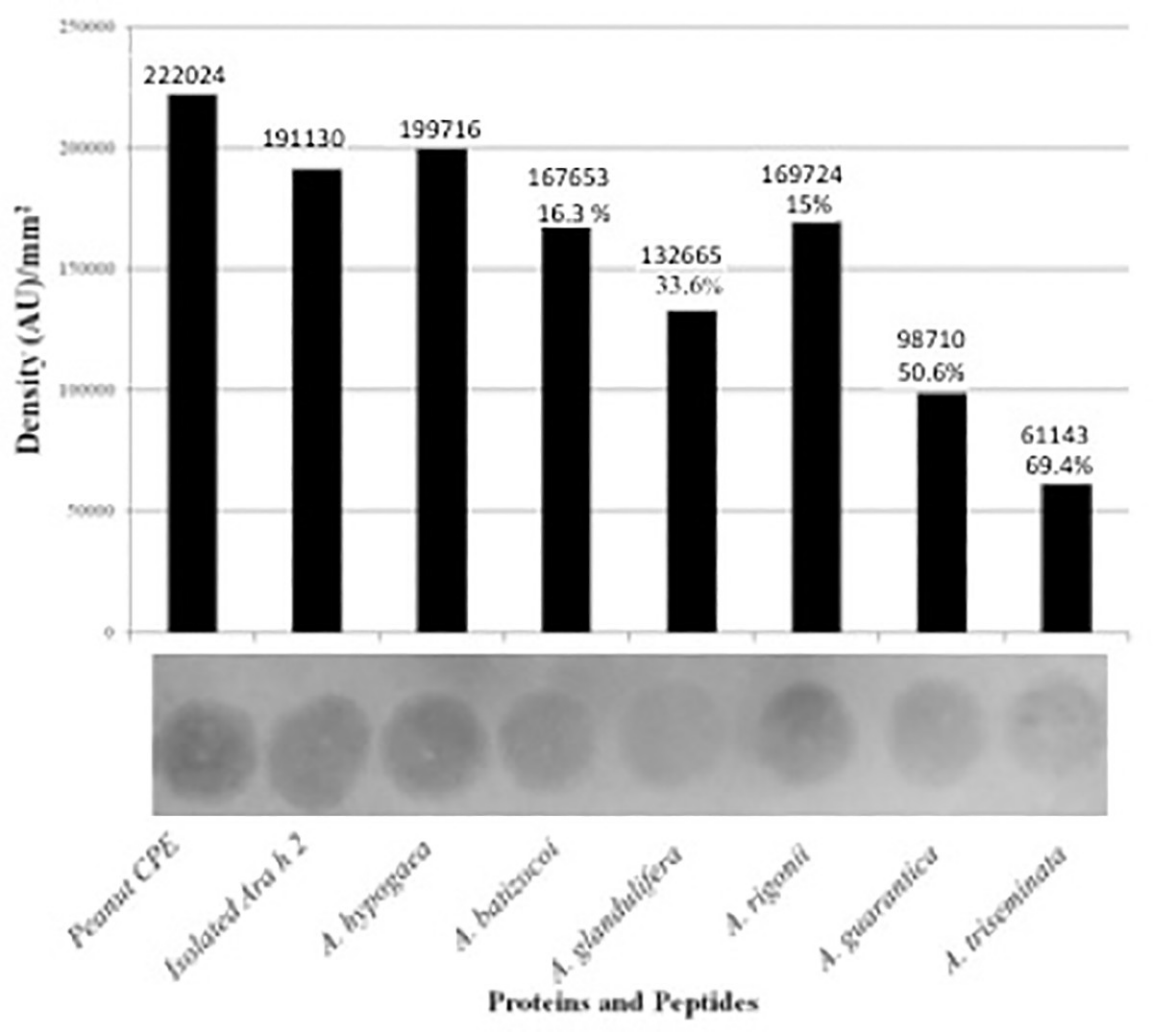
Dot Immunoblot for sera from peanut-allergic individual used to test peanuts crude protein extract (Peanut CPE), purified Ara h 2 protein, and synthetic peptides containing the hyperallergenic epitopes 6 and 7. Dot immunoblot on a nitrocellulose membrane strip is shown at the bottom and its densitometry measurements are represented as bars above the respective antigens with absolute absorption shown above bars. The synthesized polypeptides used were: A. hypogaea: RDPYSPSQDPYSPS, A. batizocoi RDPYSPSQDPYKQDP, A. glandulifera DPDRQDPYSPSQDPD, A. guarantica EQDPYRQDPYGPSPYG, A. rigonii EQDPYGPSPYGPSPY, A. triseminata QSPYSQDPYRQEPYE.

Ten epitopes have been recognized in *Ara h 2* [14]. Epitopes 6 and 7 are unique to this homologue and contain the characteristic immunodominant hexapeptide DPYSPS motif (Fig 2 and S1 Fig) located between helices 2 and 3. The other eight epitopes are split among the upstream region (epitope 1–5) and the downstream regions (epitopes 8–10). Epitope 1–4 (HASARQQWEL, QWELQGDR, DRRCQSQLER, LRPCEQHLMQ, respectively), and epitopes 8–10 (LQGRQQ, KRELRN, QRCDLDVE, respectively) are completely to highly conserved across *Arachis* (S1 Fig). Epitope 5 (KIQRDEDS), in contrast, has undergone complex mutational events. Although its KIQR motif is unchanged throughout the genus, the remaining sector (DEDS) has accommodated a number of mutations and indels across the genus history, appearing as DQD-Q and DED-S (dashes denote gaps) in the basal clade of *A*. *macedoi*, *A*. *lutescens*, and *A*. *triseminata*, mutating to DQS—, EED-Q, DEDSS in species of subsequent lineages, and reverting back to the original sequence of DED-S in the crop and its related species of the terminal lineage (Fig 1 and S1 Fig). It is to be noted that the early diverging *A*. *triseminata* with the truncated DQS—motif displayed the lowest degree of allergenicity in our immunoblot experiment compared with the peanut (Fig 7, discussed below). These natural mutations have the potential of providing valuable guidelines for examining the impact of loss/gain and residue mutations on allergenicity.

Epitopes 6 and 7 (RDPYSP and SQDPYSPS, respectively) contribute significantly to *Ara h 2* allergenicity [14]. We recognize within downstream epitope 7 another RDPYSPS motif (positions 84–90; Fig 2), a potential additional IgE-binding epitope, denoted epitope 7b. This motif was noted before but not recognized as an epitope [28, 30]. The three epitopes 6, 7, and 7b could have evolved via sequence duplications accompanied by SNPs. It has been reported that more than one motif of DPYSPS in the gene could present allergenic risk [17] and, therefore, the presence of three motifs would accentuate peanut allergy. Importantly, the region encompassing these three epitopes is punctuated with gaps and point mutations across the *Arachis*, revealing a progressive evolution of the three immunodominant epitopes and allergenicity in peanuts (Fig 2).

It is rather intriguing that in the first emerging clade (Section Extranervosae, Fig 1), *A*. *macedoi* lacks all three epitopes (Gaps E and K) whereas its sister species *A*. *lutescens* possesses epitopes 6 and 7b (Gap H, Fig 2, S1 Fig). A parsimonious explanation here is that 1) the presence of epitopes 6 and 7b is an ancestral state whereas their loss is a derived one, and 2) epitope 7 is a subsequent derivation from them. All three epitopes were lacking except for a few residues (Gaps G, H, I) in the next emerging species *A*. *triseminata* (Figs 1 and 2), whereas in the following clade, *A*. *pintoi* (Section Caulorrrhizae), only epitope 7b is maintained (Gaps E, F). The species of the next major lineage, Group Erectoides (Fig 1), again displayed a variety of losses, gains and SNPs that eliminated or modified these epitopes (Fig 2). Various residue losses (Gaps C, D, E, G) and amino acid mutational events either eliminated, truncated or highly modified epitope 6 (Figs 1 and 2). Losses and gains of these immunodominant epitopes early in the evolution of the genus could have had impact on allergenicity and, thus, present an excellent material for studying the molecular basis of allergenicity in peanuts. These molecular evolutionary scenarios in *Ara h 2* point to the existence of allergenicity at the advent of the genus *Arachis* albeit at relatively lower magnitudes.

### The *Ara h 6* homologue

*Ara h 6* accommodates only two, single amino acid residue gaps in the entire ORF located at amino acid residue positions 22 and 69 (S1 Fig). The gap at position 22 corresponds to a lack of a methionine in early-diverging *A*. *triseminata*, *A*. *macedoi* and *A*. *paraguarensis* (Fig 1, S1 Fig). As noted earlier, this methionine insertion is not expected to be involved in the transcription of the ORF since it will eliminate the crucial signal peptide region.

*Ara h 6* lacks the hexapeptide DPYSPS immunodominant motifs found in Ara h 2 and consequently, only eight epitopes have been recognized, 1–5 and 8–10 (S1 Fig). Epitope 1 (HASAMRRERGRQG) contains some conserved sections and others that experience mutations (Fig 6 and S1 Fig). Epitope 2 (ERGRQGDSSS) is highly conserved and epitopes 3, 4, 8 and 9 (DSSSCERQVDR, LKPCEQHIMQRI, RLQDRQMVQQ, and KRELMNLPQQ, respectively) remained entirely unchanged across the genus (S1 Fig), pointing to their structural and functional significance. In contrast, epitope 5 (RIMGEQE) exhibited substitution events in early diverging lineage followed by reversals in terminal lineages including the peanut species. Epitope 5 pattern mirrors that in *Ara h 2*. In epitope 10 (QRCDLDV), the first two N-terminal residues in the basal species *A*. *macedoi*, *A*. *triseminata* and *A*. *paraguariensis* (Fig 1 and S1 Fig) differ from the QR motif found consistently in species emerging upwards in the *Arachis* phylogenetic tree. It would be useful to investigate if these residue mutations and subsequent reversals from the early diverging species to the terminal ones signify possible selection to maintain allergenicity.

### The peanut crop and its ancestral species

Our sequence data and those in GenBank show two variants of *Ara h 2* in the diploid *A*. *ipaensis* (Fig 2). In one variant, epitope 7b lacks the DPYSPS motif (Gap A, residues 79–90) plus five upstream residues (GB accession XM_016314981.1) whereas in the other variant that whole region was maintained (XM_016314980.1 and ours). Since the peanut crop inherited two alleles of *Ara h 2* with and without epitope 7b, and one progenitor, *A*. *duranensis*, lacks this epitope, it appears that the *Ara h 2*.*02* allele was contributed by the *A*. *ipaensis* genotype possessing epitope 7b. It is rather remarkable that such a loss of a region containing an important allergenic epitope can take place in sister species within a relatively short historic time. Nevertheless, species containing all three epitopes (6, 7, and 7b) with their DPYSPS motifs represent an evolutionary climax of allergenicity in *Arachis*, which ironically is what we detect in the peanut crop. In conclusion, the prevalence of pronounced natural mutations in this sector of Ara h 2 provides insight into the link between amino acid mutations and allergenicity and encourages efforts of experimental genetic manipulation of allergenic regions in the crop.

### Immunoblot for *Ara h 2*

Given the pronounced differences in epitope composition among the *Arachis* species and its known strong allergenicity, *Ara h 2* was further probed for allergenic response based on pronounced mutational events that partially or completely eliminated some of its epitopes including the highly allergenic DPYSPS motif. We selected species from across the phylogenetic tree to assess within a phylogenetic background the impact of these major genetic transformations in *Ara h 2* on allergenicity using the immunoblot approach (Fig 7). The results from immunoblots probed with the various human peanut allergy-sensitive sera collected were highly similar and thus the dot immunoblot probed with sera from PA-5 was chosen as a representative. Variation in signal was most noticeable with the 40-ng application, which was analyzed quantitatively using densitometry (Fig 7). When the background was subtracted from the densitometry reading, three categories of antibody recognition signal intensities were apparent in comparison to the peptide representing the H2-H3 loop from *A*. *hypogaea*: 1) 50% or less was considered to possess low antibody binding affinity, 2) 51–75% was considered intermediate, and 3) 76% and above was regarded to have high affinity.

As expected, crude protein extract (CPE) from *A*. *hypogaea*, the purified *Ara h 2* protein, and the synthetic polypeptide representing part of the loop containing the DPYSPS motif from *A*. *hypogaea* homologue were highly recognized (Fig 7). On the other extreme, *A*. *triseminata* displayed the lowest IgE-binding ability, 30% of the *A*. *hypogaea* signal. This could be attributed in part to its lack of the DPYSPS-containing epitopes. The reduction in IgE-binding ability (Fig 7) in *A*. *guaranitica* (50.6% of the isolated *Ara h 2* protein and 69.4% of the *A*. *triseminata*) can be attributed to the presence of only one epitope, 7b (Fig 2). Although both *A*. *glandulifera* and *A*. *batizocoi* possess two DPYSPS-containing epitopes, the former possesses epitope 7 and 7b whereas the latter has epitopes 6 and 7b with an S mutated to T. The difference in reactivity (Fig 7) may imply that these epitopes have different magnitudes of allergenicity that are further influenced by residue substitutions. The immunoblot findings, which focused on the immunodominant epitopes, are in line with the [17] observation that the presence of more than one motif of DPYSPS in *Ara h 2* could present allergenic risk. Further, these findings demonstrate that it is not a mere additive effect that influences allergenicity, but also the differential impact of newly added epitope. Our homology modeling of these epitopes revealed different lengths of predicted disordered structure for them that further supports such variation in immunoreactivity, as will be described below.

### Epitope prediction and homology modeling

**Fig 8:**
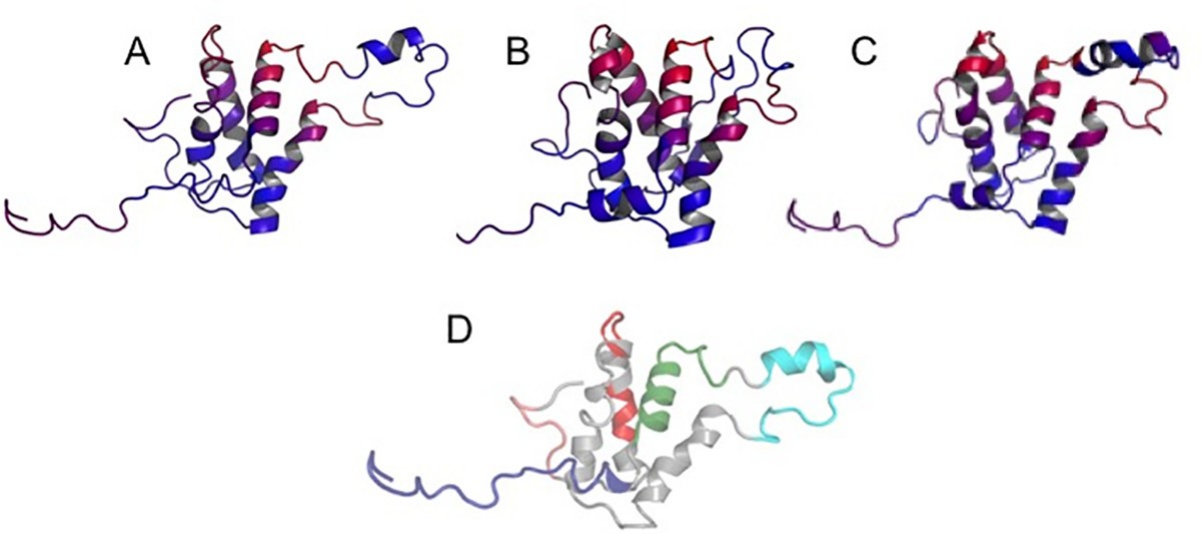
Homology models of allergenic homologues colored based on DiscoTope prediction. (A) A. hypogaea 2.01, (B) A. hypogaea 2.02, and (C) A. triseminata. Predictions are illustrated as a heatmap on the protein surface: Red  =  high prediction score and Blue  =  low prediction score. The region representing epitopes 6, 7, and 7b is highlighted. Arachis hypogaea has more red designation predicted by DiscoTope in this region and less helical structure, indicating more potential for binding IgE. (D) is homology model of A. hypogaea 2.01 with epitopes mapped: epitopes 1–3 (dark blue), epitope 4–5 (green), epitope 6–7 (cyan), epitope 8–9 (red) and epitope 10 (pink). Structure is rendered in the same orientation as A and B.

A variety of sequence and structure prediction tools were applied to the *Ara h 2 and Ara h 6* homologues in this study to further connect sequence to structure to epitope presence. These tools provided information on disordered regions, IgE binding regions, and secondary structure, allowing us to assess sequence identity/similarity and the location of sequence motifs of known epitopes (Fig 8 and S3 Fig). This sequence and structural information was considered in light of the experimental reports of the epitope positions on these proteins, particularly the immunodominant epitopes 6 and 7 [14, 29]. Analysis of the sequence-based structure using PSIPRED predictions for all species included here showed the DPYSPS sequence motif in epitope regions 6 and 7 has different lengths of predicted disordered structure that further supports the variation in immunoreactivity observed experimentally in the dot blots. This conclusion is based on the structurally disordered epitopes being more efficient at binding antigens [73] and that PSIPRED predicts both disordered protein regions and disordered protein binding regions.

Our sequence-based epitope prediction demonstrates that the absence of DPYSPS epitopes results in fewer residues having disordered or protein binding structure whereas the presence of one DPYSPS motif (e.g. epitope 6) increases the number of predicted residues involved in protein binding or disordered structure. It was also observed that the start of epitope 6 at position 65 and the presence of multiple DPYSPS motifs (species containing all epitope 6, 7, and 7b) had the largest number of residues involved in disordered structure and protein binding regions, suggesting high allergenicity. A lack of epitope 6 in *A*. *trinitensis*, *A*. *glandulifera*, *A*. *praecox*, *A*. *palustris* was predicted to result in moderately allergenic protein given the length of predicted disordered and protein binding residues. Lastly, species in Group Erectoides and Section Trisminiatae (*A*. *macedoi*, *A*. *guaranitica*, *A*. *appressipila*, *A*. *paraguariensis*, *A*. *dardani*, *A*. *tuberosa*, and *A*. *triseminata*) lack the DPYSPS motif but contain a DPY(G or T)PS motif. Their sequence-based structure and epitope prediction suggest that they are less allergenic than the other species. Taken together, results of sequence-based epitope prediction showed these regions to be epitope and/or disordered protein regions that impact allergenicity (Fig 8 and S3 Fig).

Our three-dimensional models of the *Ara h 2* structure demonstrate that the dominant epitopes are located on surface loops (Fig 8), and consequently are more accessible for IgE binding. Models were generated and validated using the NMR structure of *Ara h 6* from *A*. *hypogaea* as a template (PDB ID: 1W2Q, [37], the rationale for which was presented in Materials and Methods. Our Ara h 2 homology models revealed an overlap with *Ara h 6*, showing structural similarity in the core and in an extended loop region in a position similar to the Ara h 2 loop region containing epitopes 6 and 7 (aligned root-mean-square deviation (RMSD) of structures was 3.4 Å). As noted earlier, an overlay of the *Ara h 2* structure from homology modeling with the MBP bound crystal structure of *Ara h 2* showed the latter structure missing a major loop region containing epitope 6 and 7, a region essential to our immunological-focused study (S3 Fig), which was a major factor in selecting the *Ara h 6* NMR structure as template. An alignment overlay of the *Ara h 2* x-ray structure from PDB ID: 3OB4 was performed with the *Ara h 2* model of *A*. *hypogaea* and had an RMSD of 3.7 Å, while an alignment overlay of the *Ara h* 2 x-ray structure (PDB ID: 3OB4) and Ara h 6 NMR structure (PDB ID: 1W2Q) had an RMSD of 2.3 Å. Collectively, all structures (model, templates) have conserved helical domains, indicating the representation of major *Ara h 2* template structure components in the model, while allowing for additional modeling of the loop region containing epitopes 6 and 7 from the *Ara h 6* template structure selection.

Epitopes on antigens are distinguished from the remainder of the surface in properties such as amino acid composition, secondary structural composition, and evolutionary conservation [74]. With regard to amino acid composition, epitopes are enriched in tyrosine and tryptophan, and have a general preference for charged and polar amino acids. The sequence DPYSPS contains residues meeting those properties. Rubinstein et al. (2008) [74] noted that epitopes are not enriched in proline residues. However, proline residues contribute to disordered structure, which is common in epitopes. Bernard et al (2015) [17] observed that IgE binding to *Ara h 2* was enhanced by *Ara h 2* peptides containing three proline-contacting DPYSPS motifs compared to *Ara h 2* peptides containing only two motifs.

Sequence and structural data for *Ara h 6* relative to antigenicity are less clear, one reason being the paucity of data related to immunoreactivity of the *Ara h 6* homologue. Sequence based epitope analysis of *Ara h 6* suggests several linear epitopes within the structure [29, 75], with some of these being homologous to epitopes in *Ara h 2*. The relationship of these epitopes to structural features is less clear. For example, a large loop extending from residues 55–71 in the three-dimensional structure of *Ara h 6* is predicted to be disordered, containing the linear epitope YDSYDIR, but this epitope does not appear to be strongly immunoreactive based on our sequence-based epitope prediction. Regions from residues 80–90 and 94–106 also are reported to be immunoreactive, but structural prediction data indicate a low probability for disorder, and the NMR three-dimensional structure reveals these regions to contain coil structure interrupted by helices (S3 Fig). The C-terminal region of *Ara h 6* is predicted to be disordered as observed in the three-dimensional structure, which is consistent with the immunoreactivity of this region [45]. While lacking the DPYSPS motif, *Ara h 6* is still among the most allergenic epitopes in peanuts [1,17] despite a lack of difference in disordered protein regions across the species as compared to *Ara h 2*.

## Conclusions

This study is the first in-depth exploration of *Arachis* wild species for naturally occurring genetic alterations in genes encoding allergen proteins. It has been demonstrated that experimentally induced targeted mutations in peanuts reduce allergenicity significantly [14, 15, 31]. Focusing on the highly allergenic *Ara h 2 and Ara h 6*, we uncovered a wealth of point mutations and indels. Importantly, the mutations in Ara h 2 are concentrated mostly in the immunodominant epitope region. Our immunoblot experiments and protein structure prediction demonstrated that mutations appear to be associated with substantial reductions in allergenicity in *Arachis* species. Consequently, these genetic mutations not only provide valuable insight into interaction between mutation type/positioning, protein structure and allergenicity, but also represent cost-effective raw material for experimental work aiming at reduction/elimination of allergenicity and possibly usage in human therapy treatments.

What particularly stood out is that molecular alterations to the allergens ORFs was substantially skewed towards *Ara h 2*, pointing to a contrasting mode of gene evolution that differentially impacts the allergenic proteins. Considering that protein products from both *Ara h 2* and *Ara h 6* are immunodominant, their contrasting modes of substitutions and gain/loss of amino acids raise questions concerning the driving forces directing their distinct evolutionary pathways across the genus history. We found different modes of natural selection operating on the two homologues, with *Ara h 2* being under positive diversifying selection while *Ara h 6* is under purifying selection. One hypothesis is that *Ara h 6* was already highly allergenic at the time of the genus divergence from its common ancestors and, thus, one can argue that purifying selection has been operating to maintain the status quo, possibly due to selective advantages for allergenicity. In contrast, *Ara h 2* protein at that evolutionary stage displays a lower magnitude of allergenicity and positive selection pressure favors mutations that amplify this trait. The results of our immunoblot experiment provide support for the *Ara h 2* case since early emerging species *A*. *trisemenata* displayed the lowest IgE-binding ability, 30% of the signal value in the crop *A*. *hypogaea*, a terminal species in the phylogenetic tree that accumulated three DPYSPS-containing immunodominant epitopes. Further support also is found in our structural analyses. The three-dimensional structures from homology modeling show the immunodominant epitopes to reside on surface loops, with the size of the loops being related to the degree of allergenicity. Additionally, sequence predictions indicated varying lengths of disordered and protein-binding regions in the sequence range that contains the DPYSPS motif, with species lacking the epitope having fewer residues predicted to be disordered or protein binding. The allergenicity trait has been found to be advantageous as a defense mechanism against insects and pathogens [70, 71]. However, field and laboratory experiments are needed to assess the adaptive advantages of varied magnitudes of allergenicity in *Arachis* species in their natural habitats.

This work provides insight into the complexity of the evolutionary patterns of allergy-inducing proteins in peanut and promotes similar assessments of the other peanut allergen-inducing homologues. *Ara h 2* and *Ara h 6* present two distinctive platforms for improving our understanding of peanut allergenicity and its manipulation at the molecular level. The study strongly encourages exploration of the genetic pool for insights into the molecular basis of allergenicity in other allergy-inducing organisms.

## Supporting information

S1 Table *Arachis* species used and their respective sectional classification along with information on material sources, open reading frame length in nucleotides, and GenBank accession numbers. Italicized GenBank numbers represent accessions sequenced in this study.
(PDF)

S2 Table Amino acid frequencies as percentages of the total for *Ara h 2* and *Ara h 6* in the *Arachis* species studied. (PDF)

S3 Table Output from the FUBAR analyses of the *Ara h 2* and *Ara h 6* amino acid data sets. Residues under positive/diversifying selection are highlighted in green while those under negative/purifying selectin are highlighted in gray.
(PDF)

S1 Fig Amino acid alignments of *Ara h2* and *Ara h 6* for the *Arachis* species examined in this study deduced from the nucleotide sequence alignment. Epitopes are mapped on the top of the alignments. *Ara h 2* alignment is shown first followed by *Ara h 6* after the stop codon of *Ara h 2*. The notations gb, gb 2 and gen refer to GenBank sequence accessions. The *Ara h 2* sequences of *A*. *monticola*, *A*. *helodes* and *A*. *trintensis* are missing the upstream 5’ region 33 amino acid residues and the first codon position of the 34^th^ residue and consequently the latter codon was noted in the translation by the program as “&P&C”. The *Ara h 6* sequences of *A*. *ipaensis* and *A*. *duranensis* are missing the first 17 and 14 amino acid residues, respectively, and the symbols in the next downstream residue denote the absence of their first codon positions.
(PDF)

S2 Fig Amino acid frequencies as percentages of the total deduced amino acid of *Ara h 2* and *Ara h 6* in the *Arachis* species examined here. The amino acid symbols refer to: A: Alanine, C: Cysteine, D: Aspartic acid, E: Glutamic acid, F: Phenylalanine, G: Glycine, H: Histidine, I: Isoleucine, K: Lysine, L: Leucine, M: Methionine, N: Asparagine, P: Proline, Q: Glutamine, R: Arginine, S: Serine, T: Threonine, V: Valine, W: Tryptophan, Y: Tyrosine.
(PDF)

S3 Fig Protein structure overlay and sequence-based epitope and disordered structure predictions for all *Arachis* sequences in this work. Detailed descriptions of structures are noted.
(PDF)
