# Supplementary material for: Molecular evolution of genes encoding allergen proteins in the peanuts genus Arachis: Structural and functional implications
Source: PLoS One. 2019 Nov 1;14(11):e0222440. doi: 10.1371/journal.pone.0222440 (PMC6824556; doi:10.1371/journal.pone.0222440)
Supplement: S1 Table — Italicized GenBank numbers represent accessions sequenced in this study. (PDF) [file pone.0222440.s001.pdf]

reading frame length in nucleotides, and GenBank accession numbers. GenBank numbers for accessions generated in this study start with “MN”.

Taxa	Species	PI[a]	ORF[b] base-pair Length		GenBank accession number	
Arachis			Ara h 2	Ara h 6	Ara h 2	*Ara h 6*
Sec. *Arachis*	*A. batizocoi* Krapov. & W.C. Gregory	298639	498	438	MN027227	MN027261
	A. duranensis Krapov. & W.C. Gregory	219823	483	438	MN027223	MN027266
	A. duranensis gb mRNA		483	438	EF609641.1	EF609638.1
	A. glandulifera Stalker	468336	513	438	MN027225	MN027253
	A. helodes Martius ex Krapov. & Rigoni	468144	510	438	MN027243	MN027263
	A. hoehnei Krapov. & W.C. Gregory	468150	504	438	MN027226	MN027258
	A. hypogaea L.	262090	483	438	MN027224	MN027245
	A. hypogaea 2.01		477	NA	FJ713110.1	--
	A. hypogaea 2.02		519	NA	AY158467.1	--
	A. ipaensis Krapov. & W.C. Gregory	468322	519	438	MN027222	MN027265
	A. ipaensis gb conglutin transcript variant		483	NA	XM01631498	--
	A. ipaensis gb2 conglutin transcript variant		519	NA	XM01631498	--
	A. ipaensis mRNA		NA	438	--	EF6099640.1
	A. monticola Krapov. & Rigoni	BaRik 7264[c]	525	438	MN027242	MN027246
	A. palustris Krapov., W.C. Gregory, & Valls	VPmSv 13023	531	438	MN027229	MN027255

	A. praecox Krapov., W.C. Gregory, & Valls	476128	537	438	MN027228	MN027254
	A. trinitensis Krapov. & W.C. Gregory	604858	477	438	MN027244	MN027264
	A. paraguariensis Chodat & Hassl.	262842	468	435	MN027237	MN027251
	A. lutescens Krapov. & Rigoni	30038	498	437	MN027240	MN027262
	A. macedoi Krapov. & W.C. Gregory	276203	471	435	MN027241	MN027260
	A. villosulicarpa Hoehne	273482	NA	438	this study	--
Sec. Caulorrhizae	*A. pintoi* Krapov. &W.C. Gregory	PI18748	477	438	MN027238	MN027256
Sec. *Heteranthae*	*A. dardani Krapov. & W.C. Gregory*	GK 12943	468	441	MN027236	MN027252
Sec. *Procumbentes*	*A. appressipilia* Krapov. & W.C. Gregory	261877	468	441	MN027232	MN027250
	A. kretschmeri Krapov. & W.C. Gregory	30007	504	438	MN027230	MN027259
	A. rigonii Krapov. & W.C. Gregory	262142	468	441	MN027235	MN027249
Sec. *Rhizomatosae*	*A. glabrata Benth.*	10596	498	438	MN027231	MN027257
Sec. *Trierectoides*	*A. guaranitica* Chodat & Hassl.	276194	483	441	MN027234	MN027248
	A. tuberosa Bong. ex Benth.	476142	543	NA	MN027233	this study

Sec. Triseminatae	*A. triseminata* Krapov. & W.C. Gregory	GK 12922	477	435	MN027239	MN027247

[a]Plant Introduction accession numbers, Germplasm Resources Information Network. [b]ORF: Open reading frame. [c]Collector number
