# Supplementary material for: Molecular evolution of genes encoding allergen proteins in the peanuts genus Arachis: Structural and functional implications
Source: PLoS One. 2019 Nov 1;14(11):e0222440. doi: 10.1371/journal.pone.0222440 (PMC6824556; doi:10.1371/journal.pone.0222440)
Supplement: S2 Table — (PDF) [file pone.0222440.s002.pdf]

S2 Table. Amino acid frequencies as percentages of the total for Ara h 2 and Ara h 6 in the *Arachis* specie

Ara h2

	Alanine	Cysteine	Aspartate	Glutamine	Phenylalanine	Glycine	Histidine	Isoleucine	Lysine	Leucine
A. ipaensis	6.4	4.7	7.0	8.1	1.7	4.7	1.7	1.7	1.7	9.9
A. ipaensis gen	6.9	5.0	5.6	8.8	1.9	5.0	1.9	1.9	1.9	10.6
A. ipaensis gen.	6.4	4.7	7.0	8.1	1.7	4.7	1.7	1.7	1.7	9.9
A. duranensis	6.9	5.0	6.3	8.8	1.9	4.4	1.9	1.9	1.9	10.6
A. duranensis g	6.9	5.0	6.3	8.8	1.9	4.4	1.9	1.9	1.9	10.6
A. hypogaea	6.9	5.0	6.3	8.8	1.9	4.4	1.9	1.9	1.9	10.6
A. monticola	2.9	5.8	6.6	10.2	1.5	4.4	1.5	1.5	1.5	7.3
A. triseminata	7.0	5.1	4.4	9.5	1.9	3.8	2.5	2.5	1.9	10.8
A. tuberosa	6.1	4.4	6.1	7.2	1.7	6.1	1.7	1.7	1.7	9.4
A. guaranitica	6.9	5.0	6.9	8.1	1.9	4.4	1.9	1.9	1.9	10.6
A. rigonii	7.1	5.2	6.5	8.4	1.9	3.9	1.9	1.9	1.9	11.0
A. appressipila	7.1	5.2	6.5	8.4	1.9	4.5	1.9	1.9	1.9	11.0
A. paraguarien.	6.5	5.2	6.5	8.4	2.6	4.5	1.9	1.9	1.9	10.3
A. dardani	6.5	5.2	6.5	8.4	1.9	4.5	1.9	1.9	1.9	11.0
A. glandulifera	5.9	4.7	8.2	7.6	1.8	4.7	2.4	1.8	1.2	10.0
A. praecox	6.2	4.5	7.3	8.4	1.7	4.5	2.2	2.2	1.7	9.6
A. palustris	6.8	4.5	7.4	8.5	1.7	4.5	1.7	1.7	1.7	9.7
A. pintoi	5.7	5.1	5.1	8.9	1.9	4.4	2.5	1.9	1.9	10.8
A. glabrata	6.1	4.8	6.7	8.5	1.8	4.2	2.4	1.8	2.4	10.3
A. hoehnei	6.6	4.8	6.6	8.4	1.8	4.2	1.8	1.8	1.8	10.2
A. kretschmeri	6.6	4.8	6.6	8.4	1.8	4.2	1.8	1.8	1.8	10.2
A. macedoi	5.8	5.1	7.1	7.7	1.9	3.8	2.6	2.6	1.9	10.9
A. batizocoi	6.1	4.8	6.7	8.5	1.8	4.2	2.4	1.8	2.4	10.3
A. lutescens	6.7	4.2	6.7	8.5	1.8	4.2	2.4	1.8	1.8	10.3
A. villosulicarp	6.9	5.0	6.3	8.8	1.9	4.4	1.9	1.9	1.9	10.6
A. helodes	3.0	6.0	7.5	9.0	1.5	4.5	1.5	1.5	1.5	7.5
A. trinitensis	2.9	5.8	8.7	10.1	2.2	4.3	1.4	1.4	1.4	7.2
Avg.	6.2	5.0	6.6	8.5	1.8	4.4	2.0	1.9	1.8	10.1

Ara h6

	Alanine	Cysteine	Aspartate	Glutamine	Phenylalanin	Glycine	Histidin	Isoleucine	Lysine	Leucine
A. ipaensis	3.1	7.9	7.9	8.7	1.6	3.9	0.8	3.1	1.6	5.5
A. ipaensis gen.	5.5	6.9	6.9	7.6	1.4	3.4	1.4	3.4	2.1	8.3
A. duranensis	3.8	7.7	8.5	8.5	1.5	3.8	1.5	3.1	1.5	5.4
A. duranensis g	5.5	6.9	7.6	7.6	1.4	3.4	1.4	3.4	2.1	8.3
A. hypogaea	5.5	6.9	6.9	7.6	1.4	3.4	1.4	3.4	2.1	8.3
A. monticola	4.8	6.9	6.9	7.6	2.1	3.4	0.7	3.4	2.1	9.0
A. triseminata	5.6	6.9	7.6	5.6	1.4	4.9	3.5	4.2	2.1	8.3
A. guaranitica	5.5	6.8	6.8	6.2	1.4	3.4	1.4	3.4	1.4	8.9
A. rigonii	5.5	6.8	7.5	6.2	1.4	3.4	1.4	3.4	2.1	8.2
A. appressipila	5.5	6.8	7.5	6.2	1.4	3.4	1.4	3.4	2.1	8.2

A. paraguarien.	5.6	6.9	7.6	5.6	1.4	4.9	3.5	4.2	2.1	8.3
A. dardani	5.5	6.8	7.5	6.2	1.4	3.4	1.4	3.4	2.1	8.2
A. glandulifera	6.2	6.9	6.9	7.6	1.4	3.4	1.4	3.4	2.1	8.3
A. praecox	6.2	6.9	7.6	6.9	1.4	3.4	1.4	3.4	2.1	8.3
A. palustris	5.5	6.9	7.6	6.9	1.4	3.4	1.4	3.4	2.1	8.3
A. pintoi	5.5	6.9	7.6	7.6	1.4	3.4	1.4	3.4	2.1	8.3
A. glabrata	4.8	6.9	8.3	5.5	1.4	2.8	2.1	3.4	2.1	8.3
A. hoehnei	4.8	6.9	7.6	7.6	1.4	3.4	1.4	4.1	2.1	8.3
A. kretschmeri	5.5	6.9	7.6	7.6	1.4	3.4	1.4	4.1	2.1	8.3
A. macedoi	4.9	6.9	9.0	4.2	1.4	4.2	2.8	3.5	2.1	8.3
A. batizocoi	5.5	6.9	7.6	6.9	1.4	3.4	1.4	3.4	2.1	8.3
A. lutescens	5.5	6.9	7.6	7.6	1.4	3.4	1.4	4.1	2.1	7.6
A. villosulicarpo	5.5	6.9	6.9	7.6	1.4	3.4	1.4	3.4	2.1	8.3
A. helodes	4.8	6.9	6.9	7.6	1.4	3.4	1.4	3.4	2.1	8.3
A. trinitensis	6.2	6.9	6.9	7.6	1.4	3.4	2.1	3.4	2.1	8.3
Avg.	5.3	7.0	7.5	7.0	1.4	3.6	1.6	3.6	2.0	8.1

:s studied.

MethionineAsparagineProlineGlutamineArginine					SerineThreonineValineTryptophanTyrosine				
Methionine	Asparagin	Proline	Glutamine	Arginin	Serine	Threonine	Valine	Tryptophar	Tyrosine
2.3	4.1	6.4	14.0	12.2	7.6	0.6	1.2	0.6	3.5
2.5	4.4	5.0	14.4	11.9	6.9	0.6	1.3	0.6	3.1
2.3	4.1	6.4	14.0	12.2	7.6	0.6	1.2	0.6	3.5
2.5	4.4	5.0	14.4	11.9	6.9	0.6	1.3	0.6	3.1
2.5	4.4	5.0	14.4	11.9	6.9	0.6	1.3	0.6	3.1
2.5	4.4	5.0	14.4	11.9	6.9	0.6	1.3	0.6	3.1
2.2	5.1	7.3	16.1	11.7	9.5	0.0	0.7	0.0	4.4
2.5	4.4	3.8	16.5	12.7	5.7	0.6	0.6	0.6	3.2
2.2	3.3	9.4	13.9	10.6	7.2	0.6	1.1	0.6	5.0
2.5	3.8	5.6	15.6	11.9	5.6	0.6	1.3	0.6	3.1
2.6	3.9	5.2	15.5	11.6	6.5	0.6	1.3	0.6	2.6
2.6	3.9	5.2	15.5	11.6	5.8	0.6	1.3	0.6	2.6
2.6	3.9	5.2	15.5	11.6	6.5	0.6	1.3	0.6	2.6
2.6	3.9	5.2	15.5	11.6	6.5	0.6	1.3	0.6	2.6
2.4	4.7	5.9	14.1	12.4	7.1	0.6	1.2	0.6	2.9
2.2	3.9	7.9	12.4	12.4	7.3	0.6	1.1	0.6	3.4
2.3	4.0	6.8	13.1	12.5	7.4	0.6	1.1	0.6	3.4
2.5	4.4	5.1	15.2	12.0	7.0	0.6	1.3	0.6	3.2
2.4	4.2	5.5	13.9	11.5	6.7	1.2	1.2	0.6	3.6
3.0	4.2	6.0	14.4	12.0	6.0	1.2	1.2	0.6	3.6
3.0	4.2	6.0	14.4	12.0	6.6	0.6	1.2	0.6	3.6
2.6	4.5	3.2	17.3	12.2	5.1	1.3	0.6	0.6	3.2
2.4	4.2	5.5	13.9	11.5	6.7	1.2	1.2	0.6	3.6
2.4	4.2	5.5	13.9	12.7	6.7	0.6	1.2	0.6	3.6
2.5	4.4	5.0	14.4	11.9	6.9	0.6	1.3	0.6	3.1
2.2	5.2	7.5	15.7	11.9	9.0	0.0	0.7	0.0	4.5
2.2	5.1	7.2	15.2	13.8	7.2	0.0	0.7	0.0	2.9
2.5	4.2	5.8	14.7	12.0	6.8	0.6	1.1	0.6	3.3

Methionin	Asparagin	Prolin	Glutamin	Arginin	Serine	Threonin	Valine	Tryptopha	Tyrosine
6.3	4.7	2.4	15.7	13.4	7.1	1.6	3.1	0.0	1.6
6.2	4.1	2.1	13.8	11.7	6.9	2.1	4.8	0.0	1.4
6.2	3.8	2.3	15.4	13.1	6.9	1.5	3.8	0.0	1.5
6.2	3.4	2.1	13.8	11.7	6.9	2.1	4.8	0.0	1.4
6.2	4.1	2.1	13.8	11.7	6.9	2.1	4.8	0.0	1.4
6.2	4.1	2.1	13.8	11.7	6.9	2.1	5.5	0.0	0.7
6.3	4.2	2.1	12.5	9.7	6.9	1.4	4.2	0.7	2.1
6.2	4.1	2.1	15.1	11.0	6.8	2.1	4.8	0.7	2.1
6.2	4.1	2.1	14.4	11.6	6.8	2.1	4.8	0.7	1.4
6.2	4.1	2.1	14.4	11.6	6.8	2.1	4.8	0.7	1.4

6.3	4.2	2.1	12.5	9.7	6.9	1.4	4.2	0.7	2.1
6.2	4.1	2.1	14.4	11.6	6.8	2.1	4.8	0.7	1.4
6.2	4.1	2.1	13.8	11.7	6.2	2.1	4.8	0.0	1.4
6.2	3.4	2.1	14.5	11.7	6.2	2.1	4.8	0.0	1.4
6.2	3.4	2.1	14.5	11.7	6.9	2.1	4.8	0.0	1.4
6.2	3.4	2.1	13.8	11.7	6.9	2.1	4.8	0.0	1.4
6.2	3.4	2.1	14.5	10.3	7.6	2.8	4.8	0.7	2.1
6.2	3.4	2.1	13.8	11.7	6.9	2.8	4.1	0.0	1.4
6.9	3.4	2.1	13.8	11.7	6.2	2.1	4.1	0.0	1.4
5.6	4.2	2.1	13.9	9.7	6.9	2.1	4.9	0.7	2.8
6.2	3.4	2.1	14.5	11.7	6.9	2.1	4.8	0.0	1.4
6.9	3.4	2.1	13.8	11.7	6.9	2.1	4.1	0.0	1.4
6.2	4.1	2.1	13.8	11.7	6.9	2.1	4.8	0.0	1.4
6.2	4.1	2.1	13.8	11.7	6.9	2.1	5.5	0.0	1.4
6.2	4.1	2.1	13.8	11.0	6.2	2.1	4.8	0.0	1.4
6.2	3.9	2.1	14.1	11.5	6.8	2.0	4.6	0.2	1.5
