# Supplementary material for: Molecular evolution of genes encoding allergen proteins in the peanuts genus Arachis: Structural and functional implications
Source: PLoS One. 2019 Nov 1;14(11):e0222440. doi: 10.1371/journal.pone.0222440 (PMC6824556; doi:10.1371/journal.pone.0222440)
Supplement: S3 Table — Residues under positive/diversifying selection are highlighted in green while those under negative/purifying selectin are highlighted in gray. (PDF) [file pone.0222440.s003.pdf]

Ara h 2

Site	Partition	α	β	β-α	Prob[&alpha	Prob[&alpha
1	1	4.035	0.47	-3.565	0.651	0.297
2	1	2.625	1.368	-1.257	0.541	0.404
3	1	4.748	11.165	6.417	0.229	0.717
4	1	3.195	12.17	8.975	0.04	0.862
5	1	12.018	1.366	-10.652	0.791	0.165
6	1	0.769	1.114	0.345	0.317	0.625
7	1	0.766	0.513	-0.253	0.54	0.397
8	1	1.384	0.481	-0.903	0.593	0.348
9	1	0.624	0.56	-0.064	0.503	0.432
10	1	0.795	0.507	-0.288	0.546	0.391
11	1	0.766	0.498	-0.268	0.545	0.392
12	1	0.765	0.495	-0.271	0.547	0.39
13	1	0.766	0.498	-0.268	0.545	0.392
14	1	0.651	0.495	-0.156	0.529	0.406
15	1	1.204	0.932	-0.273	0.375	0.566
16	1	0.765	0.495	-0.271	0.547	0.39
17	1	3.06	0.495	-2.565	0.815	0.147
18	1	2.32	0.497	-1.823	0.792	0.167
19	1	0.766	0.498	-0.268	0.545	0.392
20	1	1.246	0.485	-0.761	0.583	0.358
21	1	0.795	0.498	-0.296	0.549	0.388
22	1	2.66	0.476	-2.184	0.81	0.151
23	1	0.826	0.498	-0.328	0.552	0.385
24	1	0.982	0.501	-0.481	0.565	0.374
25	1	1.613	8.507	6.895	0.048	0.913
26	1	1.391	0.547	-0.844	0.571	0.371
27	1	4.048	0.499	-3.549	0.642	0.307
28	1	1.015	0.538	-0.477	0.556	0.384
29	1	5.89	0.548	-5.342	0.929	0.05
30	1	0.997	1.714	0.717	0.298	0.646
31	1	0.795	0.529	-0.266	0.539	0.398
32	1	1.247	0.496	-0.751	0.578	0.362
33	1	0.807	0.513	-0.293	0.546	0.392
34	1	0.807	0.513	-0.293	0.546	0.392
35	1	1.177	0.455	-0.722	0.593	0.347
36	1	1.324	0.535	-0.788	0.571	0.37
37	1	1.178	0.473	-0.705	0.585	0.356
38	1	1.324	0.535	-0.788	0.571	0.37
39	1	2.58	0.485	-2.095	0.805	0.156
40	1	1.323	0.512	-0.81	0.578	0.363
41	1	0.946	0.491	-0.454	0.566	0.373

42	1	3.358	0.49	-2.868	0.823	0.141
43	1	1.179	0.5	-0.678	0.574	0.366
44	1	10.699	0.56	-10.14	0.987	0.006
45	1	0.946	0.491	-0.454	0.566	0.373
46	1	2.852	0.478	-2.374	0.814	0.149
47	1	1.177	0.455	-0.722	0.593	0.347
48	1	1.323	0.512	-0.81	0.578	0.363
49	1	5.359	0.561	-4.798	0.844	0.124
50	1	6.111	0.482	-5.629	0.869	0.104
51	1	0.742	0.486	-0.257	0.547	0.389
52	1	4.03	0.461	-3.569	0.655	0.294
53	1	1.324	0.535	-0.788	0.571	0.37
54	1	9.711	0.53	-9.181	0.891	0.086
55	1	0.935	0.486	-0.449	0.566	0.373
56	1	8.344	0.561	-7.783	0.879	0.096
57	1	0.635	0.483	-0.152	0.531	0.404
58	1	1.303	1.072	-0.231	0.363	0.579
59	1	1.357	1.301	-0.056	0.347	0.596
60	1	0.78	2.75	1.97	0.152	0.801
61	1	3.909	3.055	-0.854	0.525	0.423
62	1	5.499	11.976	6.478	0.111	0.74
63	1	0.842	0.522	-0.32	0.546	0.392
64	1	0.984	9.853	8.869	0.021	0.955
65	1	8.13	0.736	-7.394	0.858	0.112
66	1	2.023	0.686	-1.337	0.566	0.377
67	1	1.126	0.562	-0.564	0.554	0.386
68	1	2.066	8.688	6.622	0.107	0.851
69	1	3.161	1.139	-2.022	0.56	0.386
70	1	1.881	1.176	-0.705	0.531	0.413
71	1	2.698	12.331	9.633	0.082	0.873
72	1	2.737	5.831	3.094	0.251	0.698
73	1	1.544	0.759	-0.785	0.545	0.397
74	1	1.36	0.668	-0.692	0.549	0.393
75	1	2.024	0.717	-1.307	0.563	0.381
76	1	2.021	0.658	-1.364	0.57	0.374
77	1	0.994	0.667	-0.327	0.53	0.41
78	1	1.007	0.528	-0.479	0.555	0.384
79	1	1.496	0.567	-0.929	0.57	0.372
80	1	7.52	0.531	-6.989	0.868	0.105
81	1	0.898	0.51	-0.388	0.555	0.384
82	1	1.457	8.9	7.443	0.042	0.923
83	1	0.933	4.334	3.401	0.119	0.839
84	1	3.65	6.984	3.335	0.174	0.771

85	1	3.504	1.342	-2.162	0.592	0.35
86	1	0.799	0.478	-0.321	0.558	0.38
87	1	1.179	0.501	-0.678	0.574	0.366
88	1	10.274	15.855	5.581	0.213	0.634
89	1	0.74	0.536	-0.204	0.531	0.406
90	1	5.306	0.538	-4.768	0.848	0.121
91	1	4.603	0.539	-4.064	0.838	0.129
92	1	14.192	0.539	-13.653	0.98	0.014
93	1	4.263	3.626	-0.638	0.52	0.428
94	1	4.263	3.626	-0.638	0.52	0.428
95	1	4.263	3.626	-0.638	0.52	0.428
96	1	4.263	3.626	-0.638	0.52	0.428
97	1	4.263	3.626	-0.638	0.52	0.428
98	1	4.263	3.626	-0.638	0.52	0.428
99	1	4.263	3.626	-0.638	0.52	0.428
100	1	4.263	3.626	-0.638	0.52	0.428
101	1	4.263	3.626	-0.638	0.52	0.428
102	1	4.263	3.626	-0.638	0.52	0.428
103	1	4.263	3.626	-0.638	0.52	0.428
104	1	4.263	3.626	-0.638	0.52	0.428
105	1	4.263	3.626	-0.638	0.52	0.428
106	1	4.263	3.626	-0.638	0.52	0.428
107	1	4.263	3.626	-0.638	0.52	0.428
108	1	4.263	3.626	-0.638	0.52	0.428
109	1	4.263	3.626	-0.638	0.52	0.428
110	1	4.263	3.626	-0.638	0.52	0.428
111	1	4.263	3.626	-0.638	0.52	0.428
112	1	4.263	3.626	-0.638	0.52	0.428
113	1	12.973	0.754	-12.219	0.894	0.083
114	1	1.008	0.667	-0.342	0.53	0.409
115	1	13.472	0.699	-12.772	0.898	0.08
116	1	12.86	0.703	-12.157	0.894	0.083
117	1	4.838	10.709	5.871	0.245	0.701
118	1	2.257	1.284	-0.973	0.538	0.407
119	1	17.753	2.488	-15.265	0.805	0.145
120	1	4.358	1.265	-3.094	0.658	0.28
121	1	3.32	4.444	1.123	0.319	0.572
122	1	4.527	0.508	-4.019	0.844	0.124
123	1	0.772	6.052	5.28	0.037	0.935
124	1	0.632	1.119	0.487	0.293	0.65
125	1	0.771	0.519	-0.252	0.54	0.398
126	1	0.743	0.468	-0.274	0.555	0.382
127	1	10.67	0.484	-10.186	0.992	0.004

128	1	1.324	0.535	-0.788	0.571	0.37
129	1	1.178	0.476	-0.702	0.584	0.357
130	1	6.495	0.566	-5.929	0.86	0.112
131	1	1.384	3.7	2.316	0.161	0.789
132	1	0.946	0.491	-0.454	0.566	0.373
133	1	0.786	0.45	-0.335	0.569	0.368
134	1	1.177	0.455	-0.722	0.593	0.347
135	1	3.437	0.504	-2.933	0.821	0.143
136	1	1.323	0.512	-0.81	0.578	0.363
137	1	0.618	0.548	-0.07	0.505	0.431
138	1	1.179	0.5	-0.678	0.574	0.366
139	1	1.323	0.512	-0.81	0.578	0.363
140	1	3.43	0.45	-2.98	0.838	0.128
141	1	1.323	0.512	-0.81	0.578	0.363
142	1	1.179	0.5	-0.678	0.574	0.366
143	1	1.202	1.165	-0.037	0.348	0.594
144	1	0.977	0.554	-0.423	0.549	0.39
145	1	0.946	0.491	-0.454	0.566	0.373
146	1	1.189	0.905	-0.285	0.379	0.562
147	1	4.03	0.461	-3.569	0.655	0.294
148	1	1.177	0.455	-0.722	0.593	0.347
149	1	10.229	1.381	-8.847	0.767	0.185
150	1	0.771	0.489	-0.282	0.55	0.387
151	1	0.675	0.521	-0.154	0.524	0.412
152	1	0.977	0.554	-0.423	0.549	0.39
153	1	1.324	0.535	-0.788	0.571	0.37
154	1	8.42	0.486	-7.934	0.893	0.084
155	1	4.03	0.461	-3.569	0.655	0.294
156	1	1.323	0.512	-0.81	0.578	0.363
157	1	1.179	0.5	-0.678	0.574	0.366
158	1	1.324	0.535	-0.788	0.571	0.37
159	1	1.178	0.473	-0.705	0.585	0.356
160	1	0.786	0.485	-0.3	0.553	0.384
161	1	0.946	0.491	-0.454	0.566	0.373
162	1	0.675	0.521	-0.154	0.524	0.412
163	1	1.324	0.535	-0.788	0.571	0.37
164	1	3.655	0.497	-3.158	0.827	0.138
165	1	0.946	0.491	-0.454	0.566	0.373
166	1	0.977	0.554	-0.423	0.549	0.39
167	1	1.324	0.535	-0.788	0.571	0.37
168	1	1.323	0.512	-0.81	0.578	0.363
169	1	0.977	0.554	-0.423	0.549	0.39
170	1	1.324	0.535	-0.788	0.571	0.37

171	1	1.176	0.448	-0.729	0.596	0.344
172	1	1.323	0.514	-0.808	0.577	0.364
173	1	0.946	0.491	-0.454	0.566	0.373
174	1	1.323	0.512	-0.81	0.578	0.363
175	1	0.742	0.486	-0.257	0.547	0.389
176	1	0.946	0.491	-0.454	0.566	0.373
177	1	1.179	0.5	-0.678	0.574	0.366
178	1	0.675	0.521	-0.154	0.524	0.412
179	1	0.635	0.478	-0.157	0.533	0.402
180	1	0.977	0.554	-0.423	0.549	0.39
181	1	9.04	0.559	-8.481	0.883	0.093
182	1	1.177	0.455	-0.722	0.593	0.347
183	1	0.761	1.134	0.373	0.314	0.628
184	1	25.508	1.443	-24.065	0.983	0.004
185	1	0.946	0.491	-0.454	0.566	0.373
186	1	3.097	1.103	-1.993	0.634	0.301
187	1	0.771	0.478	-0.293	0.555	0.382
188	1	1.324	0.535	-0.788	0.571	0.37
189	1	0.635	0.483	-0.152	0.531	0.404
190	1	1.177	0.455	-0.722	0.593	0.347
191	1	9.36	0.5	-8.86	0.897	0.081
192	1	0.675	0.521	-0.154	0.524	0.412
193	1	1.547	8.505	6.957	0.033	0.931
194	1	0.775	1.137	0.362	0.316	0.626
195	1	0.977	0.527	-0.45	0.556	0.383
196	1	0.786	0.472	-0.314	0.559	0.379
197	1	0.743	0.495	-0.248	0.544	0.393
198	1	0.743	0.495	-0.248	0.544	0.393
199	1	0.807	0.513	-0.293	0.546	0.392
200	1	9.36	0.5	-8.86	0.897	0.081
201	1	0.807	0.513	-0.293	0.546	0.392
202	1	9.846	1.185	-8.661	0.784	0.171

Ara h 6

Site	Partition	α	β	β-α	Prob[&alpha	Prob[&alpha
1	1	4.3	0.465	-3.835	0.64	0.308
2	1	0.756	1.015	0.259	0.312	0.629
3	1	39.241	0.52	-38.721	1	0
4	1	0.753	7.816	7.063	0.012	0.969
5	1	0.758	0.503	-0.254	0.527	0.41
6	1	1.443	0.478	-0.965	0.579	0.362
7	1	0.635	1.342	0.707	0.262	0.682

8	1	0.647	1.057	0.409	0.289	0.653
9	1	9.989	1.026	-8.963	0.909	0.059
10	1	0.756	0.509	-0.247	0.524	0.413
11	1	0.645	0.51	-0.135	0.507	0.428
12	1	0.758	0.499	-0.259	0.529	0.408
13	1	0.756	0.509	-0.247	0.524	0.413
14	1	0.759	1.057	0.298	0.307	0.633
15	1	2.358	0.537	-1.821	0.771	0.185
16	1	0.788	0.507	-0.281	0.528	0.41
17	1	1.969	0.498	-1.471	0.768	0.186
18	1	1.984	0.914	-1.07	0.397	0.545
19	1	0.756	1.03	0.273	0.31	0.631
20	1	11.645	0.516	-11.129	0.967	0.023
21	1	11.815	0.504	-11.312	0.968	0.022
22	1	4.306	0.524	-3.782	0.619	0.33
23	1	1.037	0.478	-0.559	0.561	0.379
24	1	3.275	0.997	-2.278	0.641	0.294
25	1	1.571	2.348	0.777	0.213	0.733
26	1	1.688	1.011	-0.677	0.375	0.567
27	1	11.971	1.014	-10.956	0.93	0.044
28	1	2.223	4.948	2.725	0.265	0.651
29	1	3.193	3.473	0.28	0.362	0.538
30	1	0.788	0.492	-0.296	0.534	0.403
31	1	2.059	0.474	-1.585	0.597	0.346
32	1	0.646	0.549	-0.096	0.494	0.443
33	1	0.646	0.549	-0.096	0.494	0.443
34	1	2.058	0.46	-1.598	0.603	0.34
35	1	2.058	0.463	-1.595	0.602	0.341
36	1	1.54	1.003	-0.537	0.367	0.575
37	1	1.096	0.925	-0.171	0.356	0.585
38	1	1.443	0.518	-0.924	0.56	0.381
39	1	0.657	1.057	0.4	0.291	0.651
40	1	2.059	0.474	-1.585	0.597	0.346
41	1	1.037	0.478	-0.559	0.561	0.379
42	1	3.588	0.507	-3.081	0.81	0.152
43	1	2.059	0.477	-1.581	0.595	0.348
44	1	0.756	0.509	-0.247	0.524	0.413
45	1	1.442	0.505	-0.937	0.565	0.376
46	1	0.758	0.501	-0.257	0.528	0.409
47	1	2.058	0.463	-1.595	0.602	0.341
48	1	1.442	0.495	-0.948	0.569	0.372
49	1	1.443	0.518	-0.924	0.56	0.381
50	1	2.059	0.475	-1.583	0.596	0.347

51	1	1.27	0.461	-0.809	0.582	0.358
52	1	4.3	0.465	-3.835	0.64	0.308
53	1	11.872	0.523	-11.349	0.894	0.084
54	1	1.037	0.478	-0.559	0.561	0.379
55	1	12.082	0.483	-11.599	0.905	0.075
56	1	4.3	0.465	-3.835	0.64	0.308
57	1	0.758	0.492	-0.266	0.532	0.405
58	1	1.696	0.943	-0.753	0.382	0.559
59	1	0.777	1.181	0.404	0.298	0.644
60	1	1.513	2.21	0.697	0.218	0.728
61	1	1.318	1.153	-0.165	0.34	0.602
62	1	2.082	1.113	-0.969	0.376	0.567
63	1	2.059	0.474	-1.585	0.597	0.346
64	1	0.758	0.5	-0.258	0.528	0.409
65	1	2.06	0.517	-1.543	0.58	0.363
66	1	0.835	2.212	1.377	0.176	0.773
67	1	0.741	0.49	-0.251	0.531	0.406
68	1	1.037	0.478	-0.559	0.561	0.379
69	1	3.875	1.987	-1.888	0.53	0.417
70	1	0.81	0.923	0.113	0.331	0.609
71	1	0.651	1.045	0.394	0.291	0.65
72	1	0.621	1.272	0.652	0.265	0.679
73	1	0.758	0.5	-0.258	0.528	0.409
74	1	0.758	0.5	-0.258	0.528	0.409
75	1	2.059	0.474	-1.585	0.597	0.346
76	1	13.182	3.21	-9.972	0.662	0.263
77	1	4.471	0.532	-3.939	0.83	0.135
78	1	1.443	0.518	-0.924	0.56	0.381
79	1	1.037	0.478	-0.559	0.561	0.379
80	1	2.058	0.463	-1.595	0.602	0.341
81	1	2.058	0.463	-1.595	0.602	0.341
82	1	0.835	0.481	-0.354	0.546	0.392
83	1	1.442	0.495	-0.948	0.569	0.372
84	1	1.946	0.55	-1.397	0.747	0.204
85	1	16.789	0.952	-15.837	0.867	0.106
86	1	1.453	1.127	-0.326	0.349	0.593
87	1	4.3	0.465	-3.835	0.64	0.308
88	1	1.442	0.495	-0.948	0.569	0.372
89	1	2.059	0.477	-1.581	0.595	0.348
90	1	2.706	1.106	-1.6	0.6	0.331
91	1	1.453	1.099	-0.353	0.352	0.59
92	1	0.685	0.504	-0.181	0.517	0.42
93	1	2.058	0.463	-1.595	0.602	0.341

94	1	4.3	0.465	-3.835	0.64	0.308
95	1	2.058	0.463	-1.595	0.602	0.341
96	1	1.442	0.495	-0.948	0.569	0.372
97	1	0.646	0.499	-0.147	0.512	0.424
98	1	3.222	0.555	-2.667	0.79	0.168
99	1	1.443	0.518	-0.924	0.56	0.381
100	1	1.443	0.518	-0.924	0.56	0.381
101	1	1.27	0.461	-0.809	0.582	0.358
102	1	4.3	0.465	-3.835	0.64	0.308
103	1	1.442	0.495	-0.948	0.569	0.372
104	1	18.298	0.484	-17.814	0.938	0.049
105	1	1.443	0.518	-0.924	0.56	0.381
106	1	2.058	0.463	-1.595	0.602	0.341
107	1	4.22	0.484	-3.736	0.835	0.131
108	1	1.037	0.478	-0.559	0.561	0.379
109	1	0.772	0.553	-0.219	0.511	0.427
110	1	1.443	0.518	-0.924	0.56	0.381
111	1	2.059	0.474	-1.585	0.597	0.346
112	1	1.037	0.478	-0.559	0.561	0.379
113	1	0.739	0.531	-0.208	0.515	0.422
114	1	4.3	0.465	-3.835	0.64	0.308
115	1	0.788	0.507	-0.281	0.528	0.41
116	1	1.443	0.518	-0.924	0.56	0.381
117	1	1.443	0.518	-0.924	0.56	0.381
118	1	2.058	0.469	-1.589	0.599	0.344
119	1	1.453	1.063	-0.389	0.356	0.586
120	1	0.685	0.504	-0.181	0.517	0.42
121	1	1.442	0.495	-0.948	0.569	0.372
122	1	0.756	0.509	-0.247	0.524	0.413
123	1	4.3	0.465	-3.835	0.64	0.308
124	1	2.059	0.477	-1.581	0.595	0.348
125	1	0.772	0.553	-0.219	0.511	0.427
126	1	3.029	0.5	-2.529	0.802	0.158
127	1	0.739	0.531	-0.208	0.515	0.422
128	1	1.443	0.518	-0.924	0.56	0.381
129	1	0.835	0.474	-0.361	0.549	0.388
130	1	2.079	1.006	-1.073	0.387	0.555
131	1	3.98	0.476	-3.504	0.833	0.132
132	1	6.517	0.48	-6.037	0.862	0.109
133	1	0.644	1.035	0.39	0.291	0.651
134	1	0.646	0.501	-0.145	0.511	0.425
135	1	1.512	3.12	1.608	0.181	0.768
136	1	0.672	8.436	7.764	0.008	0.978

137	1	2.058	0.463	-1.595	0.602	0.341
138	1	6.257	0.482	-5.775	0.869	0.104
139	1	0.772	0.553	-0.219	0.511	0.427
140	1	9.135	0.48	-8.656	0.885	0.091
141	1	14.572	1.081	-13.491	0.937	0.039
142	1	0.835	0.467	-0.368	0.553	0.385
143	1	3.288	0.491	-2.797	0.811	0.151
144	1	0.758	0.492	-0.266	0.532	0.405
145	1	0.685	0.504	-0.181	0.517	0.42
146	1	2.058	0.463	-1.595	0.602	0.341

BayesFactor	PSRF	Neff
0.552	1.002	340.31
0.885	1.004	268.293
3.317	1.013	142.819
8.157	0.998	780.522
0.258	1	503.187
2.173	1.015	127.919
0.86	1.002	356.108
0.698	1.002	353.289
0.995	1.002	341.516
0.839	1.002	357.72
0.841	1.002	362.503
0.836	1.002	364.302
0.841	1.002	362.503
0.893	1.002	365.638
1.704	1.012	150.286
0.836	1.002	364.302
0.226	1.001	415.715
0.262	1.001	416.231
0.841	1.002	362.503
0.728	1.002	356.697
0.828	1.002	361.656
0.233	1.001	425.745
0.819	1.002	360.634
0.782	1.002	354.445
13.731	1.005	254.364
0.77	1.003	330.976
0.578	1.003	332.366
0.814	1.002	339.128
0.069	0.998	803.626
2.385	1.019	106.296
0.864	1.002	348.791
0.742	1.002	351.792
0.842	1.002	354.539
0.842	1.002	354.539
0.695	1.002	372.558
0.767	1.002	335.034
0.721	1.002	363.265
0.767	1.002	335.034
0.242	1.001	422.045
0.745	1.002	342.97
0.778	1.002	359.68

0.214	1.001	421.621
0.754	1.002	350.944
0.008	0.996	3853.667
0.778	1.002	359.68
0.228	1.001	427.033
0.695	1.002	372.558
0.745	1.002	342.969
0.185	1.001	444.14
0.151	1	466.795
0.833	1.002	369.267
0.543	1.002	342.916
0.767	1.002	335.034
0.123	0.999	556.334
0.777	1.002	362.739
0.139	1	538.172
0.887	1.002	370.416
1.794	1.013	138.943
1.925	1.015	126.645
5.271	1.031	73.615
0.956	1.005	248.241
3.727	0.999	626.892
0.842	1.002	350.193
27.604	1.003	328.335
0.165	1	539.011
0.791	1.003	301.694
0.821	1.003	333.609
7.455	1.015	126.359
0.822	1.004	276.148
0.919	1.004	270.386
9.014	1.005	244.625
3.015	1.019	105.277
0.861	1.004	292.908
0.845	1.003	304.454
0.803	1.003	298.186
0.78	1.003	305.393
0.907	1.003	307.432
0.813	1.002	344.865
0.774	1.003	324.735
0.153	1	496.515
0.813	1.002	353.437
15.746	1.005	257.712
6.789	1.027	81.659
4.389	1.006	223.781

0.702	1.011	157.229
0.8	1.002	371.492
0.755	1.002	350.808
2.264	0.999	607.178
0.894	1.002	348.142
0.18	1.001	444.733
0.193	1.001	427.444
0.018	0.997	1513.315
0.976	1.005	245.608
0.976	1.005	245.608
0.976	1.005	245.608
0.976	1.005	245.608
0.976	1.005	245.608
0.976	1.005	245.608
0.976	1.005	245.608
0.976	1.005	245.608
0.976	1.005	245.608
0.976	1.005	245.608
0.976	1.005	245.608
0.976	1.005	245.608
0.976	1.005	245.608
0.976	1.005	245.608
0.976	1.005	245.608
0.976	1.005	245.608
0.976	1.005	245.608
0.976	1.005	245.608
0.976	1.005	245.608
0.976	1.005	245.608
0.118	0.999	687.59
0.906	1.003	309.675
0.113	0.999	665.324
0.118	0.999	652.052
3.062	1.013	139.065
0.897	1.004	271.037
0.222	1.002	373.22
0.509	1.008	193.424
1.743	1.015	130.653
0.185	1.001	430.883
18.868	1.014	132.948
2.427	1.015	130.665
0.862	1.002	353.631
0.807	1.002	378.048
0.005	0.997	1616.903

0.767	1.002	335.034
0.724	1.002	362
0.164	1	476.408
4.893	1.022	94.238
0.778	1.002	359.68
0.761	1.001	387.187
0.695	1.002	372.558
0.218	1.001	412.348
0.745	1.002	342.97
0.989	1.002	344.674
0.754	1.002	350.944
0.745	1.002	342.97
0.192	1.001	429.392
0.745	1.002	342.97
0.754	1.002	350.944
1.909	1.015	130.411
0.837	1.003	334.498
0.778	1.002	359.68
1.678	1.012	153.332
0.543	1.002	342.916
0.695	1.002	372.558
0.297	1.001	445.343
0.824	1.002	366.755
0.915	1.002	354.721
0.837	1.003	334.498
0.767	1.002	335.034
0.12	1	508.719
0.543	1.002	342.916
0.745	1.002	342.97
0.754	1.002	350.944
0.767	1.002	335.034
0.721	1.002	363.265
0.815	1.002	368.124
0.778	1.002	359.68
0.915	1.002	354.721
0.767	1.002	335.034
0.208	1.001	423.599
0.778	1.002	359.68
0.837	1.003	334.498
0.767	1.002	335.034
0.745	1.002	342.97
0.837	1.003	334.498
0.767	1.002	335.034

0.684	1.002	376.272
0.747	1.002	342.205
0.778	1.002	359.68
0.745	1.002	342.97
0.833	1.002	369.267
0.778	1.002	359.68
0.754	1.002	350.944
0.915	1.002	354.721
0.879	1.002	372.756
0.837	1.003	334.498
0.133	1	549.126
0.695	1.002	372.558
2.207	1.015	126.354
0.005	1.001	392.112
0.778	1.002	359.68
0.563	1.01	165.617
0.808	1.002	372.435
0.767	1.002	335.034
0.887	1.002	370.416
0.695	1.002	372.558
0.116	1	534.01
0.915	1.002	354.721
17.501	1.002	345.423
2.191	1.015	125.963
0.811	1.002	343.86
0.796	1.002	375.081
0.846	1.002	364.759
0.846	1.002	364.759
0.842	1.002	354.539
0.116	1	534.01
0.842	1.002	354.539
0.27	0.999	556.134

BayesFactor	PSRF	Neff
0.572	1.011	160.709
2.175	1.013	141.271
0	1.005	240.977
39.995	0.998	843.233
0.893	1.011	158.449
0.729	1.011	161.396
2.757	1.015	127.303

2.411	1.014	137.362
0.08	1.002	364.113
0.902	1.011	158.195
0.962	1.011	159.664
0.886	1.011	158.661
0.902	1.011	158.195
2.218	1.013	139.791
0.29	1.018	112.914
0.89	1.011	158.372
0.294	1.02	104.808
1.54	1.011	162.255
2.191	1.013	140.724
0.03	1.002	355.715
0.029	1.002	348.719
0.631	1.011	162.676
0.782	1.011	160.12
0.535	1.01	173.406
3.531	1.013	140.044
1.678	1.011	157.874
0.06	1.002	343.893
2.391	1.004	271.035
1.495	1.006	235.478
0.866	1.011	159.075
0.68	1.011	161.981
1.019	1.011	157.889
1.019	1.011	157.889
0.661	1.011	162.252
0.665	1.011	162.168
1.735	1.011	154.937
1.807	1.012	151.22
0.789	1.011	160.189
2.391	1.014	137.481
0.68	1.011	161.981
0.782	1.011	160.12
0.23	1.015	128.571
0.685	1.011	161.929
0.902	1.011	158.195
0.773	1.011	160.392
0.889	1.011	158.571
0.665	1.011	162.168
0.759	1.011	160.579
0.789	1.011	160.189
0.682	1.011	161.953

0.716	1.011	161.899
0.572	1.011	160.709
0.118	1.009	178.214
0.782	1.011	160.12
0.104	1.009	181.358
0.572	1.011	160.709
0.875	1.011	159.021
1.628	1.011	158.762
2.319	1.014	135.758
3.431	1.013	141.395
1.939	1.012	146.708
1.681	1.011	157.477
0.68	1.011	161.981
0.888	1.011	158.604
0.733	1.011	161.782
4.368	1.015	129.526
0.876	1.011	158.978
0.782	1.011	160.12
0.919	1.012	147.553
2	1.013	145.568
2.386	1.014	137.792
2.713	1.015	129.24
0.888	1.011	158.604
0.888	1.011	158.604
0.68	1.011	161.981
0.459	1.013	139.399
0.201	1.012	154.368
0.789	1.011	160.189
0.782	1.011	160.12
0.665	1.011	162.168
0.665	1.011	162.168
0.827	1.011	159.258
0.759	1.011	160.579
0.33	1.02	104.04
0.152	1.005	257.369
1.867	1.012	149.403
0.572	1.011	160.709
0.759	1.011	160.579
0.685	1.011	161.929
0.634	1.011	158.515
1.846	1.012	150.418
0.928	1.011	159.138
0.665	1.011	162.168

0.572	1.011	160.709
0.665	1.011	162.168
0.759	1.011	160.579
0.945	1.011	160.338
0.26	1.015	127.121
0.789	1.011	160.189
0.789	1.011	160.189
0.716	1.011	161.899
0.572	1.011	160.709
0.759	1.011	160.579
0.066	1.008	192.899
0.789	1.011	160.189
0.665	1.011	162.168
0.194	1.013	142.879
0.782	1.011	160.12
0.955	1.011	156.621
0.789	1.011	160.189
0.68	1.011	161.981
0.782	1.011	160.12
0.939	1.011	157.324
0.572	1.011	160.709
0.89	1.011	158.372
0.789	1.011	160.189
0.789	1.011	160.189
0.674	1.011	162.02
1.816	1.012	151.649
0.928	1.011	159.138
0.759	1.011	160.579
0.902	1.011	158.195
0.572	1.011	160.709
0.685	1.011	161.929
0.955	1.011	156.621
0.242	1.016	122.816
0.939	1.011	157.324
0.789	1.011	160.189
0.815	1.011	159.62
1.603	1.011	161.246
0.196	1.013	139.241
0.157	1.011	156.747
2.392	1.013	138.194
0.948	1.011	160.23
4.255	1.013	139.544
57.935	0.998	811.953

0.665	1.011	162.168
0.149	1.01	172.668
0.955	1.011	156.621
0.128	1.01	170.542
0.052	1.004	281.17
0.803	1.011	160.044
0.228	1.015	126.576
0.875	1.011	159.021
0.928	1.011	159.138
0.665	1.011	162.168
