# Supplementary material for: Molecular evolution of genes encoding allergen proteins in the peanuts genus Arachis: Structural and functional implications
Source: PLoS One. 2019 Nov 1;14(11):e0222440. doi: 10.1371/journal.pone.0222440 (PMC6824556; doi:10.1371/journal.pone.0222440)
Supplement: S1 Fig — Epitopes are mapped on the top of the alignments. Ara h 2 alignment is shown first followed by Ara h 6 after the stop codon of Ara h 2. The notations gb, gb 2 and gen refer to GenBank sequence accessions. The Ara h 2 sequences of A. monticola, A. helodes and A. trintensis are missing the upstream 5’ region 33 amino acid residues and the first codon position of the 34th residue and consequently the latter codon was noted in the translation by the program as “&P&C”. The Ara h 6 sequences of A. ipaensis and A. duranensis are missing the first 17 and 14 amino acid residues, respectively, and the symbols in the next downstream residue denote the absence of their first codon positions. (PDF) [file pone.0222440.s004.pdf]

Supplementary fig. S1. Amino acid alignments of Ara h2 and Ara h 6 for the *Arachis* species examined in this study deduced from the nucleotide sequence alignment. Epitopes are mapped on the top of the alignments. Ara h 2 alignment is shown first followed by Ara h 6 after the stop codon of Ara h 2. the notations gb, gb 2 and gen refer to sequence accessions obtained from GenBank.

Epitope 1 Epitope 2 Epitope 3 Epitope 4

Characters	1	2	3	4	5	6	7	8	9	10	11	12	13	14	15	16	17	18	19	20	21	22	23	24	25
	1.1	1.2	1.3	1.4	1.5	1.6	1.7	1.8	1.9	1.10	1.11	1.12	1.13	1.14	1.15	1.16	1.17	1.18	1.19	1.20	1.21	1.22	1.23	1.24	1.25
A. ipaensis	M	-	-	A	K	L	T	I	L	V	A	L	A	L	F	L	L	A	A	H	A	S	A	R	Q
A. ipaensis gb	M	-	-	A	K	L	T	I	L	V	A	L	A	L	F	L	L	A	A	H	A	S	A	R	Q
A. ipaensis gb2	M	-	-	A	K	L	T	I	L	V	A	L	A	L	F	L	L	A	A	H	A	S	A	R	Q
A. duranensis	M	-	-	A	K	L	T	I	L	V	A	L	A	L	F	L	L	A	A	H	A	S	A	R	Q
A. duranensis gen	M	-	-	A	K	L	T	I	L	V	A	L	A	L	F	L	L	A	A	H	A	S	A	R	Q
A. hypogaea	M	-	-	A	K	L	T	I	L	V	A	L	A	L	F	L	L	A	A	H	A	S	A	R	Q
A. hypogaea 2.01 gb	M	-	-	-	-	L	T	I	L	V	A	L	A	L	F	L	L	A	A	H	A	S	A	R	Q
A. hypogaea 2.02 gb	M	-	-	A	K	L	T	I	L	V	A	L	A	L	F	L	L	A	A	H	A	S	A	R	Q
A. monticola	?	?	?	?	?	?	?	?	?	?	?	?	?	?	?	?	?	?	?	?	?	?	?	?	?
A. helodes	?	?	?	?	?	?	?	?	?	?	?	?	?	?	?	?	?	?	?	?	?	?	?	?	?
A. glandulifera	M	-	-	S	N	L	T	I	L	V	A	L	A	L	F	L	L	A	A	H	A	S	A	R	H
A. hoehnei	M	P	M	A	K	L	T	I	L	V	A	L	A	L	F	L	L	A	A	H	A	S	A	R	Q
A. batizocoi	M	-	-	S	K	L	T	I	L	V	A	L	A	L	F	L	L	A	A	H	A	S	A	R	H
A. trinitensis	?	?	?	?	?	?	?	?	?	?	?	?	?	?	?	?	?	?	?	?	?	?	?	?	?
A. praecox	M	P	I	P	K	L	T	I	L	V	A	L	A	L	F	L	L	A	A	H	A	S	A	R	H
A. palustris	M	-	-	A	K	L	T	I	L	V	A	L	A	L	F	L	L	A	A	H	A	S	A	R	Q
A. kretschmeri	M	P	M	A	K	L	T	I	L	V	A	L	A	L	F	L	L	A	A	H	A	S	A	R	Q
A. glabrata	M	-	-	S	K	L	T	I	L	V	A	L	A	L	F	L	L	A	A	H	A	S	A	R	H
A. appressipila	M	-	-	A	K	L	T	I	L	V	A	L	A	L	F	L	L	A	A	H	A	S	A	R	Q
A. tuberosa	M	-	-	A	K	L	T	I	L	V	A	L	A	L	F	L	L	A	A	H	A	S	A	R	Q
A. guaranitica	M	-	-	A	K	L	T	I	L	V	A	L	A	L	F	L	L	A	A	H	A	S	A	R	Q
A. rigonii	M	-	-	A	K	L	T	I	L	V	A	L	A	L	F	L	L	A	A	H	A	S	A	R	Q
A. dardani	M	-	-	S	K	L	T	I	L	V	A	L	A	L	F	L	L	A	A	H	A	S	A	R	Q
A. paraguariensis	M	-	-	S	K	F	T	I	L	V	A	L	A	L	F	L	L	A	A	H	A	S	A	R	Q
A. pintoi	M	-	-	S	K	L	T	I	L	V	A	L	A	L	F	L	L	A	A	H	A	S	A	R	Q
A. triseminata	M	-	-	A	K	L	T	I	L	V	A	L	A	L	L	L	L	A	A	H	A	S	A	R	Q
A. lutescens	M	-	-	A	K	L	T	I	L	V	A	L	A	L	F	L	L	A	A	H	A	S	A	R	H
A. macedoi	M	-	-	S	K	L	T	I	L	V	A	L	A	L	F	L	L	A	A	H	A	S	A	R	Q

Characters	26	27	28	29	30	31	32	33	34	35	36	37	38	39	40	41	42	43	44	45	46	47	48	49	50
	1.26	1.27	1.28	1.29	1.30	1.31	1.32	1.33	1.34	1.35	1.36	1.37	1.38	1.39	1.40	1.41	1.42	1.43	1.44	1.45	1.46	1.47	1.48	1.49	1.50
A. ipaensis	Q	W	E	L	Q	G	D	R	R	C	Q	S	Q	L	E	R	A	N	L	R	P	C	E	Q	H
A. ipaensis gb	Q	W	E	L	Q	G	D	R	R	C	Q	S	Q	L	E	R	A	N	L	R	P	C	E	Q	H
A. ipaensis gb2	Q	W	E	L	Q	G	D	R	R	C	Q	S	Q	L	E	R	A	N	L	R	P	C	E	Q	H
A. duranensis	Q	W	E	L	Q	G	D	R	R	C	Q	S	Q	L	E	R	A	N	L	R	P	C	E	Q	H
A. duranensis gen	Q	W	E	L	Q	G	D	R	R	C	Q	S	Q	L	E	R	A	N	L	R	P	C	E	Q	H
A. hypogaea	Q	W	E	L	Q	G	D	R	R	C	Q	S	Q	L	E	R	A	N	L	R	P	C	E	Q	H
A. hypogaea 2.01 gb	Q	W	E	L	Q	G	D	R	R	C	Q	S	Q	L	E	R	A	N	L	R	P	C	E	Q	H
A. hypogaea 2.02 gb	Q	W	E	L	Q	G	D	R	R	C	Q	S	Q	L	E	R	A	N	L	R	P	C	E	Q	H
A. monticola	?	?	?	?	?	?	?	?	&P&C	C	Q	S	Q	L	E	R	A	N	L	R	P	C	E	Q	H
A. helodes	?	?	?	?	?	?	?	?	&P&C	C	Q	S	Q	L	E	R	A	N	L	R	P	C	E/Q	Q	H
A. glandulifera	Q	W	E	L	Q	G	D	R	R	C	Q	S	Q	L	E	R	A	N	L	R	P	C	E	Q	H
A. hoehnei	Q	W	E	L	Q	G	D	R	R	C	Q	S	Q	L	E	R	A	N	L	R	P	C	E	Q	H
A. batizocoi	Q	W	E	L	Q	G	D	R	R	C	Q	S	Q	L	E	R	A	N	L	R	P	C	E	Q	H
A. trinitensis	?	?	?	?	?	?	?	?	&P&C	C	Q	S	Q	L	E	R	A	N	L	R	P	C	E	Q	H
A. praecox	Q	W	E	L	Q	G	D	R	R	C	Q	S	Q	L	E	R	A	N	L	R	P	C	E	Q	H
A. palustris	Q	W	E	L	Q	G	D	R	R	C	Q	S	Q	L	E	R	A	N	L	R	P	C	E	Q	H
A. kretschmeri	Q	W	E	L	Q	G	D	R	R	C	Q	S	Q	L	E	R	A	N	L	R	P	C	E	Q	H
A. glabrata	Q	W	E	L	Q	G	D	R	R	C	Q	S	Q	L	E	R	A	N	L	R	P	C	E	Q	H
A. appressipila	Q	W	E	L	Q	G	D	R	R	C	Q	S	Q	L	E	R	A	N	L	R	P	C	E	Q	H
A. tuberosa	Q	W	E	L	Q	G	D	R	R	C	Q	S	Q	L	E	R	A	N	L	R	P	C	E	Q	H
A. guaranitica	Q	W	E	L	Q	G	D	R	R	C	Q	S	Q	L	E	R	A	N	L	R	P	C	E	Q	H
A. rigonii	Q	W	E	L	Q	G	D	R	R	C	Q	S	Q	L	E	R	A	N	L	R	P	C	E	Q	H
A. dardani	Q	W	E	L	Q	G	D	R	R	C	Q	S	Q	L	E	R	A	N	L	R	P	C	E	Q	H
A. paraguariensis	Q	W	E	L	Q	G	D	R	R	C	Q	S	Q	L	E	R	A	N	L	R	P	C	E	Q	H
A. pintoi	Q	W	E	L	R	G	D	R	R	C	Q	S	Q	L	E	R	A	N	L	R	P	C	E	Q	H
A. triseminata	Q	W	E	L	Q	G	D	R	R	C	Q	S	Q	L	E	R	A	N	L	R	P	C	E	Q	H
A. lutescens	Q	W	E	L	Q	G	D	R	R	C	Q	S	Q	L	E	R	A	N	L	R	P	C	E	Q	H
A. macedoi	Q	W	E	L	Q	G	D	R	R	C	Q	S	Q	L	E	R	A	N	L	R	P	C	E	Q	H

Epitope 5 Epitope 6 Epitope 7

Characters	51	52	53	54	55	56	57	58	59	60	61	62	63	64	65	66	67	68	69	70	71	72	73	74	75
	1.51	1.52	1.53	1.54	1.55	1.56	1.57	1.58	1.59	1.60	1.61	1.62	1.63	1.64	1.65	1.66	1.67	1.68	1.69	1.70	1.71	1.72	1.73	1.74	1.75
A. ipaensis	L	M	Q	K	I	Q	R	D	E	D	-	S	Y	G	R	D	P	Y	S	P	S	Q	D	P	Y
A. ipaensis gb	L	M	Q	K	I	Q	R	D	E	D	-	S	Y	G	R	D	P	Y	S	P	S	Q	D	P	Y
A. ipaensis gb2	L	M	Q	K	I	Q	R	D	E	D	-	S	Y	G	R	D	P	Y	S	P	S	Q	D	P	Y
A. duranensis	L	M	Q	K	I	Q	R	D	E	D	-	S	Y	E	R	D	P	Y	S	P	S	Q	D	P	Y
A. duranensis gen	L	M	Q	K	I	Q	R	D	E	D	-	S	Y	E	R	D	P	Y	S	P	S	Q	D	P	Y
A. hypogaea	L	M	Q	K	I	Q	R	D	E	D	-	S	Y	E	R	D	P	Y	S	P	S	Q	D	P	Y
A. hypogaea 2.01 gb	L	M	Q	K	I	Q	R	D	E	D	-	S	Y	E	R	D	P	Y	S	P	S	Q	D	P	Y
A. hypogaea 2.02 gb	L	M	Q	K	I	Q	R	D	E	D	-	S	Y	G	R	D	P	Y	S	P	S	Q	D	P	Y
A. monticola	L	M	Q	K	I	Q	R	D	E	D	-	S	Y	E	R	D	P	Y	S	P	S	Q	D	P	Y
A. helodes	L	M	Q	K	I	Q	R	D	E	D	-	S	Y	E	R	D	P	Y	S	P	S	Q	D	P	Y
A. glandulifera	L	M	Q	K	I	Q	R	D	E	D	-	S	Y	G	R	D	P	D	R	-	-	Q	D	P	Y
A. hoehnei	L	M	Q	K	I	Q	R	D	E	D	-	S	Y	E	R	D	P	Y	S	P	S	-	-	-	-
A. batizocoi	L	M	Q	K	I	Q	R	D	E	D	-	S	Y	G	R	D	P	Y	S	P	S	-	-	-	-
A. trinitensis	L	M	Q	K	I	Q	R	D	E	D	-	S	Y	G	R	D	P	D	R	-	-	E	D	P	Y
A. praecox	L	M	Q	K	I	Q	R	D	E	D	S	S	Y	G	R	D	P	D	R	-	-	E	D	P	Y
A. palustris	L	M	Q	K	I	Q	R	D	E	D	S	S	Y	G	R	D	P	D	R	-	-	E	D	P	Y
A. kretschmeri	L	M	Q	K	I	Q	R	D	E	D	-	S	Y	E	R	D	P	Y	S	P	S	-	-	-	-
A. glabrata	L	M	Q	K	I	Q	R	D	E	D	-	S	Y	G	R	D	P	Y	S	P	S	-	-	-	-
A. appressipila	L	M	Q	K	I	Q	R	D	E	D	-	S	Y	E	-	-	-	-	-	-	-	-	-	-	-
A. tuberosa	L	M	Q	K	I	Q	R	D	E	D	-	S	Y	E	-	-	-	-	-	-	-	-	-	-	-
A. guaranitica	L	M	Q	K	I	Q	R	D	E	D	-	S	Y	E	-	-	-	-	-	-	-	-	-	-	-
A. rigonii	L	M	Q	K	I	Q	R	D	E	D	-	S	Y	E	-	-	-	-	-	-	-	-	-	-	-
A. dardani	L	M	Q	K	I	Q	R	D	E	D	-	S	Y	E	-	-	-	-	-	-	-	-	-	-	-
A. paraguariensis	L	M	Q	K	I	Q	R	D	E	D	-	S	Y	E	-	-	-	-	-	-	-	-	-	-	-
A. pintoi	L	M	Q	K	I	Q	R	E	E	D	-	Q	Y	E	-	-	-	-	-	-	-	-	-	-	-
A. triseminata	L	M	Q	K	I	Q	R	D	Q	S	-	-	-	-	-	-	-	-	-	-	-	-	-	P	Y
A. lutescens	L	M	Q	K	I	Q	R	D	E	D	-	S	Y	E	R	D	P	Y	S	P	S	-	-	-	-
A. macedoi	L	M	Q	K	I	Q	R	D	Q	D	-	Q	Y	E	-	-	-	-	-	-	-	-	-	-	-

Characters	76	77	78	79	80	81	82	83	84	85	86	87	88	89	90	91	92	93	94	95	96	97	98	99	100
	1.76	1.77	1.78	1.79	1.80	1.81	1.82	1.83	1.84	1.85	1.86	1.87	1.88	1.89	1.90	1.91	1.92	1.93	1.94	1.95	1.96	1.97	1.98	1.99	1.10
A. ipaensis	S	P	S	Q	D	P	D	R	R	D	P	Y	S	P	S	P	Y	-	-	-	-	-	-	-	-
A. ipaensis gb	S	P	S	-	-	-	-	-	-	-	-	-	-	-	-	P	Y	-	-	-	-	-	-	-	-
A. ipaensis gb2	S	P	S	Q	D	P	D	R	R	D	P	Y	S	P	S	P	Y	-	-	-	-	-	-	-	-
A. duranensis	S	P	S	-	-	-	-	-	-	-	-	-	-	-	-	P	Y	-	-	-	-	-	-	-	-
A. duranensis gen	S	P	S	-	-	-	-	-	-	-	-	-	-	-	-	P	Y	-	-	-	-	-	-	-	-
A. hypogaea	S	P	S	-	-	-	-	-	-	-	-	-	-	-	-	P	Y	-	-	-	-	-	-	-	-
A. hypogaea 2.01 gb	S	P	S	-	-	-	-	-	-	-	-	-	-	-	-	P	Y	-	-	-	-	-	-	-	-
A. hypogaea 2.02 gb	S	P	S	Q	D	P	D	R	R	D	P	Y	S	P	S	P	Y	-	-	-	-	-	-	-	-
A. monticola	S	P	S	Q	?	?	&L&I	S	Q	D	P	Y	S	P	S	P	Y	-	-	-	-	-	-	-	-
A. helodes	S	P	S	-	-	-	-	-	Q	D	P	Y	S	P	S	P	Y	-	-	-	-	-	-	-	-
A. glandulifera	S	P	S	Q	D	P	D	R	Q	D	P	Y	S	P	S	P	Y	-	-	-	-	-	-	-	-
A. hoehnei	-	-	-	Q	D	P	Y	R	Q	D	P	Y	T	P	S	P	Y	-	-	-	-	-	-	-	-
A. batizocoi	-	-	-	Q	D	P	Y	K	Q	D	P	Y	T	P	S	P	Y	-	-	-	-	-	-	-	-
A. trinitensis	S	P	S	Q	D	P	D	R	Q	D	P	Y	S	P	S	P	Y	-	-	-	-	-	-	-	-
A. praecox	S	P	S	Q	D	P	D	R	E	D	P	Y	S	P	S	P	Y	-	-	-	-	-	-	-	-
A. palustris	S	P	S	Q	D	P	D	R	E	D	P	Y	S	P	S	P	Y	-	-	-	-	-	-	-	-
A. kretschmeri	-	-	-	Q	D	P	Y	R	Q	D	P	Y	S	P	S	P	Y	-	-	-	-	-	-	-	-
A. glabrata	-	-	-	Q	D	P	Y	K	Q	D	P	Y	T	P	S	P	Y	-	-	-	-	-	-	-	-
A. appressipila	-	-	-	-	-	-	-	-	Q	D	P	Y	G	P	S	P	Y	-	-	-	-	-	-	-	-
A. tuberosa	-	-	-	Q	D	P	Y	R	Q	D	P	Y	G	P	S	P	Y	G	P	S	P	Y	G	P	S
A. guaranitica	-	-	-	Q	D	P	Y	R	Q	D	P	Y	G	P	S	P	Y	-	-	-	-	-	-	-	-
A. rigonii	-	-	-	-	-	-	-	-	Q	D	P	Y	S	P	S	P	Y	-	-	-	-	-	-	-	-
A. dardani	-	-	-	-	-	-	-	-	Q	D	P	Y	G	P	S	P	Y	-	-	-	-	-	-	-	-
A. paraguariensis	-	-	-	-	-	-	-	-	Q	D	P	Y	G	P	S	P	Y	-	-	-	-	-	-	-	-
A. pintoi	-	-	-	-	-	-	-	-	Q	D	P	Y	S	P	S	P	Y	-	-	-	-	-	-	-	-
A. triseminata	S	-	-	Q	D	P	Y	R	Q	E	P	Y	E	-	-	-	Y	-	-	-	-	-	-	-	-
A. lutescens	-	-	-	Q	D	P	Y	R	Q	D	P	Y	S	P	S	P	Y	-	-	-	-	-	-	-	-
A. macedoi	-	-	-	Q	D	P	Y	R	Q	D	P	Y	-	-	-	-	-	-	-	-	-	-	-	-	-

Characters	101	102	103	104	105	106	107	108	109	110	111	112	113	114	115	116	117	118	119	120	121	122	123	124	125	126	127	128	129	130	131	132	133	134	135	136	137	138	139	140	141	142	143	144	145	146	147	148	149	150
	1.10	1.10	1.10	1.10	1.10	1.10	1.10	1.10	1.10	1.10	1.11	1.11	1.11	1.11	1.11	1.11	1.11	1.11	1.11	1.11	1.12	1.12	1.12	1.12	1.12	1.12	1.12	1.12	1.12	1.12	1.13	1.13	1.13	1.13	1.13	1.13	1.13	1.13	1.13	1.13	1.14	1.14	1.14	1.14	1.14	1.14	1.14	1.14	1.14	1.14
A. ipaensis	-	-	-	-	-	-	-	-	-	-	-	-	-	-	-	-	-	-	-	-	D	R	R	G	A	G	S	S	Q	H	Q	E	R	C	C	N	E	L	N	E	F	E	N	N	Q	R	C	M	C	E
A. ipaensis gb	-	-	-	-	-	-	-	-	-	-	-	-	-	-	-	-	-	-	-	-	D	R	R	G	A	G	S	S	Q	H	Q	E	R	C	C	N	E	L	N	E	F	E	N	N	Q	R	C	M	C	E
A. ipaensis gb2	-	-	-	-	-	-	-	-	-	-	-	-	-	-	-	-	-	-	-	-	D	R	R	G	A	G	S	S	Q	H	Q	E	R	C	C	N	E	L	N	E	F	E	N	N	Q	R	C	M	C	E
A. duranensis	-	-	-	-	-	-	-	-	-	-	-	-	-	-	-	-	-	-	-	-	D	R	R	G	A	G	S	S	Q	H	Q	E	R	C	C	N	E	L	N	E	F	E	N	N	Q	R	C	M	C	E
A. duranensis gen	-	-	-	-	-	-	-	-	-	-	-	-	-	-	-	-	-	-	-	-	D	R	R	G	A	G	S	S	Q	H	Q	E	R	C	C	N	E	L	N	E	F	E	N	N	Q	R	C	M	C	E
A. hypogaea	-	-	-	-	-	-	-	-	-	-	-	-	-	-	-	-	-	-	-	-	D	R	R	G	A	G	S	S	Q	H	Q	E	R	C	C	N	E	L	N	E	F	E	N	N	Q	R	C	M	C	E
A. hypogaea 2.01 gb	-	-	-	-	-	-	-	-	-	-	-	-	-	-	-	-	-	-	-	-	D	R	R	G	A	G	S	S	Q	H	Q	E	R	C	C	N	E	L	N	E	F	E	N	N	Q	R	C	M	C	E
A. hypogaea 2.02 gb	-	-	-	-	-	-	-	-	-	-	-	-	-	-	-	-	-	-	-	-	D	R	R	G	A	G	S	S	Q	H	Q	E	R	C	C	N	E	L	N	E	F	E	N	N	Q	R	C	M	C	E
A. monticola	-	-	-	-	-	-	-	-	-	-	-	-	-	-	-	-	-	-	-	-	D	R	R	G	A	G	S	S	Q	H	Q	E	R	C	C	N	E	L	N	E	F	E	N	N	Q	R	C	M	C	E
A. helodes	-	-	-	-	-	-	-	-	-	-	-	-	-	-	-	-	-	-	-	-	D	R	R	G	A	G	S	S	Q	H	Q	E	R	C	C	N	E	L	N	E	F	E	N	N	Q	R	C	M	C	E
A. glandulifera	-	-	-	-	-	-	-	-	-	-	-	-	-	-	-	-	-	-	-	-	D	R	R	G	A	G	S	S	Q	H	Q	E	R	C	C	N	E	L	N	E	F	E	N	N	Q	R	C	M	C	E
A. hoehnei	-	-	-	-	-	-	-	-	-	-	-	-	-	-	-	-	-	-	-	-	D	R	R	G	A	G	S	S	Q	H	Q	E	R	C	C	N	E	L	N	E	F	E	N	N	Q	R	C	M	C	E
A. batizocoi	-	-	-	-	-	-	-	-	-	-	-	-	-	-	-	-	-	-	-	-	D	E	R	R	A	G	S	S	Q	H	Q	E	R	C	C	N	E	L	N	E	F	E	N	N	Q	R	C	M	C	E
A. trinitensis	-	-	-	-	-	-	-	-	-	-	-	-	-	-	-	-	-	-	-	-	D	R	R	R	A	G	S	S	Q	H	Q	E	R	C	C	N	E	L	N	E	F	E	N	N	Q	R	C	M	C	E
A. praecox	-	-	-	-	-	-	-	-	-	-	-	-	-	G	P	S	P	-	-	Y	A	R	R	R	A	G	S	S	Q	H	Q	E	R	C	C	N	E	L	N	E	F	E	N	N	Q	R	C	M	C	E
A. palustris	-	-	-	-	-	-	-	-	-	-	-	-	-	G	P	S	P	-	-	Y	A	R	R	R	A	G	S	S	Q	H	Q	E	R	C	C	N	E	L	N	E	F	E	N	N	Q	R	C	M	C	E
A. kretschmeri	-	-	-	-	-	-	-	-	-	-	-	-	-	-	-	-	-	-	-	-	D	R	R	G	A	G	S	S	Q	H	Q	E	R	C	C	N	E	L	N	E	F	E	N	N	Q	R	C	M	C	E
A. glabrata	-	-	-	-	-	-	-	-	-	-	-	-	-	-	-	-	-	-	-	-	D	E	R	R	A	G	S	S	Q	H	Q	E	R	C	C	N	E	L	N	E	F	E	N	N	Q	R	C	M	C	E
A. appressipila	-	-	-	-	-	-	-	-	-	-	-	-	-	G	P	S	P	-	-	-	-	-	R	R	A	G	S	S	Q	H	Q	Q	R	C	C	N	E	L	N	E	F	E	N	D	Q	R	C	M	C	E
A. tuberosa	S	P	Y	G	P	S	P	Y	G	P	S	P	Y	G	P	S	P	-	-	-	-	-	R	R	A	G	S	S	Q	H	Q	Q	R	C	C	N	E	L	N	E	F	E	N	D	Q	R	C	M	C	E
A. guaranitica	-	-	-	-	-	-	-	-	-	-	-	-	-	G	P	S	P	-	-	-	-	-	R	R	A	G	S	S	Q	H	Q	Q	R	C	C	N	E	L	N	E	F	E	N	D	Q	R	C	M	C	E
A. rigonii	-	-	-	-	-	-	-	-	-	-	-	-	-	G	P	S	P	-	-	-	-	-	R	R	A	G	S	S	Q	H	Q	Q	R	C	C	N	E	L	N	E	F	E	N	D	Q	R	C	M	C	E
A. dardani	-	-	-	-	-	-	-	-	-	-	-	-	-	G	P	S	P	-	-	-	-	-	R	R	A	G	S	S	Q	H	Q	Q	R	C	C	N	E	L	N	E	F	E	N	D	Q	R	C	M	C	E
A. paraguariensis	-	-	-	-	-	-	-	-	-	-	-	-	-	G	P	S	P	-	-	-	-	-	R	R	A	G	S	S	Q	H	Q	Q	R	C	C	N	E	L	N	E	F	E	N	D	Q	R	C	M	C	E
A. pintoi	-	-	-	-	-	-	-	-	-	-	-	-	-	G	P	S	P	-	-	Y	D	R	R	H	A	G	S	S	Q	H	Q	Q	R	C	C	N	E	L	N	E	F	E	N	N	Q	R	C	M	C	E
A. triseminata	-	-	-	-	-	-	-	-	-	-	-	-	-	-	-	-	-	E	S	H	D	R	R	R	A	G	S	S	Q	H	Q	E	R	C	C	N	E	L	N	E	F	E	N	N	Q	R	C	M	C	Q
A. lutescens	-	-	-	-	-	-	-	-	-	-	-	-	-	-	-	-	-	-	-	-	D	R	R	G	A	G	S	S	Q	H	Q	E	R	C	C	N	E	L	N	E	F	E	N	N	Q	R	R	M	C	E
A. macedoi	-	-	-	-	-	-	-	-	-	-	-	-	-	-	-	-	-	D	S	Y	D	R	R	H	T	G	S	S	Q	H	Q	E	R	C	C	N	E	L	N	E	F	E	N	N	Q	R	C	M	C	Q

Characters	151	152	153	154	155	156	157	158	159	160	161	162	163	164	165	166	167	168	169	170	171	172	173	174	175
													Epitope 8										Epitope 9		
	1.15	1.15	1.15	1.15	1.15	1.15	1.15	1.15	1.15	1.15	1.16	1.16	1.16	1.16	1.16	1.16	1.16	1.16	1.16	1.16	1.17	1.17	1.17	1.17	1.17
A. ipaensis	A	L	Q	Q	I	M	E	N	Q	S	D	R	L	Q	G	R	Q	Q	E	Q	Q	F	K	R	E
A. ipaensis gb	A	L	Q	Q	I	M	E	N	Q	S	D	R	L	Q	G	R	Q	Q	E	Q	Q	F	K	R	E
A. ipaensis gb2	A	L	Q	Q	I	M	E	N	Q	S	D	R	L	Q	G	R	Q	Q	E	Q	Q	F	K	R	E
A. duranensis	A	L	Q	Q	I	M	E	N	Q	S	D	R	L	Q	G	R	Q	Q	E	Q	Q	F	K	R	E
A. duranensis gen	A	L	Q	Q	I	M	E	N	Q	S	D	R	L	Q	G	R	Q	Q	E	Q	Q	F	K	R	E
A. hypogaea	A	L	Q	Q	I	M	E	N	Q	S	D	R	L	Q	G	R	Q	Q	E	Q	Q	F	K	R	E
A. hypogaea 2.01 gb	A	L	Q	Q	I	M	E	N	Q	S	D	R	L	Q	G	R	Q	Q	E	Q	Q	F	K	R	E
A. hypogaea 2.02 gb	A	L	Q	Q	I	M	E	N	Q	S	D	R	L	Q	G	R	Q	Q	E	Q	Q	F	K	R	E
A. monticola	A	L	Q	Q	I	M	E	N	Q	S	D	R	L	Q	G	R	Q	Q	E	Q	Q	F	K	R	E
A. helodes	A	L	Q	Q	I	M	E	N	Q	S	D	R	L	Q	G	R	Q	Q	E	Q	Q	F	K	R	E
A. glandulifera	A	L	Q	Q	I	M	E	N	Q	S	D	R	L	Q	G	R	Q	Q	E	Q	Q	F	K	R	E
A. hoehnei	A	L	Q	Q	I	M	E	N	Q	S	D	R	L	Q	G	R	Q	Q	E	Q	Q	F	K	R	E
A. batizocoi	A	L	Q	Q	I	M	E	N	Q	S	D	R	L	Q	G	R	Q	Q	E	Q	Q	F	K	R	E
A. trinitensis	A	L	Q	Q	I	M	E	N	Q	S	D	R	L	Q	G	R	Q	Q	E	Q	Q	F	K	R	E
A. praecox	A	L	Q	Q	I	M	E	N	Q	S	D	R	L	Q	G	R	Q	Q	E	Q	Q	F	K	R	E
A. palustris	A	L	Q	Q	I	M	E	N	Q	S	D	R	L	Q	G	R	Q	Q	E	Q	Q	F	K	R	E
A. kretschmeri	A	L	Q	Q	I	M	E	N	Q	S	D	R	L	Q	G	R	Q	Q	E	Q	Q	F	K	R	E
A. glabrata	A	L	Q	Q	I	M	E	N	Q	S	D	R	L	Q	G	R	Q	Q	E	Q	Q	F	K	R	E
A. appressipila	A	L	Q	Q	I	M	E	N	Q	S	D	R	L	Q	G	R	Q	Q	E	Q	Q	F	K	R	E
A. tuberosa	A	L	Q	Q	I	M	E	N	Q	S	D	R	L	Q	G	R	Q	Q	E	Q	Q	F	K	R	E
A. guaranitica	A	L	Q	Q	I	M	E	N	Q	S	D	R	L	Q	G	R	Q	Q	E	Q	Q	F	K	R	E
A. rigonii	A	L	Q	Q	I	M	E	N	Q	S	D	R	L	Q	G	R	Q	Q	E	Q	Q	F	K	R	E
A. dardani	A	L	Q	Q	I	M	E	N	Q	S	D	R	L	Q	G	R	Q	Q	E	Q	Q	F	K	R	E
A. paraguariensis	A	L	Q	Q	I	M	E	N	Q	S	D	R	L	Q	G	R	Q	Q	E	Q	Q	F	K	R	E
A. pintoi	A	L	Q	Q	I	M	E	N	Q	S	D	R	L	Q	G	R	Q	Q	E	Q	Q	F	K	R	E
A. triseminata	A	L	Q	Q	I	M	E	N	Q	S	D	R	L	Q	G	R	Q	Q	E	Q	Q	F	K	R	E
A. lutescens	A	L	Q	Q	I	M	E	N	Q	S	D	R	L	Q	G	R	Q	Q	E	Q	Q	F	K	R	E
A. macedoi	A	L	Q	Q	I	M	E	N	Q	S	D	R	L	Q	G	R	Q	Q	E	Q	Q	F	K	R	E

Characters	176	177	178	179	180	181	182	183	184	185	186	187	188	189	190	191	192	193	194	195	196	197	198	199	200
														Epitope 10											
	1.17	1.17	1.17	1.17	1.17	1.18	1.18	1.18	1.18	1.18	1.18	1.18	1.18	1.18	1.18	1.19	1.19	1.19	1.19	1.19	1.19	1.19	1.19	1.19	1.19
A. ipaensis	L	R	N	L	P	Q	Q	C	G	L	R	A	P	Q	R	C	D	L	E	V	E	S	G	G	R
A. ipaensis gb	L	R	N	L	P	Q	Q	C	G	L	R	A	P	Q	R	C	D	L	E	V	E	S	G	G	R
A. ipaensis gb2	L	R	N	L	P	Q	Q	C	G	L	R	A	P	Q	R	C	D	L	E	V	E	S	G	G	R
A. duranensis	L	R	N	L	P	Q	Q	C	G	L	R	A	P	Q	R	C	D	L	D	V	E	S	G	G	R
A. duranensis gen	L	R	N	L	P	Q	Q	C	G	L	R	A	P	Q	R	C	D	L	D	V	E	S	G	G	R
A. hypogaea	L	R	N	L	P	Q	Q	C	G	L	R	A	P	Q	R	C	D	L	D	V	E	S	G	G	R
A. hypogaea 2.01 gb	L	R	N	L	P	Q	Q	C	G	L	R	A	P	Q	R	C	D	L	D	V	E	S	G	G	R
A. hypogaea 2.02 gb	L	R	N	L	P	Q	Q	C	G	L	R	A	P	Q	R	C	D	L	E	V	E	S	G	G	R
A. monticola	L	R	N	L	P	Q	Q	C	G	L	R	A	P	Q	R	C	D	L	E	V	E	S	G	G	R
A. helodes	L	R	N	L	P	Q	Q	C	G	L	R	A	P	Q	R	C	D	L	D	V	E	S	G	G	R
A. glandulifera	L	R	N	L	P	Q	Q	C	G	L	R	A	P	Q	R	C	D	L	D	V	E	S	G	G	R
A. hoehnei	L	R	N	L	P	Q	Q	C	G	L	R	A	P	Q	R	C	D	L	D	V	E	S	G	G	R
A. batizocoi	L	R	N	L	P	Q	Q	C	G	L	R	A	P	Q	R	C	D	L	D	V	E	S	G	G	R
A. trinitensis	L	R	N	L	P	Q	Q	C	G	L	R	A	P	Q	R	C	D	L	E	V	E	S	G	G	R
A. praecox	L	R	N	L	P	Q	Q	C	G	L	R	A	P	Q	R	C	D	L	D	V	E	S	G	G	R
A. palustris	L	R	N	L	P	Q	Q	C	G	L	R	A	P	Q	R	C	D	L	D	V	E	S	G	G	R
A. kretschmeri	L	R	N	L	P	Q	Q	C	G	L	R	A	P	Q	R	C	D	L	D	V	E	S	G	G	R
A. glabrata	L	R	N	L	P	Q	Q	C	G	L	R	A	P	Q	R	C	D	L	D	V	E	S	G	G	R
A. appressipila	L	R	N	L	P	Q	Q	C	D	L	R	A	P	Q	R	C	D	L	D	V	E	S	G	G	R
A. tuberosa	L	R	N	L	P	Q	Q	C	D	L	R	A	P	Q	R	C	D	L	D	V	E	S	G	G	R
A. guaranitica	L	R	N	L	P	Q	Q	C	D	L	R	A	P	Q	R	C	D	L	D	V	E	S	G	G	R
A. rigonii	L	R	N	L	P	Q	Q	C	D	L	R	A	P	Q	R	C	D	L	D	V	E	S	G	G	R
A. dardani	L	R	N	L	P	Q	Q	C	D	L	R	A	P	Q	R	C	D	L	D	V	E	S	G	G	R
A. paraguariensis	L	R	N	L	P	Q	Q	C	D	L	R	A	P	Q	R	C	D	L	D	V	E	S	G	G	R
A. pintoi	L	R	N	L	P	Q	Q	C	G	L	R	S	P	Q	R	C	D	L	D	V	E	S	G	G	R
A. triseminata	L	R	N	L	P	Q	Q	C	G	F	R	A	P	Q	R	C	D	L	E	I	E	S	G	G	R
A. lutescens	L	R	N	L	P	Q	Q	C	G	L	R	A	P	Q	R	C	D	L	D	V	E	S	G	G	R
A. macedoi	L	R	N	L	P	Q	Q	C	G	L	R	A	P	Q	R	C	D	L	D	I	E	S	G	G	R

Characters	201	202	203	204
	1.20	1.20	1.20	1.20
A. ipaensis	D	R	Y	
A. ipaensis gb	D	R	Y	
A. ipaensis gb2	D	R	Y	
A. duranensis	D	R	Y	
A. duranensis gen	D	R	Y	
A. hypogaea	D	R	Y	
A. hypogaea 2.01 gb	D	R	Y	
A. hypogaea 2.02 gb	D	R	Y	
A. monticola	D	R	Y	
A. helodes	D	R	Y	
A. glandulifera	D	R	Y	
A. hoehnei	D	R	Y	
A. batizocoi	D	R	Y	
A. trinitensis	D	R	F	
A. praecox	D	R	Y	
A. palustris	D	R	Y	
A. kretschmeri	D	R	Y	
A. glabrata	D	R	Y	
A. appressipila	D	R	Y	
A. tuberosa	D	R	Y	
A. guaranitica	D	R	Y	
A. rigonii	D	R	Y	
A. dardani	D	R	Y	
A. paraguariensis	D	R	Y	
A. pintoi	D	R	Y	
A. triseminata	D	R	Y	
A. lutescens	D	R	Y	
A. macedoi	D	R	Y	

Epitope 1

Epitope 2

Epitope 3

Epitope 4

Characters	1	2	3	4	5	6	7	8	9	10	11	12	13	14	15	16	17	18	19	20	21	22	23	24	25
	1.1	1.2	1.3	1.4	1.5	1.6	1.7	1.8	1.9	1.10	1.11	1.12	1.13	1.14	1.15	1.16	1.17	1.18	1.19	1.20	1.21	1.22	1.23	1.24	1.25
A. ipaensis	?	?	?	?	?	?	?	?	?	?	?	?	?	?	?	?	?	&L&[illegible]	A	S	A	M	R	R	E
A. ipaensis gen	M	A	K	S	T	I	L	V	A	L	L	A	L	V	L	V	A	H	A	S	A	M	R	R	E
A. duranensis	?	?	?	?	?	?	?	?	?	?	?	?	?	?	&M&	V	A	H	A	S	A	M	R	R	E
A. duranensis gen	M	A	K	S	T	I	L	V	A	L	L	A	L	V	L	V	A	H	A	S	A	M	R	R	E
A. hypogaea	M	A	K	S	T	I	L	V	A	L	L	A	L	V	L	V	A	H	A	S	A	M	R	R	E
A. monticola	M	V	K	S	T	I	L	V	A	L	L	A	L	V	L	V	A	L	A	S	A	M	R	R	E
A. triseminata	M	A	K	S	T	I	L	V	A	L	L	A	L	V	L	V	A	H	A	S	A	-	R	H	E
A. guaranitica	M	A	K	S	T	I	L	V	A	L	L	A	L	V	L	V	A	H	A	S	A	M	R	R	E
A. rigonii	M	A	K	S	T	I	L	V	A	L	L	A	L	V	L	V	A	H	A	S	A	M	R	R	E
A. appressipila	M	A	K	S	T	I	L	V	A	L	L	A	L	V	L	V	A	H	A	S	A	M	R	R	E
A. paraguariensis	M	A	K	S	T	I	L	V	A	L	L	A	L	V	L	V	A	H	A	S	A	-	R	H	E
A. dardani	M	A	K	S	T	I	L	V	A	L	L	A	L	V	L	V	A	H	A	S	A	M	R	R	E
A. glandulifera	M	A	K	A	T	I	L	V	A	L	L	A	L	V	L	V	A	H	A	S	A	M	R	R	E
A. praecox	M	A	K	A	T	I	L	V	A	L	L	A	L	V	L	V	A	H	A	S	A	M	R	R	E
A. palustris	M	A	K	S	T	I	L	V	A	L	L	A	L	V	L	V	A	H	A	S	A	M	R	R	E
A. pintoi	M	A	K	S	T	I	L	V	A	L	L	A	L	V	L	V	A	H	A	S	A	M	R	R	E
A. glabrata	M	A	K	S	T	I	L	V	A	L	L	A	L	V	L	V	A	H	T	S	A	M	R	R	E
A. hoehnei	M	A	K	S	T	I	L	V	T	L	L	A	L	I	L	V	A	H	A	S	A	M	R	R	E
A. kretschmeri	M	A	K	I	T	I	L	V	A	L	L	A	L	V	L	V	A	H	A	S	A	M	R	R	E
A. macedoi	M	A	K	S	T	I	L	V	A	L	L	A	L	V	L	V	A	H	A	S	A	-	R	R	N
A. batizocoi	M	A	K	S	T	I	L	V	A	L	L	A	L	V	L	V	A	H	A	S	A	M	R	R	E
A. lutescns	M	A	K	S	T	I	V	I	A	L	L	A	L	V	L	V	A	H	A	S	A	M	R	R	E
A. helodes	M	V	K	S	T	I	L	V	A	L	L	A	L	V	L	V	A	H	A	S	A	M	R	R	E
A. trinitensis	M	A	K	A	T	I	L	V	A	L	L	A	L	V	L	V	A	H	A	S	A	M	R	R	E

Characters	26	27	28	29	30	31	32	33	34	35	36	37	38	39	40	41	42	43	44	45	46	47	48	49	50
	1.26	1.27	1.28	1.29	1.30	1.31	1.32	1.33	1.34	1.35	1.36	1.37	1.38	1.39	1.40	1.41	1.42	1.43	1.44	1.45	1.46	1.47	1.48	1.49	1.50
A. ipaensis	R	G	R	Q	G	D	S	S	S	C	E	R	Q	V	D	R	V	N	L	K	P	C	E	Q	H
A. ipaensis gen	R	G	R	Q	G	D	S	S	S	C	E	R	Q	V	D	R	V	N	L	K	P	C	E	Q	H
A. duranensis	R	G	R	Q	G	D	S	S	S	C	E	R	Q	V	D	R	V	N	L	K	P	C	E	Q	H
A. duranensis gen	R	G	R	Q	G	D	S	S	S	C	E	R	Q	V	D	R	V	N	L	K	P	C	E	Q	H
A. hypogaea	R	G	R	Q	G	D	S	S	S	C	E	R	Q	V	D	R	V	N	L	K	P	C	E	Q	H
A. monticola	R	G	R	Q	G	D	S	S	S	C	E	R	Q	V	D	R	V	N	L	K	P	C	E	Q	H
A. triseminata	W	G	Q	R	G	D	S	S	S	C	E	S	Q	I	D	R	V	N	L	K	P	C	E	Q	H
A. guaranitica	W	G	R	Q	G	D	S	S	S	C	E	R	Q	V	D	R	V	N	L	K	P	C	E	Q	H
A. rigonii	W	G	R	Q	G	D	S	S	S	C	E	R	Q	V	D	R	V	N	L	K	P	C	E	Q	H
A. appressipila	W	G	R	Q	G	D	S	S	S	C	E	R	Q	V	D	R	V	N	L	K	P	C	E	Q	H
A. paraguariensis	W	G	Q	R	G	D	S	S	S	C	E	S	Q	I	D	R	V	N	L	K	P	C	E	Q	H
A. dardani	W	G	R	Q	G	D	S	S	S	C	E	R	Q	V	D	R	V	N	L	K	P	C	E	Q	H
A. glandulifera	R	G	R	Q	G	D	S	S	S	C	E	R	Q	V	D	R	V	N	L	K	P	C	E	Q	H
A. praecox	R	G	R	Q	G	D	S	S	S	C	E	R	Q	V	D	R	V	N	L	K	P	C	E	Q	H
A. palustris	R	G	R	Q	G	D	S	S	S	C	E	R	Q	V	D	R	V	N	L	K	P	C	E	Q	H
A. pintoi	R	G	R	Q	G	D	S	S	S	C	E	R	Q	V	D	R	V	N	L	K	P	C	E	Q	H
A. glabrata	W	R	Q	H	G	D	S	S	S	C	E	R	Q	V	D	R	V	N	L	K	P	C	E	Q	H
A. hoehnei	R	G	R	Q	G	D	S	S	S	C	E	R	Q	V	D	R	V	N	L	K	P	C	E	Q	H
A. kretschmeri	R	G	R	Q	G	D	S	S	S	C	E	R	Q	V	D	R	V	N	L	K	P	C	E	Q	H
A. macedoi	W	G	H	Q	G	D	S	S	S	C	D	R	Q	V	D	R	V	N	L	K	P	C	E	Q	H
A. batizocoi	R	G	R	Q	G	D	S	S	S	C	E	R	Q	V	D	R	V	N	L	K	P	C	E	Q	H
A. lutescns	R	G	R	Q	G	D	S	S	S	C	E	R	Q	V	D	R	V	N	L	K	P	C	E	Q	H
A. helodes	R	G	R	Q	G	D	S	S	S	C	E	R	Q	V	D	R	V	N	L	K	P	C	E	Q	H
A. trinitensis	R	G	R	Q	G	D	S	S	S	C	E	R	Q	V	D	R	V	N	L	K	P	C	E	Q	H

Epitope 5

Characters	51	52	53	54	55	56	57	58	59	60	61	62	63	64	65	66	67	68	69	70	71	72	73	74	75	76	77	78	79	80	81	82	83	84	85	86	87	88	89	90	91	92	93	94	95	96	97	98	99	100
	1.51	1.52	1.53	1.54	1.55	1.56	1.57	1.58	1.59	1.60	1.61	1.62	1.63	1.64	1.65	1.66	1.67	1.68	1.69	1.70	1.71	1.72	1.73	1.74	1.75	1.76	1.77	1.78	1.79	1.80	1.81	1.82	1.83	1.84	1.85	1.86	1.87	1.88	1.89	1.90	1.91	1.92	1.93	1.94	1.95	1.96	1.97	1.98	1.99	1.10
A. ipaensis	I	M	Q	R	I	M	G	E	Q	E	Q	Y	D	S	Y	D	I	R	-	S	T	R	S	S	D	Q	Q	Q	R	C	C	D	E	L	N	E	M	E	N	T	Q	R	C	M	C	E	A	L	Q	Q
A. ipaensis gen	I	M	Q	R	I	M	G	E	Q	E	Q	Y	D	S	Y	D	I	R	-	S	T	R	S	S	D	Q	Q	Q	R	C	C	D	E	L	N	E	M	E	N	T	Q	R	C	M	C	E	A	L	Q	Q
A. duranensis	I	M	Q	R	I	M	G	E	Q	E	Q	Y	D	S	Y	D	I	R	-	S	T	R	S	S	D	Q	Q	Q	R	C	C	D	E	L	D	Q	M	E	N	T	E	R	C	M	C	E	A	L	Q	Q
A. duranensis gen	I	M	Q	R	I	M	G	E	Q	E	Q	Y	D	S	Y	D	I	R	-	S	T	R	S	S	D	Q	Q	Q	R	C	C	D	E	L	D	Q	M	E	N	T	E	R	C	M	C	E	A	L	Q	Q
A. hypogaea	I	M	Q	R	I	M	G	E	Q	E	Q	Y	D	S	Y	D	I	R	-	S	T	R	S	S	D	Q	Q	Q	R	C	C	D	E	L	N	E	M	E	N	T	Q	R	C	M	C	E	A	L	Q	Q
A. monticola	I	M	Q	R	I	M	G	E	Q	E	Q	F	D	S	Y	D	I	R	-	S	T	R	S	S	D	Q	Q	Q	R	C	C	D	E	L	N	E	M	E	N	T	Q	R	C	M	C	E	A	L	Q	Q
A. triseminata	I	M	Q	R	I	M	G	D	H	G	R	Y	D	S	Y	D	I	R	-	R	T	G	S	S	D	Q	Q	Q	R	C	C	D	E	L	N	Q	M	E	N	N	Q	R	C	M	C	E	A	L	Q	Q
A. guaranitica	I	M	Q	R	I	M	G	D	Q	E	Q	Y	D	S	Y	Y	I	R	R	S	T	R	S	S	D	Q	Q	Q	R	C	C	D	E	L	N	Q	M	E	N	T	Q	R	C	M	C	E	A	L	Q	Q
A. rigonii	I	M	Q	R	I	M	G	D	Q	E	Q	Y	D	S	Y	D	I	R	R	S	T	R	S	S	D	Q	Q	Q	R	C	C	D	E	L	N	Q	M	E	N	T	Q	R	C	M	C	E	A	L	Q	Q
A. appressipila	I	M	Q	R	I	M	G	D	Q	E	Q	Y	D	S	Y	D	I	R	R	S	T	R	S	S	D	Q	Q	Q	R	C	C	D	E	L	N	Q	M	E	N	T	Q	R	C	M	C	E	A	L	Q	Q
A. paraguariensis	I	M	Q	R	I	M	G	D	H	G	R	Y	D	S	Y	D	I	R	-	R	T	G	S	S	D	Q	Q	Q	R	C	C	D	E	L	N	Q	M	E	N	N	Q	R	C	M	C	E	A	L	Q	Q
A. dardani	I	M	Q	R	I	M	G	D	Q	E	Q	Y	D	S	Y	D	I	R	R	S	T	R	S	S	D	Q	Q	Q	R	C	C	D	E	L	N	Q	M	E	N	T	Q	R	C	M	C	E	A	L	Q	Q
A. glandulifera	I	M	Q	R	I	M	G	E	Q	E	Q	Y	D	S	Y	D	I	R	-	S	T	R	S	S	D	Q	Q	Q	R	C	C	D	E	L	N	E	M	E	N	T	Q	R	C	M	C	E	A	L	Q	Q
A. praecox	I	M	Q	R	I	M	G	E	Q	E	Q	Y	D	S	Y	D	I	R	-	S	T	R	S	S	D	Q	Q	Q	R	C	C	D	E	L	D	Q	M	E	N	T	Q	R	C	M	C	E	A	L	Q	Q
A. palustris	I	M	Q	R	I	M	G	E	Q	E	Q	Y	D	S	Y	D	I	R	-	S	T	R	S	S	D	Q	Q	Q	R	C	C	D	E	L	D	Q	M	E	N	T	Q	R	C	M	C	E	A	L	Q	Q
A. pintoi	I	M	Q	R	I	M	G	E	Q	E	Q	Y	D	S	Y	D	I	R	-	S	T	R	S	S	D	Q	Q	Q	R	C	C	D	E	L	D	Q	M	E	N	T	E	R	C	M	C	E	A	L	Q	Q
A. glabrata	I	M	Q	R	I	M	G	D	Q	D	Q	Y	D	S	Y	D	I	R	-	S	T	R	S	S	D	Q	Q	Q	R	C	C	D	E	L	N	Q	M	E	N	T	Q	R	C	M	C	E	A	L	Q	Q
A. hoehnei	I	M	Q	R	I	M	G	E	Q	E	Q	Y	D	S	Y	D	I	R	-	S	T	R	S	S	D	Q	Q	Q	R	C	C	D	E	L	D	Q	M	E	N	T	E	R	C	M	C	E	A	L	Q	Q
A. kretschmeri	I	M	Q	R	I	M	G	E	Q	E	Q	Y	D	S	Y	D	I	R	-	S	T	R	S	S	D	Q	Q	Q	R	C	C	D	E	L	D	Q	M	E	N	T	E	R	C	M	C	E	A	L	Q	Q
A. macedoi	I	M	Q	R	I	M	G	D	Q	D	Q	Y	D	S	Y	Y	I	R	-	R	S	G	S	S	D	D	Q	Q	R	C	C	D	E	L	N	Q	M	E	N	T	Q	R	C	M	C	E	A	L	Q	Q
A. batizocoi	I	M	Q	R	I	M	G	E	Q	E	Q	Y	D	S	Y	D	I	R	-	S	T	R	S	S	D	Q	Q	Q	R	C	C	D	E	L	D	Q	M	E	N	T	Q	R	C	M	C	E	A	L	Q	Q
A. lutescens	I	M	Q	R	I	M	G	E	Q	E	Q	Y	D	S	Y	D	I	R	-	S	T	R	S	S	D	Q	Q	Q	R	C	C	D	E	L	D	Q	M	E	N	T	E	R	C	M	C	E	A	L	Q	Q
A. helodes	I	M	Q	R	I	M	G	E	Q	E	Q	Y	D	S	Y	D	I	R	-	S	T	R	S	S	D	Q	Q	Q	R	C	C	D	E	L	N	E	M	E	N	T	Q	R	C	M	C	E	A	L	Q	Q
A. trinitensis	I	M	Q	R	I	M	G	E	Q	E	Q	Y	D	S	Y	D	I	R	-	S	T	R	S	S	D	Q	Q	Q	R	C	C	D	E	L	N	E	M	E	N	T	Q	R	C	M	C	E	A	L	Q	Q

Epitope 8 (characters 108–117) | Epitope 9 (characters 119–128) | Epitope 10 (characters 135–141)

Characters	101	102	103	104	105	106	107	108	109	110	111	112	113	114	115	116	117	118	119	120	121	122	123	124
	1.10	1.10	1.10	1.10	1.10	1.10	1.10	1.10	1.10	1.11	1.11	1.11	1.11	1.11	1.11	1.11	1.11	1.11	1.11	1.12	1.12	1.12	1.12	1.12
A. ipaensis	I	M	E	N	Q	C	D	R	L	Q	D	R	Q	M	V	Q	Q	F	K	R	E	L	M	N
A. ipaensis gen	I	M	E	N	Q	C	D	R	L	Q	D	R	Q	M	V	Q	Q	F	K	R	E	L	M	N
A. duranensis	I	M	E	N	Q	C	D	R	L	Q	D	R	Q	M	V	Q	Q	F	K	R	E	L	M	N
A. duranensis gen	I	M	E	N	Q	C	D	R	L	Q	D	R	Q	M	V	Q	Q	F	K	R	E	L	M	N
A. hypogaea	I	M	E	N	Q	C	D	R	L	Q	D	R	Q	M	V	Q	Q	F	K	R	E	L	M	N
A. monticola	I	M	E	N	Q	C	D	R	L	Q	D	R	Q	M	V	Q	Q	F	K	R	E	L	M	N
A. triseminata	I	M	E	N	Q	C	D	R	L	Q	D	R	Q	M	V	Q	Q	F	K	R	E	L	M	N
A. guaranitica	I	M	E	N	Q	C	D	R	L	Q	D	R	Q	M	V	Q	Q	F	Q	R	E	L	M	N
A. rigonii	I	M	E	N	Q	C	D	R	L	Q	D	R	Q	M	V	Q	Q	F	K	R	E	L	M	N
A. appressipila	I	M	E	N	Q	C	D	R	L	Q	D	R	Q	M	V	Q	Q	F	K	R	E	L	M	N
A. paraguariensis	I	M	E	N	Q	C	D	R	L	Q	D	R	Q	M	V	Q	Q	F	K	R	E	L	M	N
A. dardani	I	M	E	N	Q	C	D	R	L	Q	D	R	Q	M	V	Q	Q	F	K	R	E	L	M	N
A. glandulifera	I	M	E	N	Q	C	D	R	L	Q	D	R	Q	M	V	Q	Q	F	K	R	E	L	M	N
A. praecox	I	M	E	N	Q	C	D	R	L	Q	D	R	Q	M	V	Q	Q	F	K	R	E	L	M	N
A. palustris	I	M	E	N	Q	C	D	R	L	Q	D	R	Q	M	V	Q	Q	F	K	R	E	L	M	N
A. pintoi	I	M	E	N	Q	C	D	R	L	Q	D	R	Q	M	V	Q	Q	F	K	R	E	L	M	N
A. glabrata	I	M	E	N	Q	C	D	R	L	Q	D	R	Q	M	V	Q	Q	F	K	R	E	L	M	N
A. hoehnei	I	M	E	N	Q	C	D	R	L	Q	D	R	Q	M	V	Q	Q	F	K	R	E	L	M	N
A. kretschmeri	I	M	E	N	Q	C	D	R	L	Q	D	R	Q	M	V	Q	Q	F	K	R	E	L	M	N
A. macedoi	I	M	E	N	Q	C	D	R	L	Q	D	R	Q	M	V	Q	Q	F	K	R	E	L	M	N
A. batizocoi	I	M	E	N	Q	C	D	R	L	Q	D	R	Q	M	V	Q	Q	F	K	R	E	L	M	N
A. lutescns	I	M	E	N	Q	C	D	R	L	Q	D	R	Q	M	V	Q	Q	F	K	R	E	L	M	N
A. helodes	I	M	E	N	Q	C	D	R	L	Q	D	R	Q	M	V	Q	Q	F	K	R	E	L	M	N
A. trinitensis	I	M	E	N	Q	C	D	R	L	Q	D	R	Q	M	V	Q	Q	F	K	R	E	L	M	N

Characters	125	126	127	128	129	130	131	132	133	134	135	136	137	138	139	140	141	142	143	144	145	146	147
	1.12	1.12	1.12	1.12	1.12	1.13	1.13	1.13	1.13	1.13	1.13	1.13	1.13	1.13	1.13	1.14	1.14	1.14	1.14	1.14	1.14	1.14	1.14
A. ipaensis	L	P	Q	Q	C	N	F	R	A	P	Q	R	C	D	L	D	V	S	G	G	R	C	
A. ipaensis gen	L	P	Q	Q	C	N	F	R	A	P	Q	R	C	D	L	D	V	S	G	G	R	C	
A. duranensis	L	P	Q	Q	C	N	F	R	A	P	Q	R	C	D	L	D	V	S	G	G	R	C	
A. duranensis gen	L	P	Q	Q	C	N	F	R	A	P	Q	R	C	D	L	D	V	S	G	G	R	C	
A. hypogaea	L	P	Q	Q	C	N	F	R	A	P	Q	R	C	D	L	D	V	S	G	G	R	C	
A. monticola	L	P	Q	Q	C	N	F	R	A	P	Q	R	C	D	L	D	V	S	G	G	R	C	
A. triseminata	L	P	Q	Q	C	Y	F	R	A	P	M	H	C	D	L	D	V	S	G	G	R	C	
A. guaranitica	L	P	Q	Q	C	N	F	R	A	P	Q	L	C	D	L	D	V	S	G	G	R	C	
A. rigonii	L	P	Q	Q	C	N	F	R	A	P	Q	R	C	D	L	D	V	S	G	G	R	C	
A. appressipila	L	P	Q	Q	C	N	F	R	A	P	Q	R	C	D	L	D	V	S	G	G	R	C	
A. paraguariensis	L	P	Q	Q	C	Y	F	R	A	P	M	H	C	D	L	D	V	S	G	G	R	C	
A. dardani	L	P	Q	Q	C	N	F	R	A	P	Q	R	C	D	L	D	V	S	G	G	R	C	
A. glandulifera	L	P	Q	Q	C	N	F	R	A	P	Q	R	C	D	L	D	V	S	G	G	R	C	
A. praecox	L	P	Q	Q	C	N	F	R	A	P	Q	R	C	D	L	D	V	S	G	G	R	C	
A. palustris	L	P	Q	Q	C	N	F	R	A	P	Q	R	C	D	L	D	V	S	G	G	R	C	
A. pintoi	L	P	Q	Q	C	N	F	R	A	P	Q	R	C	D	L	D	V	S	G	G	R	C	
A. glabrata	L	P	Q	Q	C	Y	F	R	A	P	Q	S	C	D	L	D	V	S	G	G	R	C	
A. hoehnei	L	P	Q	Q	C	N	F	R	A	P	Q	R	C	D	L	D	V	S	G	G	R	C	
A. kretschmeri	L	P	Q	Q	C	N	F	R	A	P	Q	R	C	D	L	D	M	S	G	G	R	C	
A. macedoi	L	P	Q	Q	C	Y	F	R	T	P	Q	H	C	D	L	D	V	S	G	G	R	C	
A. batizocoi	L	P	Q	Q	C	N	F	R	A	P	Q	R	C	D	L	D	V	S	G	G	R	C	
A. lutescns	L	P	Q	Q	C	N	F	R	A	P	Q	R	C	D	L	D	M	S	G	G	R	C	
A. helodes	L	P	Q	Q	C	N	F	R	A	P	Q	R	C	D	L	D	V	S	G	G	R	C	
A. trinitensis	L	P	Q	Q	C	N	F	R	A	P	Q	H	C	D	L	D	V	S	G	G	R	C	
