# Supplementary material for: Molecular evolution of genes encoding allergen proteins in the peanuts genus Arachis: Structural and functional implications
Source: PLoS One. 2019 Nov 1;14(11):e0222440. doi: 10.1371/journal.pone.0222440 (PMC6824556; doi:10.1371/journal.pone.0222440)

Supplementary fig S2. Standard deviations calculated for deduced amino acids sequences of the species used in this study. The amino acid symbols refer to: A: Alanine, C: Cysteine, D: Aspartic acid, E: Glutamic acid, F: Phenylalanine, G: Glycine, H: Histidine, I: Isoleucine, K: Lysine, L: Leucine, M: Methionine, N: Asparagine, O: Proline, Q: Glutamine, R: Arginine, S: Serine, T: Threonine, V: Valine, W: Tryptophan, Y: Tyrosine

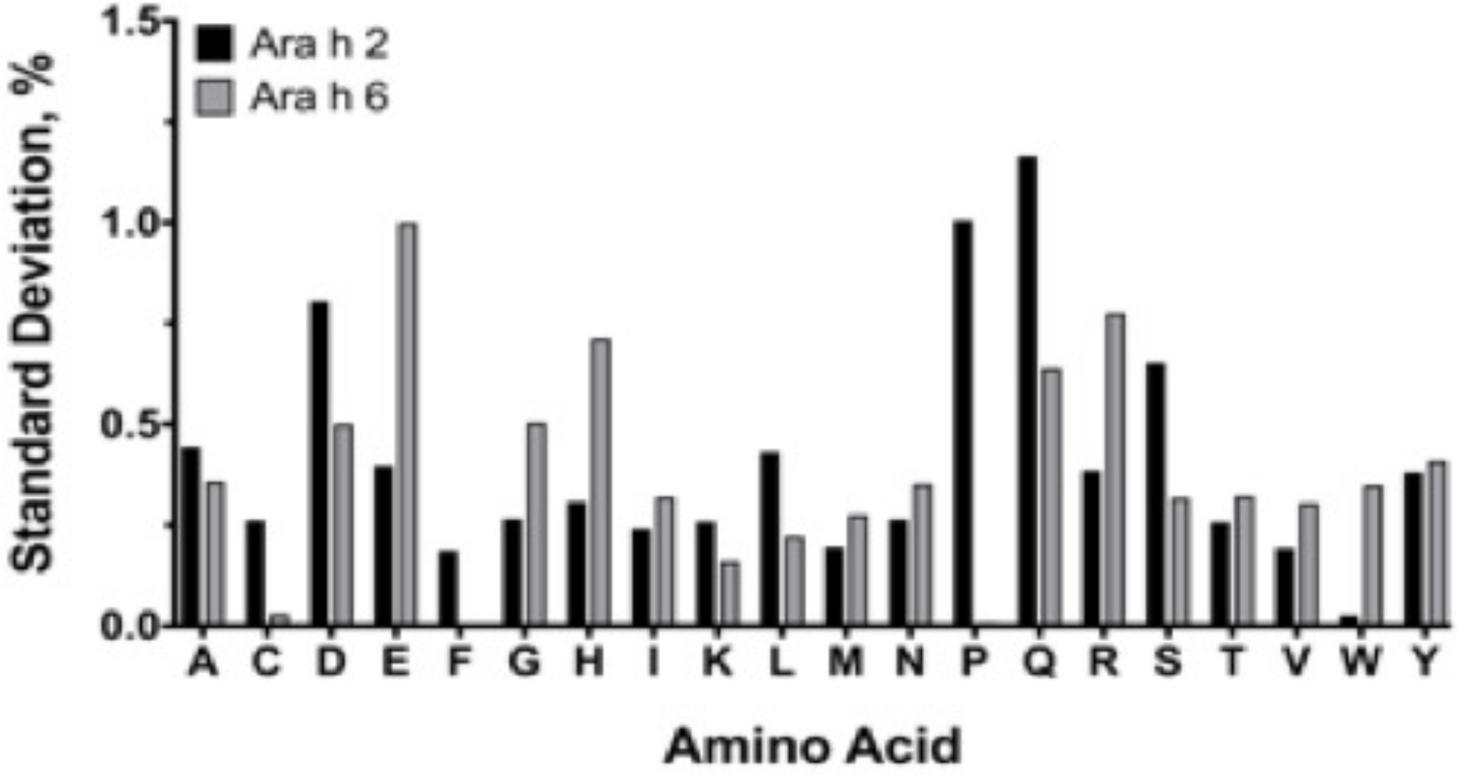

Supplement: S2 Fig — The amino acid symbols refer to: A: Alanine, C: Cysteine, D: Aspartic acid, E: Glutamic acid, F: Phenylalanine, G: Glycine, H: Histidine, I: Isoleucine, K: Lysine, L: Leucine, M: Methionine, N: Asparagine, P: Proline, Q: Glutamine, R: Arginine, S: Serine, T: Threonine, V: Valine, W: Tryptophan, Y: Tyrosine. (PDF) [file pone.0222440.s005.pdf]
