# Supplementary material for: Molecular evolution of genes encoding allergen proteins in the peanuts genus Arachis: Structural and functional implications
Source: PLoS One. 2019 Nov 1;14(11):e0222440. doi: 10.1371/journal.pone.0222440 (PMC6824556; doi:10.1371/journal.pone.0222440)
Supplement: S3 Fig — Detailed descriptions of structures are noted. (PDF) [file pone.0222440.s006.pdf]

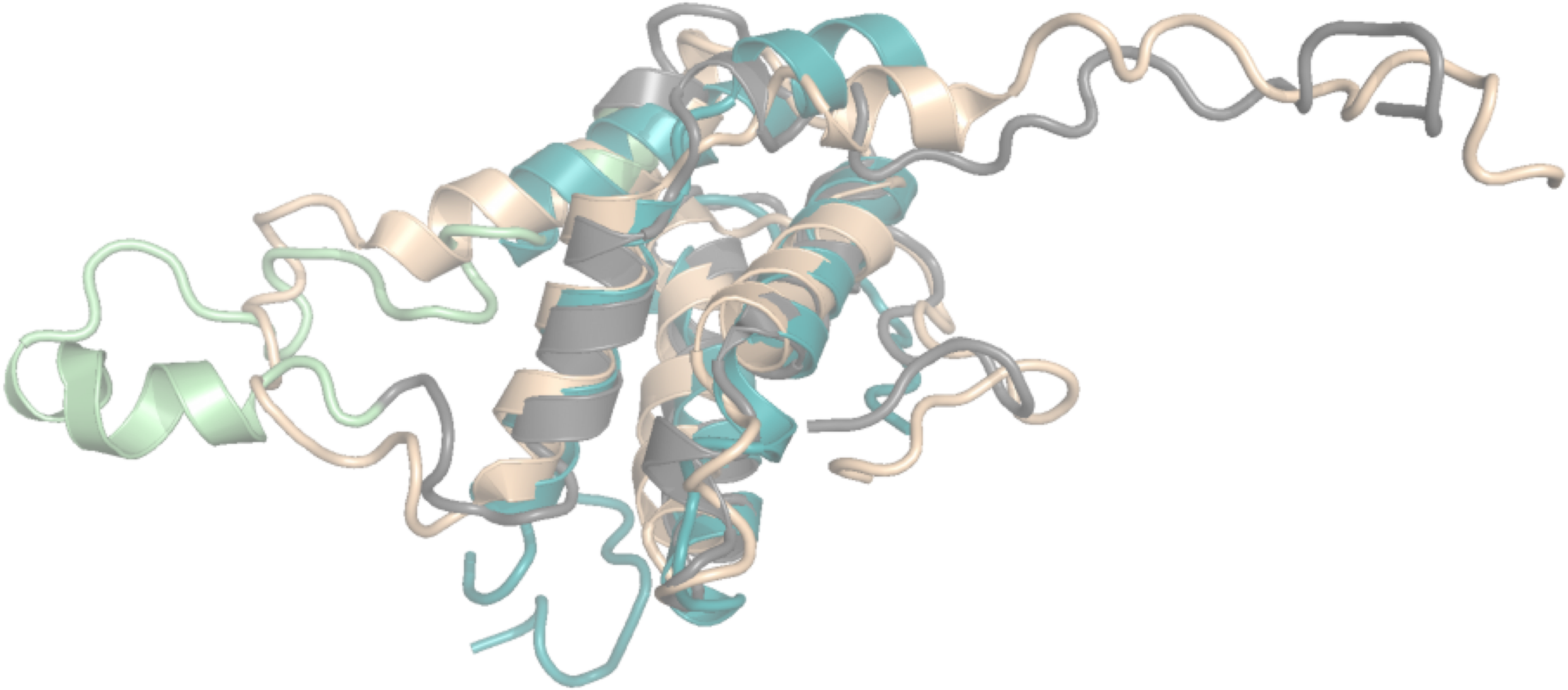

Figure S3A. Structural overlay of *A. hypogaea* 2.02 Ara h 2 homology model (dark grey), Ara h 2 NMR structure (PDB ID: 3OB4) with MBP removed for clarity (deep teal), and Ara h 6 NMR structure (PDB ID: 1W2Q , tan). Structures are shown as cartoon. The loop region that contains epitope 6, 7, and 7b in the homology model is shown as light green to denote it being missing from the Ara h 2 NMR structure.

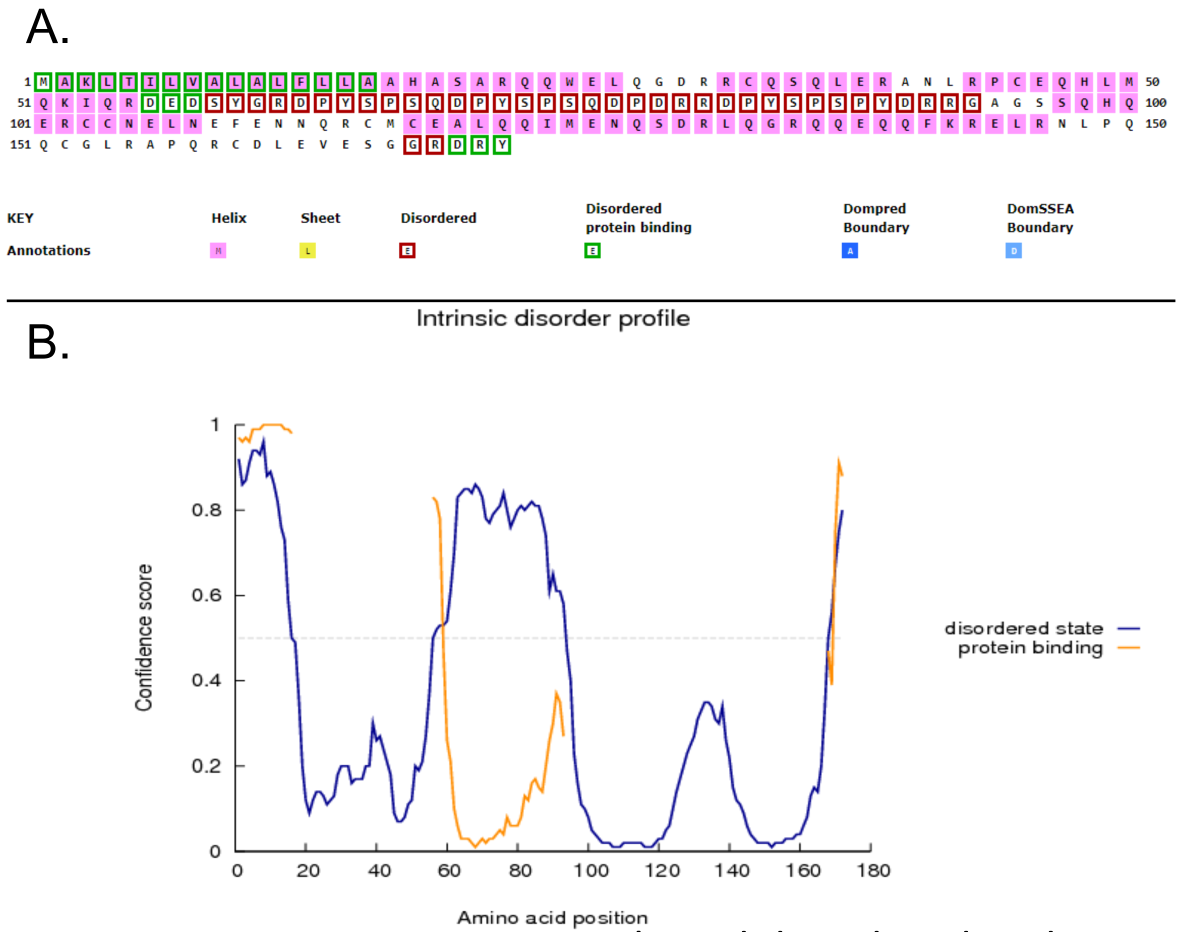

Figure S3B. PSIPRED sequence based disordered and disordered protein binding results for *A. ipaensis* Ara h 2. (A) shows the residues predicted to be disordered, involved in a helix, and to bind other proteins (e.g. IgE). (B) highlights the intrinsic disordered profile per amino acid position that align with key epitope regions of Ara h 2. , See figure legends for coloring descriptions.

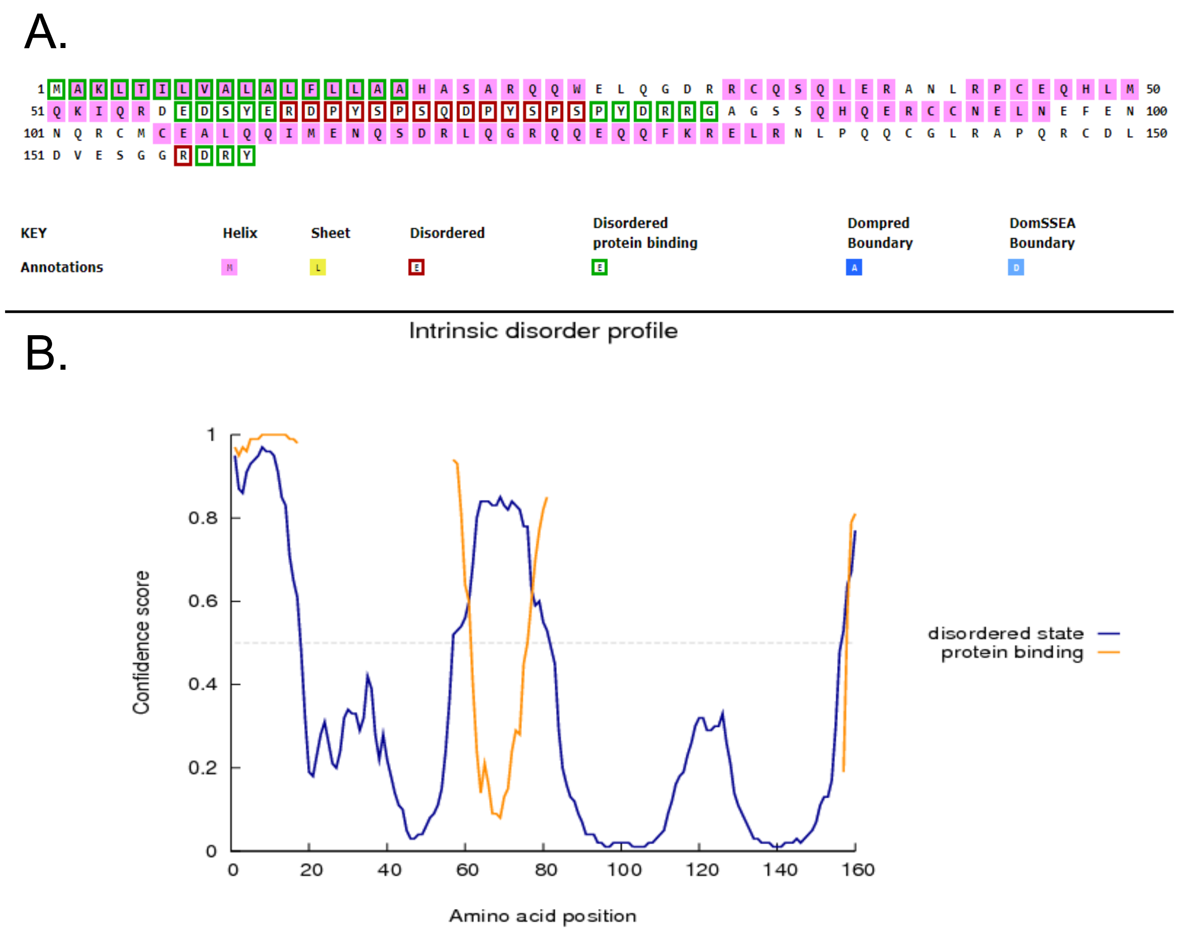

Figure S3C. PSIPRED sequence based disordered and disordered protein binding results for *A. duranensis* Ara h 2. (A) shows the residues predicted to be disordered, involved in a helix, and to bind other proteins (e.g. IgE). (B) highlights the intrinsic disordered profile per amino acid position that align with key epitope regions of Ara h 2. , See figure legends for coloring descriptions.

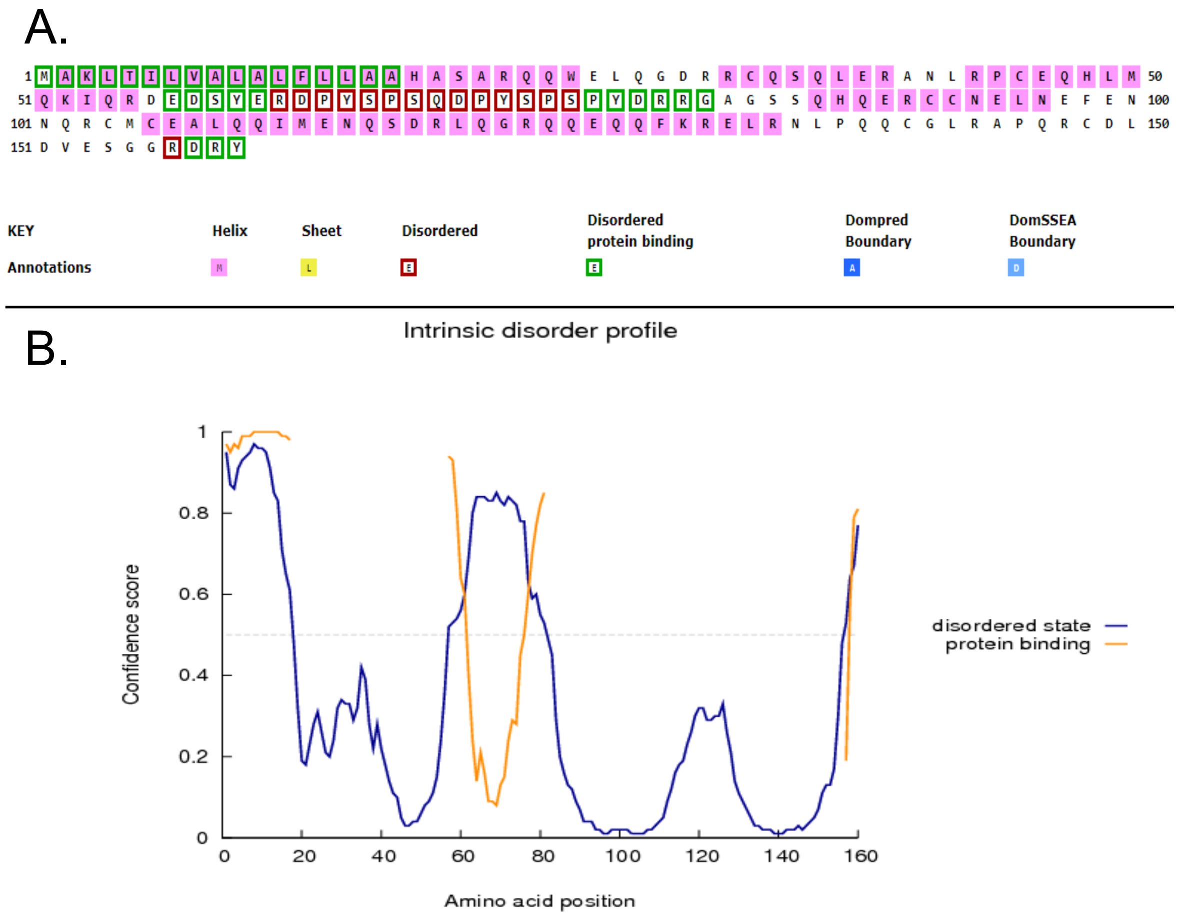

Figure S3D. PSIPRED sequence based disordered and disordered protein binding results for *A. hypogaea* Ara h 2. (A) shows the residues predicted to be disordered, involved in a helix, and to bind other proteins (e.g. IgE). (B) highlights the intrinsic disordered profile per amino acid position that align with key epitope regions of Ara h 2. , See figure legends for coloring descriptions.

A.

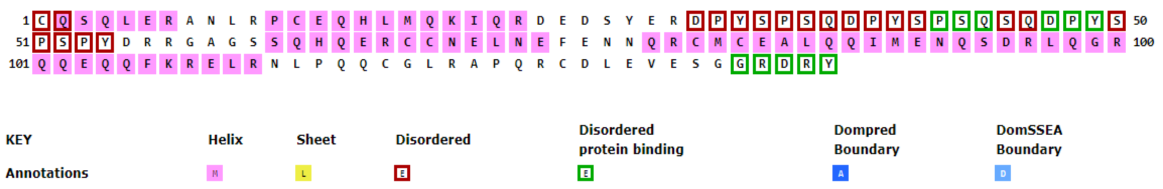

B.

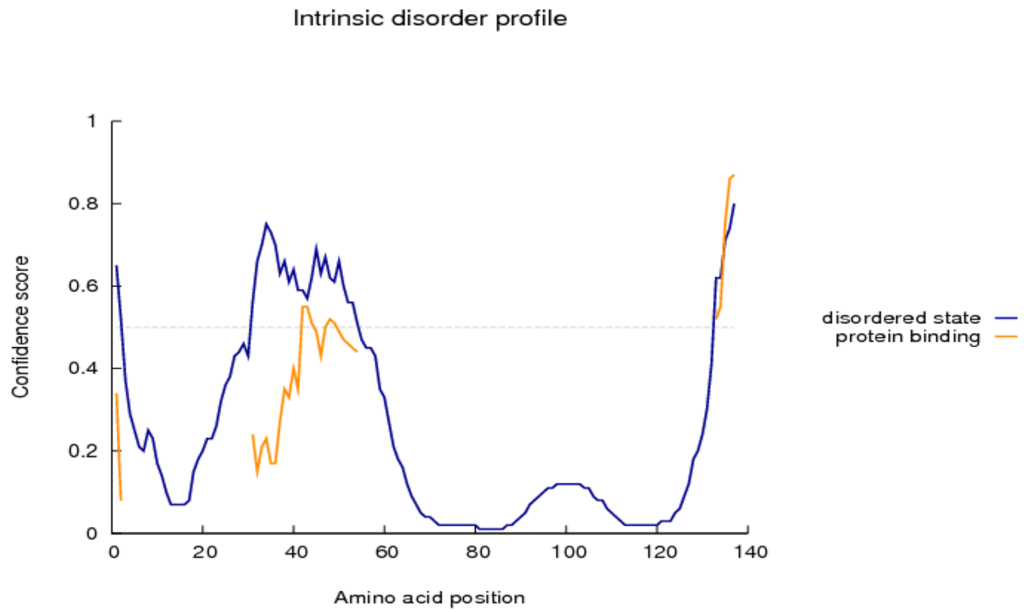

Figure S3E. PSIPRED sequence based disordered and disordered protein binding results for *A. monticola* Ara h 2. (A) shows the residues predicted to be disordered, involved in a helix, and to bind other proteins (e.g. IgE). (B) highlights the intrinsic disordered profile per amino acid position that align with key epitope regions of Ara h 2. See figure legends for coloring descriptions.

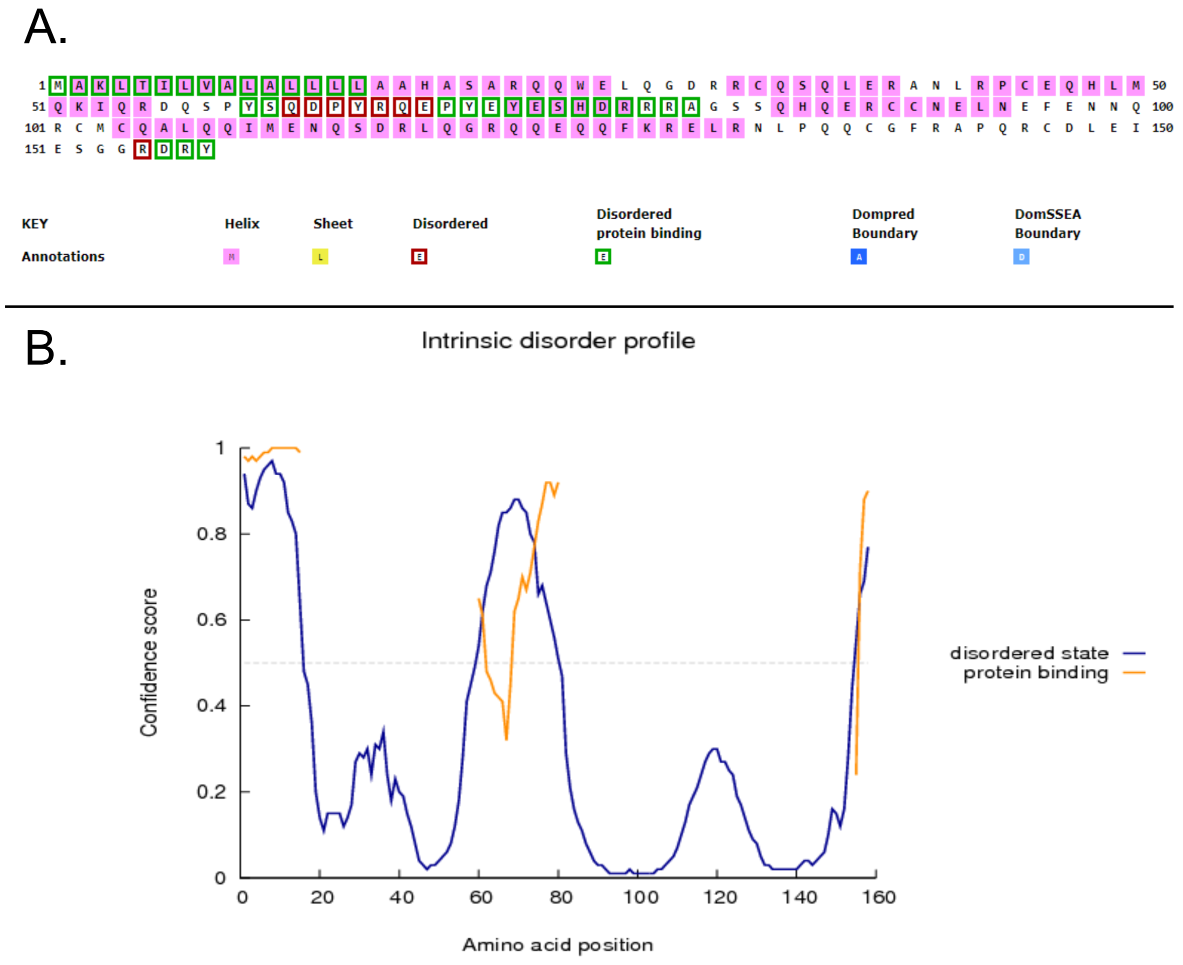

Figure S3F. PSIPRED sequence based disordered and disordered protein binding results for *A. triseminiata* Ara h 2. (A) shows the residues predicted to be disordered, involved in a helix, and to bind other proteins (e.g. IgE). (B) highlights the intrinsic disordered profile per amino acid position that align with key epitope regions of Ara h 2. See figure legends for coloring descriptions.

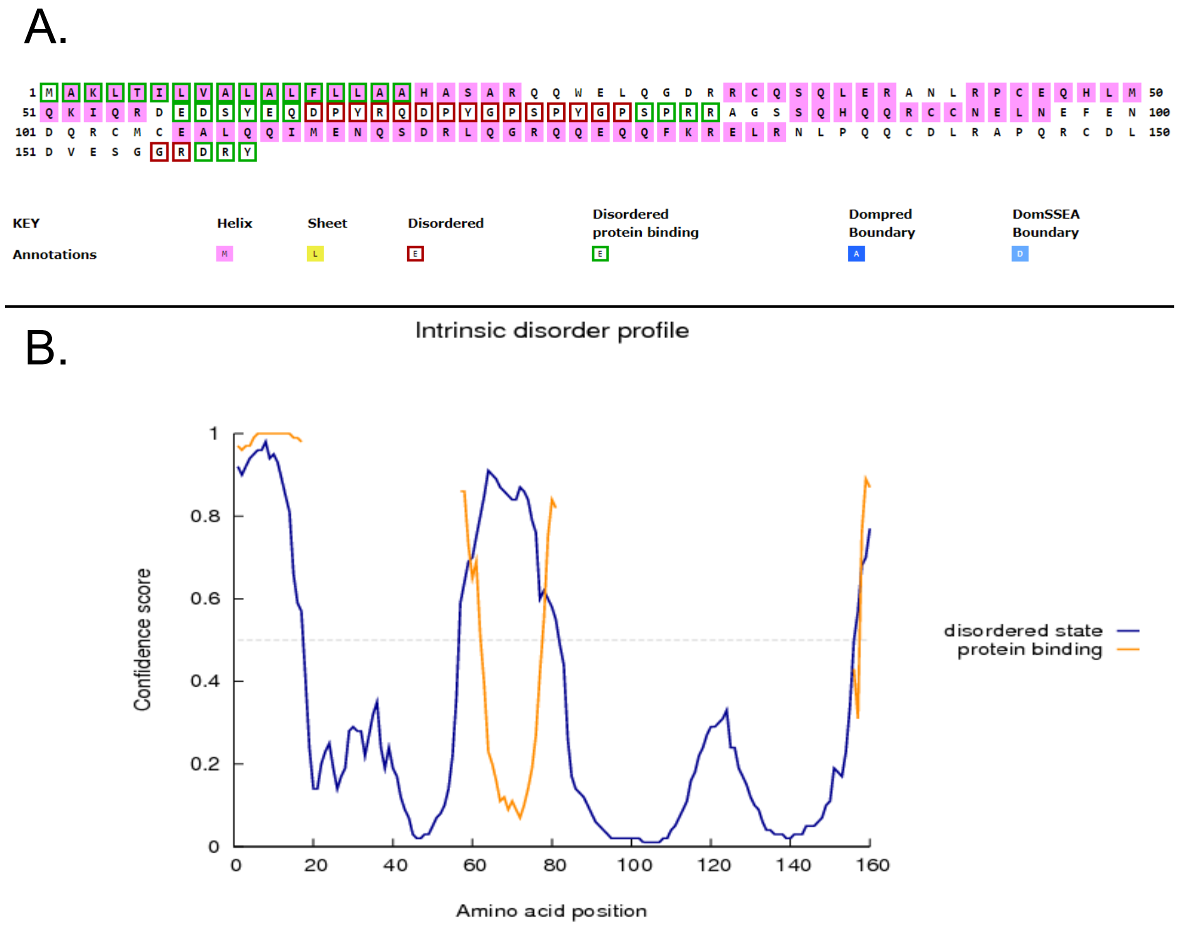

Figure S3G. PSIPRED sequence based disordered and disordered protein binding results for *A. guaranitica* Ara h 2. (A) shows the residues predicted to be disordered, involved in a helix, and to bind other proteins (e.g. IgE). (B) highlights the intrinsic disordered profile per amino acid position that align with key epitope regions of Ara h 2. See figure legends for coloring descriptions.

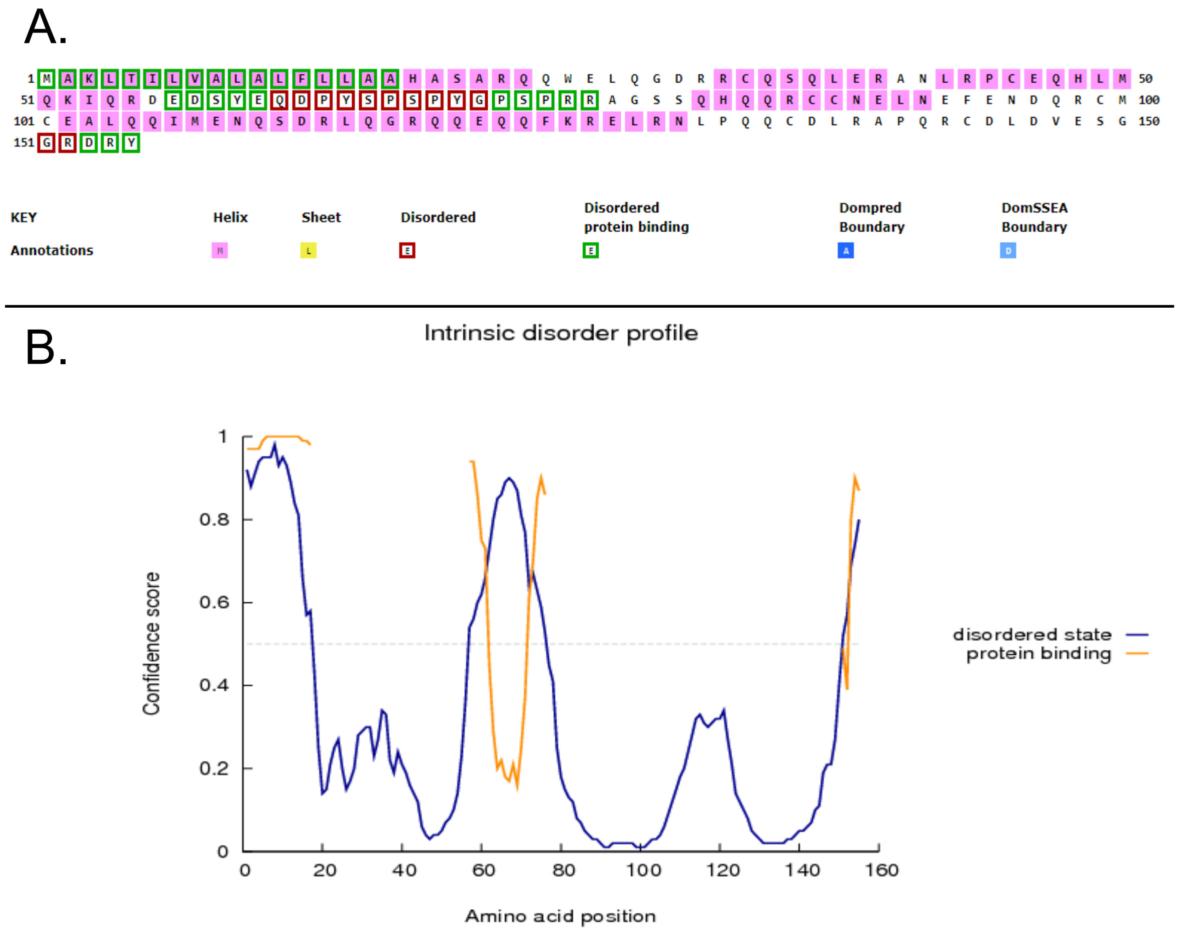

Figure S3H. PSIPRED sequence based disordered and disordered protein binding results for *A. rigonii* Ara h 2. (A) shows the residues predicted to be disordered, involved in a helix, and to bind other proteins (e.g. IgE). (B) highlights the intrinsic disordered profile per amino acid position that align with key epitope regions of Ara h 2. See figure legends for coloring descriptions.

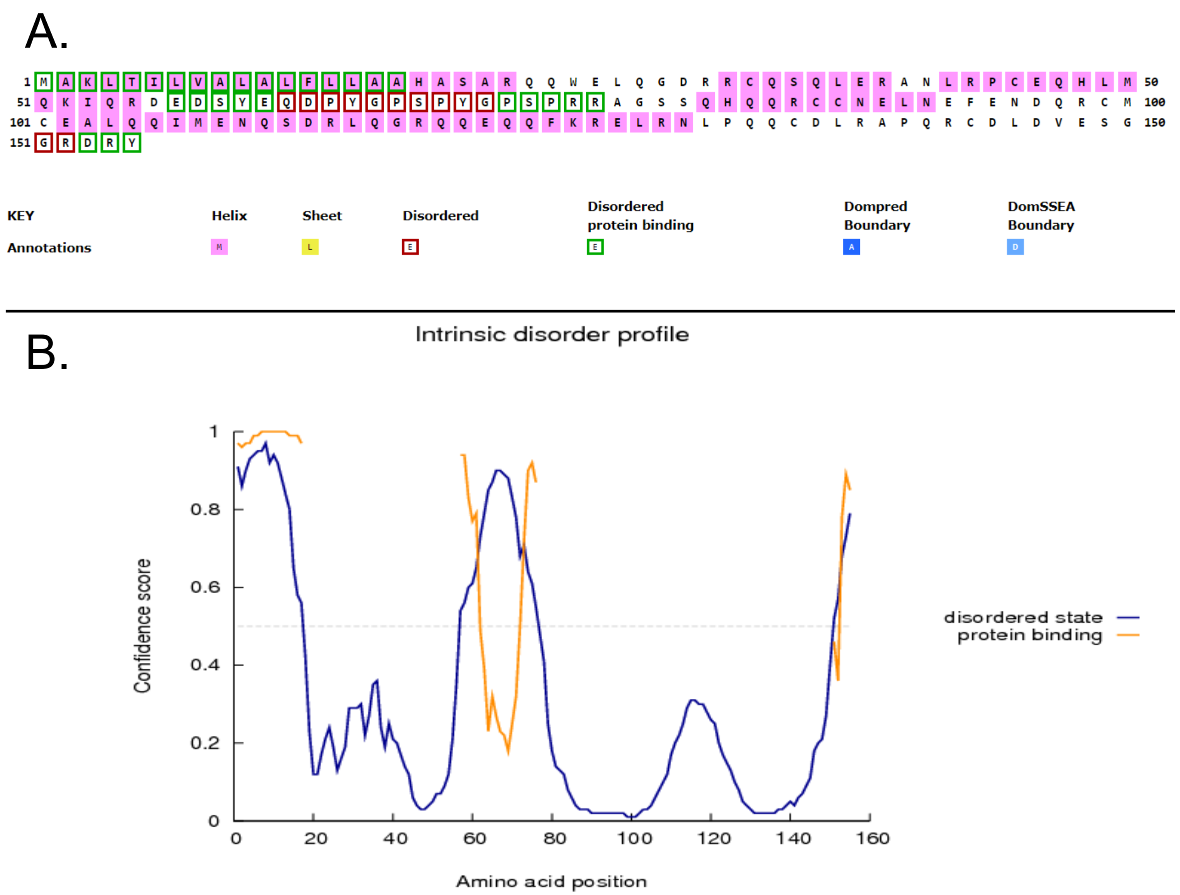

Figure S3I. PSIPRED sequence based disordered and disordered protein binding results for *A. appressipila* Ara h 2. (A) shows the residues predicted to be disordered, involved in a helix, and to bind other proteins (e.g. IgE). (B) highlights the intrinsic disordered profile per amino acid position that align with key epitope regions of Ara h 2. See figure legends for coloring descriptions.

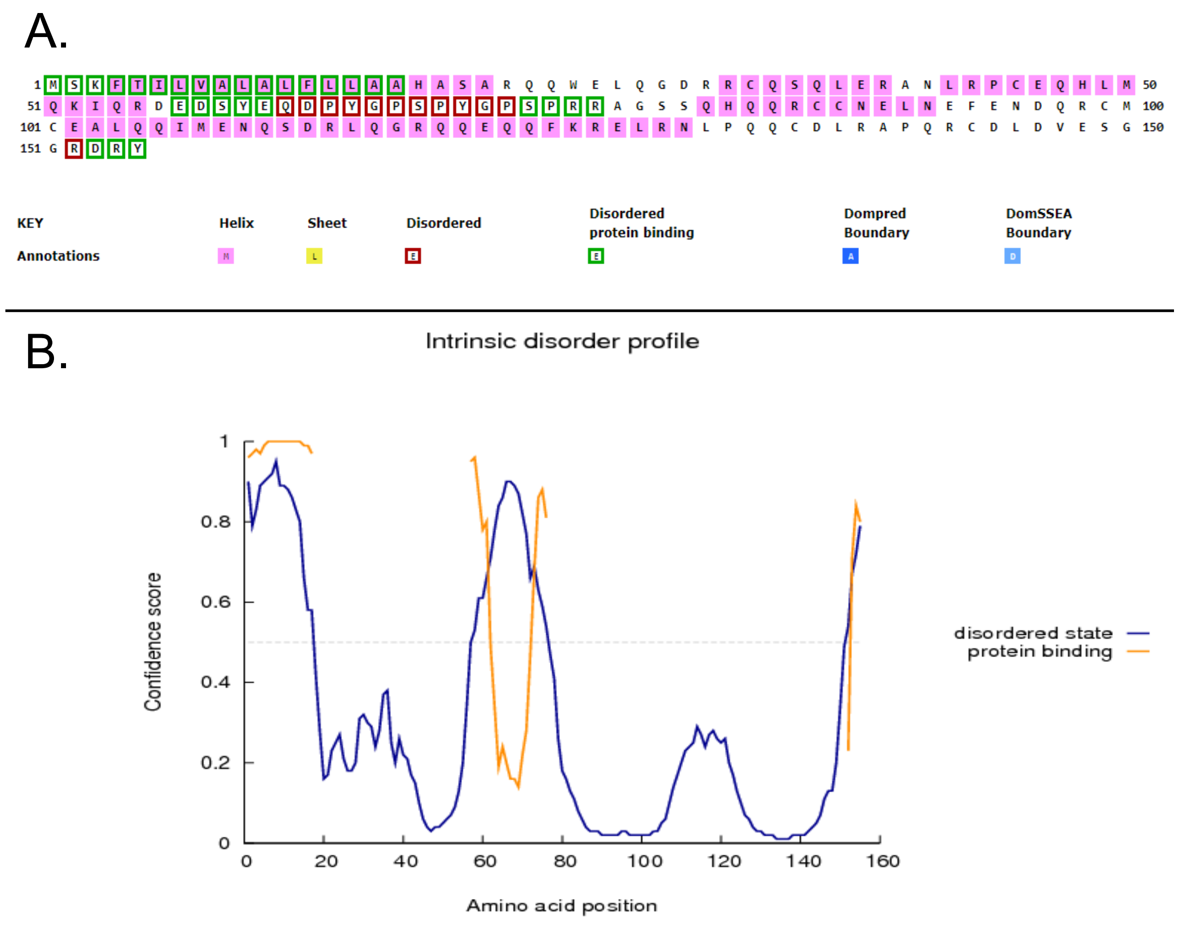

Figure S3J. PSIPRED sequence based disordered and disordered protein binding results for *A. paraguariensis* Ara h 2. (A) shows the residues predicted to be disordered, involved in a helix, and to bind other proteins (e.g. IgE). (B) highlights the intrinsic disordered profile per amino acid position that align with key epitope regions of Ara h 2. See figure legends for coloring descriptions.

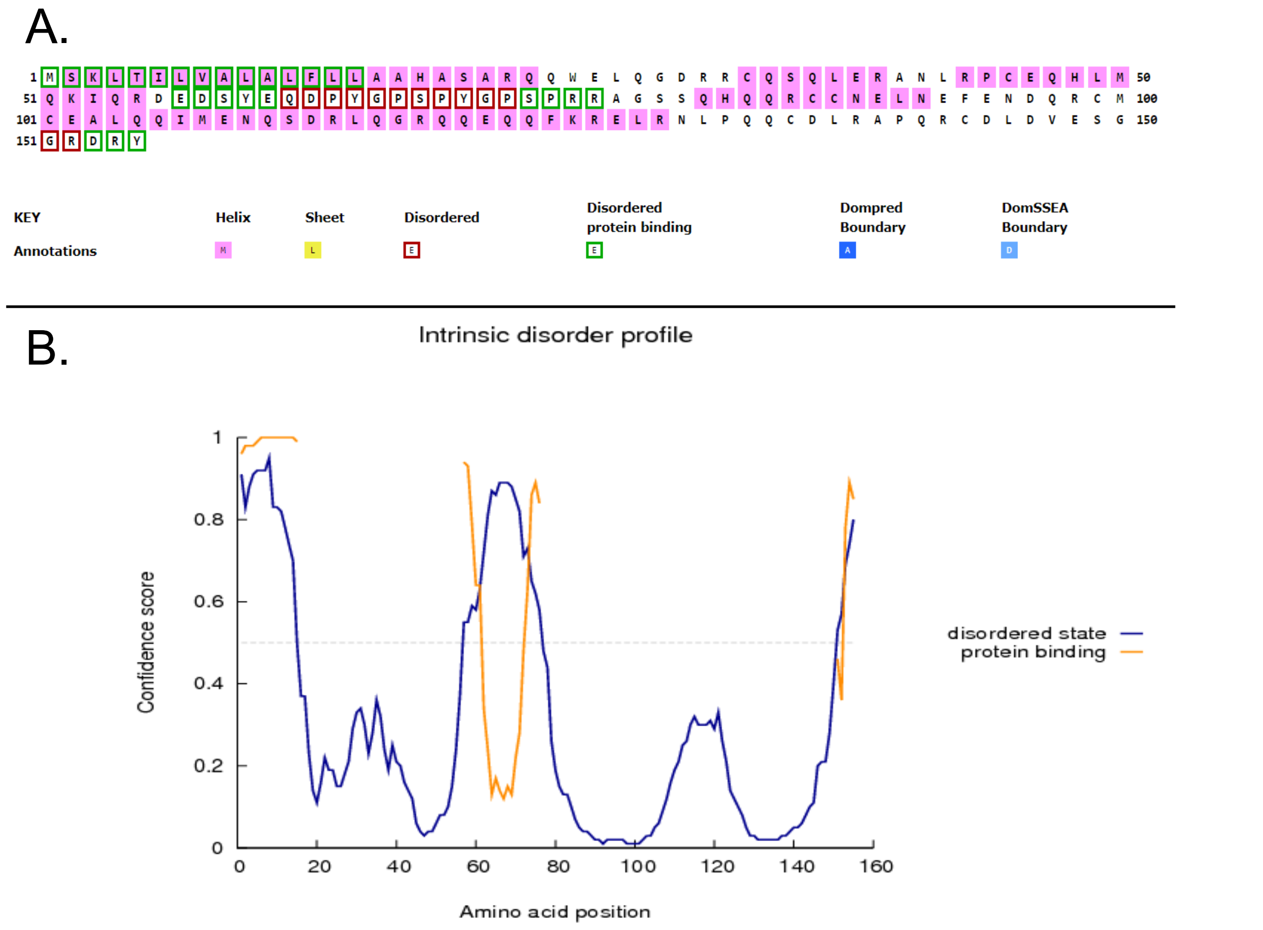

Figure S3J. PSIPRED sequence based disordered and disordered protein binding results for *A. dardani* Ara h 2. (A) shows the residues predicted to be disordered, involved in a helix, and to bind other proteins (e.g. IgE). (B) highlights the intrinsic disordered profile per amino acid position that align with key epitope regions of Ara h 2. See figure legends for coloring descriptions.

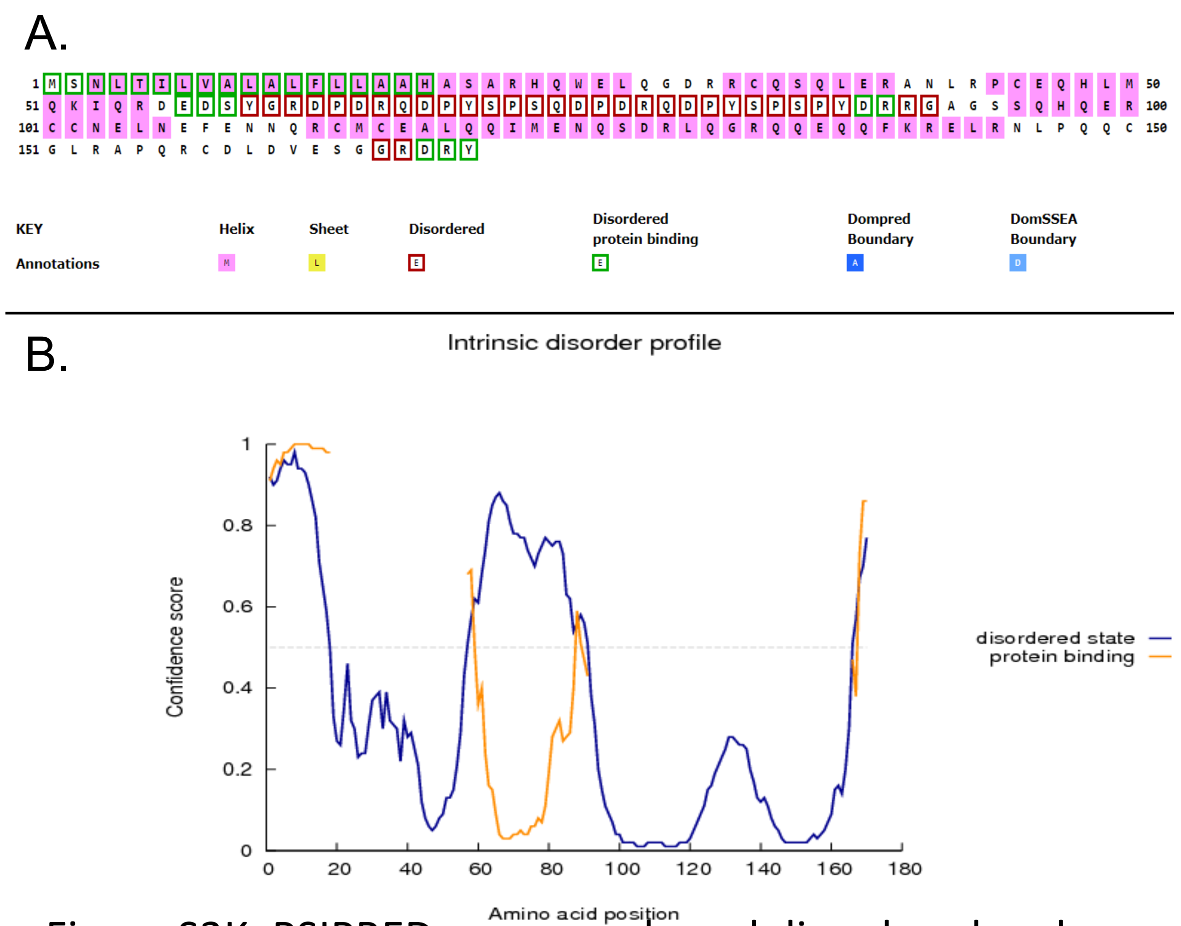

Figure S3K. PSIPRED sequence based disordered and disordered protein binding results for *A. glandulifera* Ara h 2. (A) shows the residues predicted to be disordered, involved in a helix, and to bind other proteins (e.g. IgE). (B) highlights the intrinsic disordered profile per amino acid position that align with key epitope regions of Ara h 2. See figure legends for coloring descriptions.

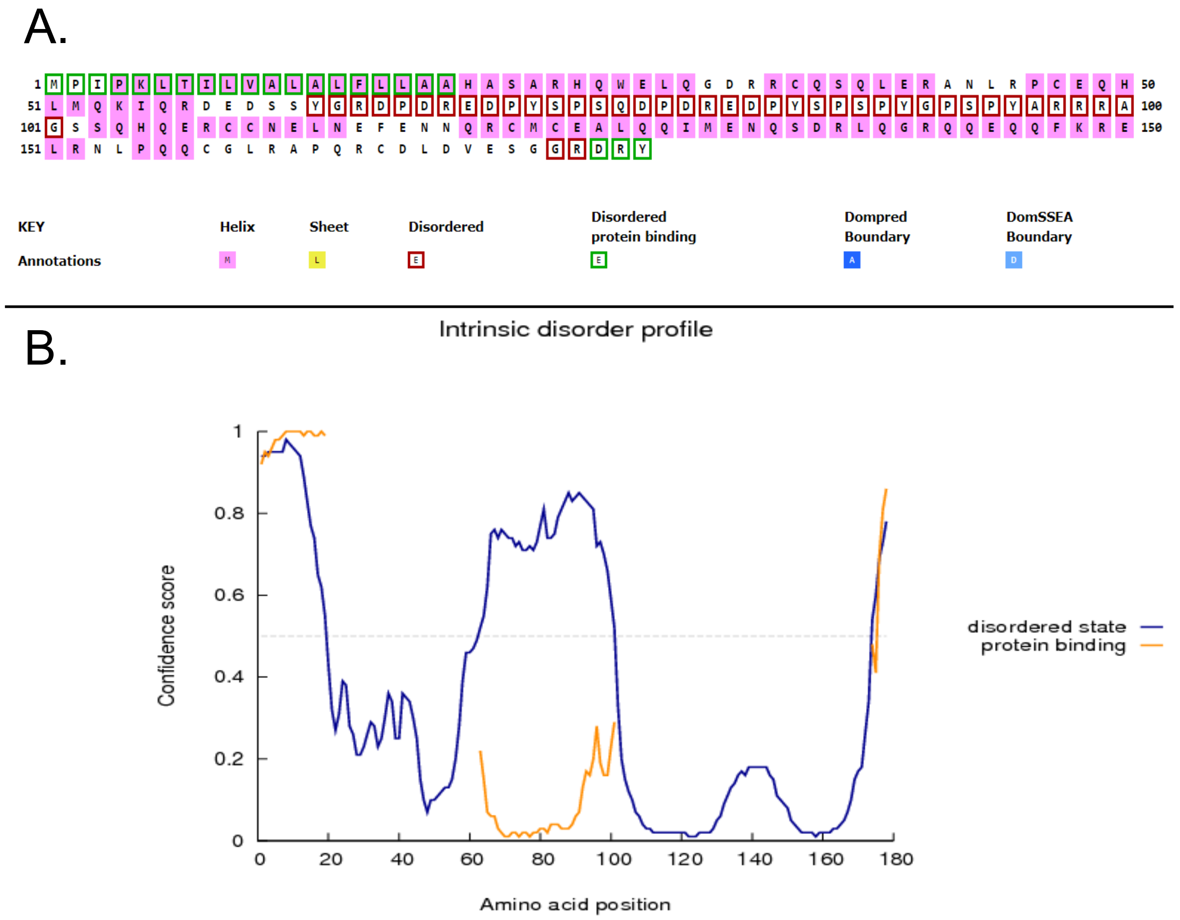

Figure S3L. PSIPRED sequence based disordered and disordered protein binding results for *A. praecox* Ara h 2. (A) shows the residues predicted to be disordered, involved in a helix, and to bind other proteins (e.g. IgE). (B) highlights the intrinsic disordered profile per amino acid position that align with key epitope regions of Ara h 2. See figure legends for coloring descriptions.

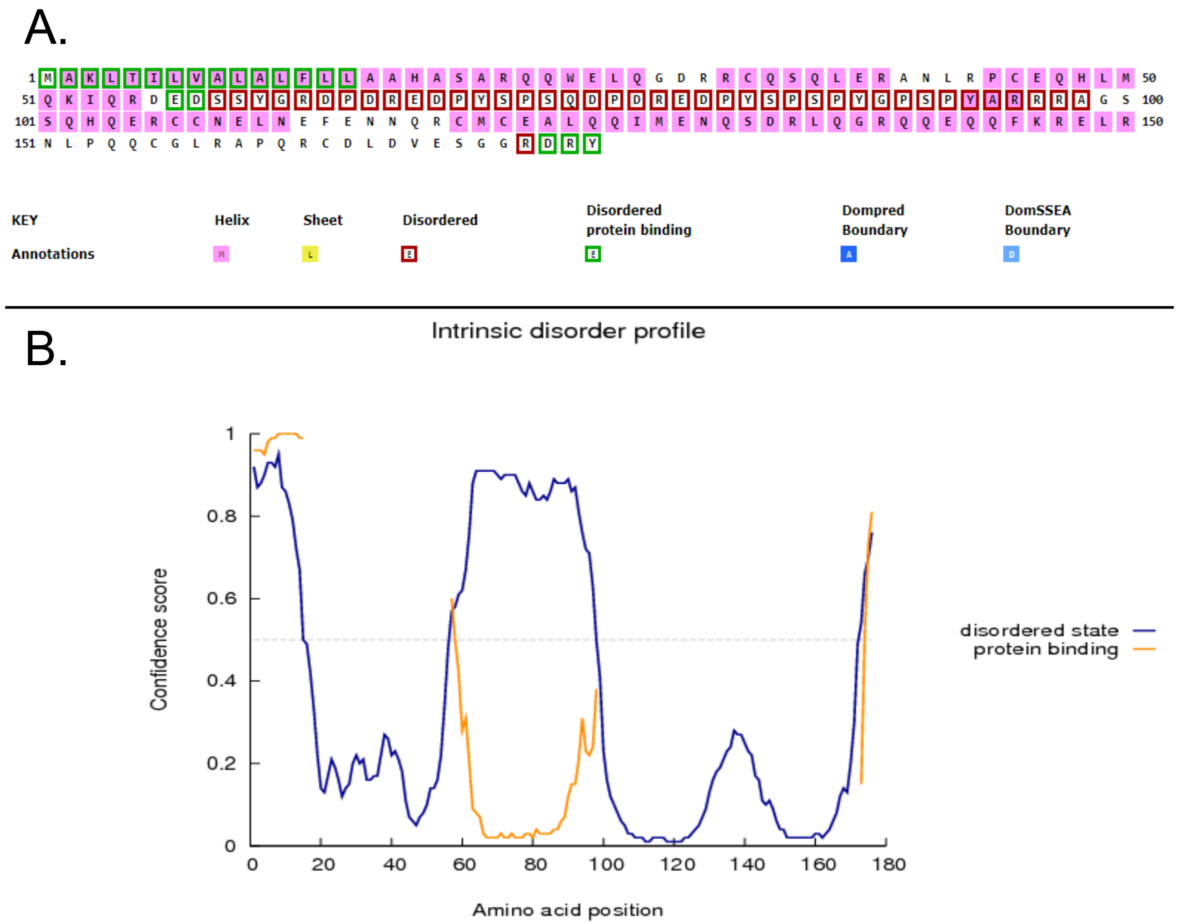

Figure S3M. PSIPRED sequence based disordered and disordered protein binding results for *A. palustris* Ara h 2. (A) shows the residues predicted to be disordered, involved in a helix, and to bind other proteins (e.g. IgE). (B) highlights the intrinsic disordered profile per amino acid position that align with key epitope regions of Ara h 2. See figure legends for coloring descriptions.

A.

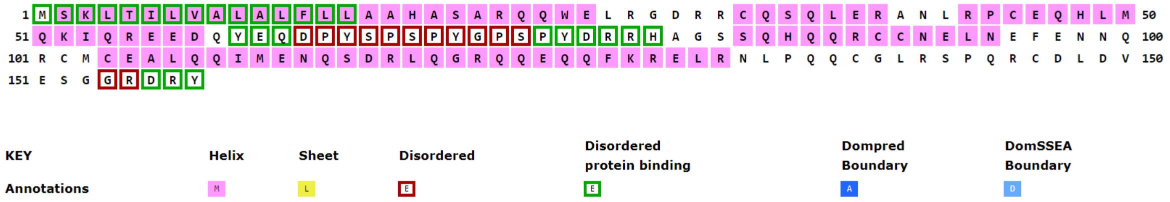

B.

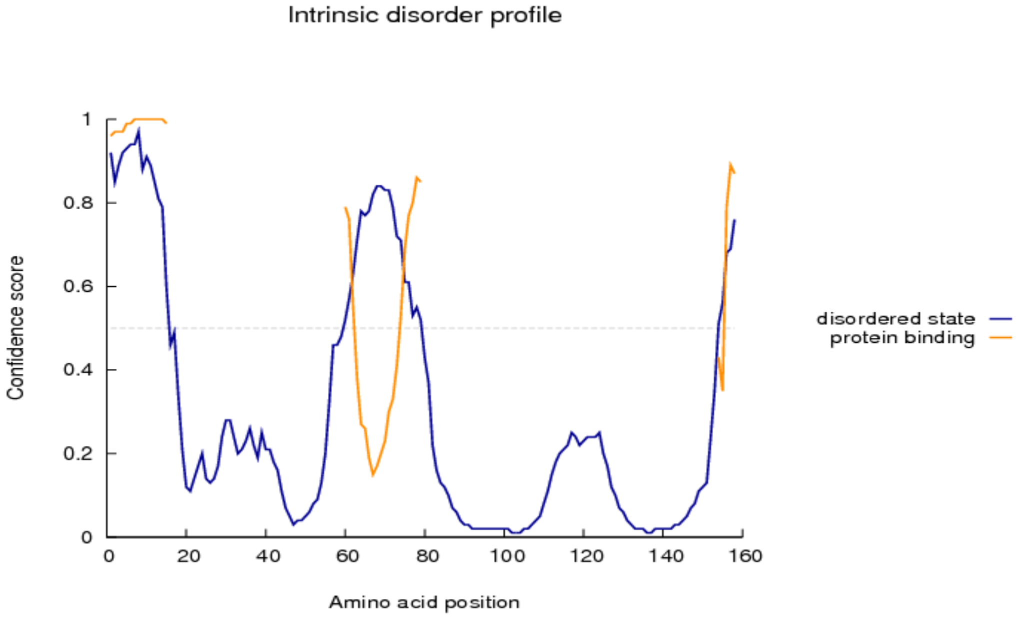

Figure S3N. PSIPRED sequence based disordered and disordered protein binding results for *A. pintoi* Ara h 2. (A) shows the residues predicted to be disordered, involved in a helix, and to bind other proteins (e.g. IgE). (B) highlights the intrinsic disordered profile per amino acid position that align with key epitope regions of Ara h 2. See figure legends for coloring descriptions.

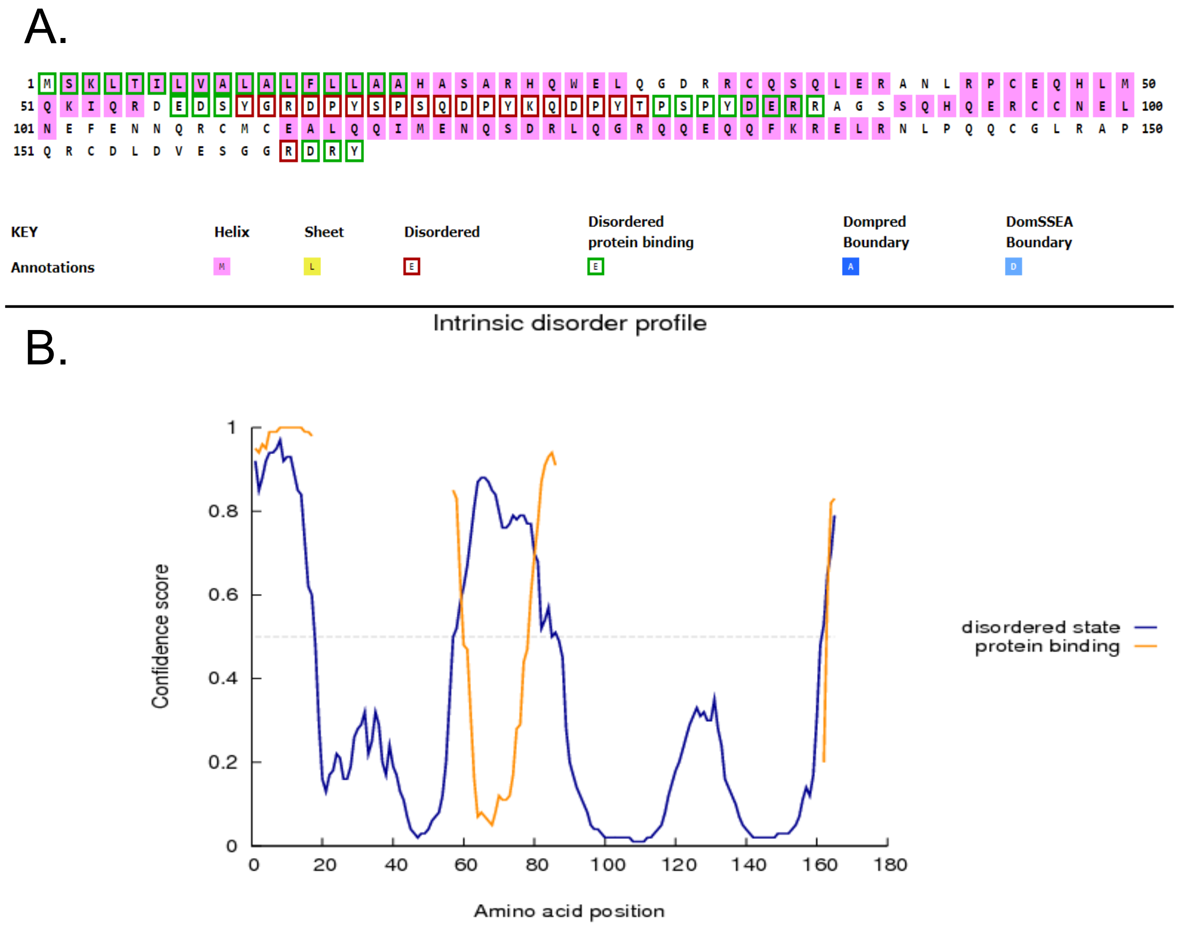

Figure S3O. PSIPRED sequence based disordered and disordered protein binding results for *A. glabrata* Ara h 2. (A) shows the residues predicted to be disordered, involved in a helix, and to bind other proteins (e.g. IgE). (B) highlights the intrinsic disordered profile per amino acid position that align with key epitope regions of Ara h 2. , See figure legends for coloring descriptions.

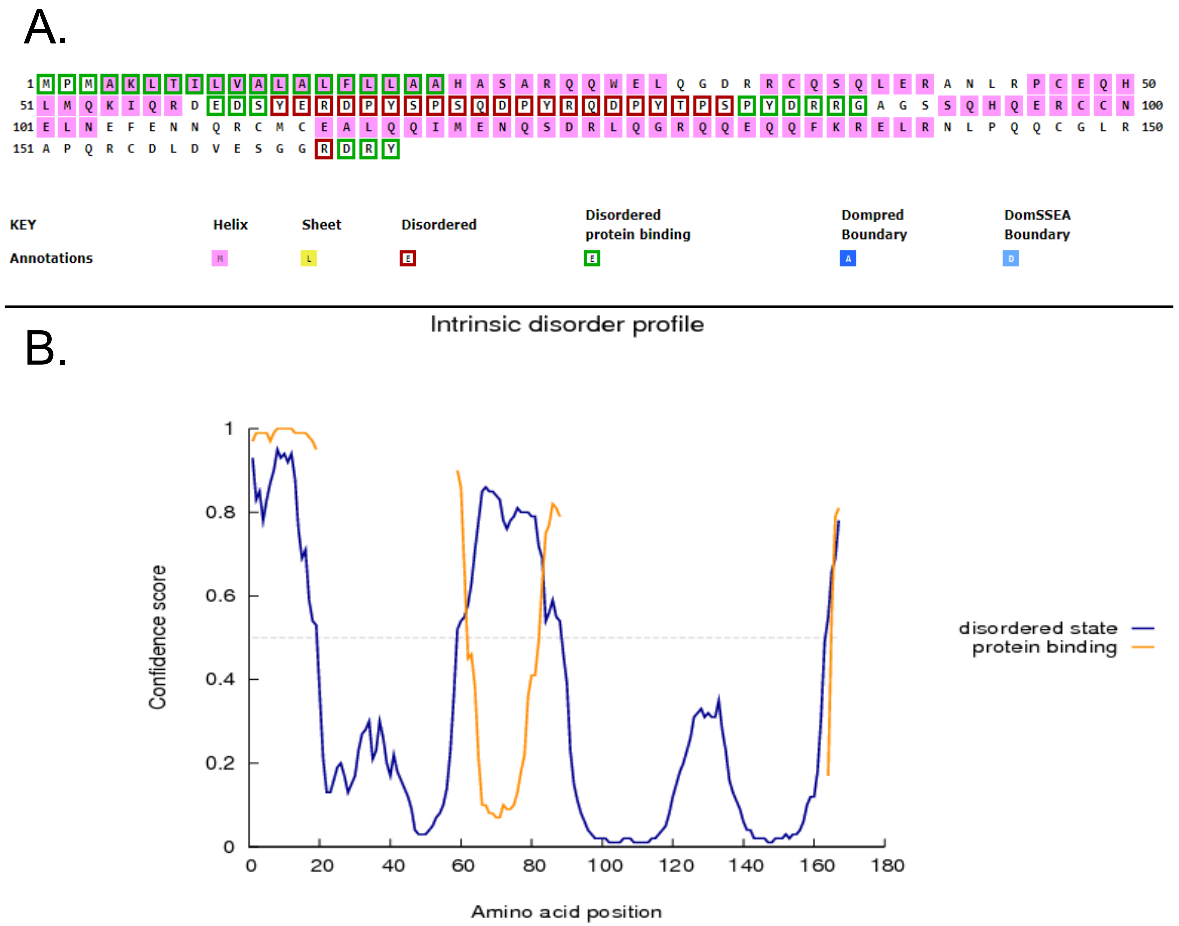

Figure S3P. PSIPRED sequence based disordered and disordered protein binding results for *A. hoehnei* Ara h 2. (A) shows the residues predicted to be disordered, involved in a helix, and to bind other proteins (e.g. IgE). (B) highlights the intrinsic disordered profile per amino acid position that align with key epitope regions of Ara h 2. , See figure legends for coloring descriptions.

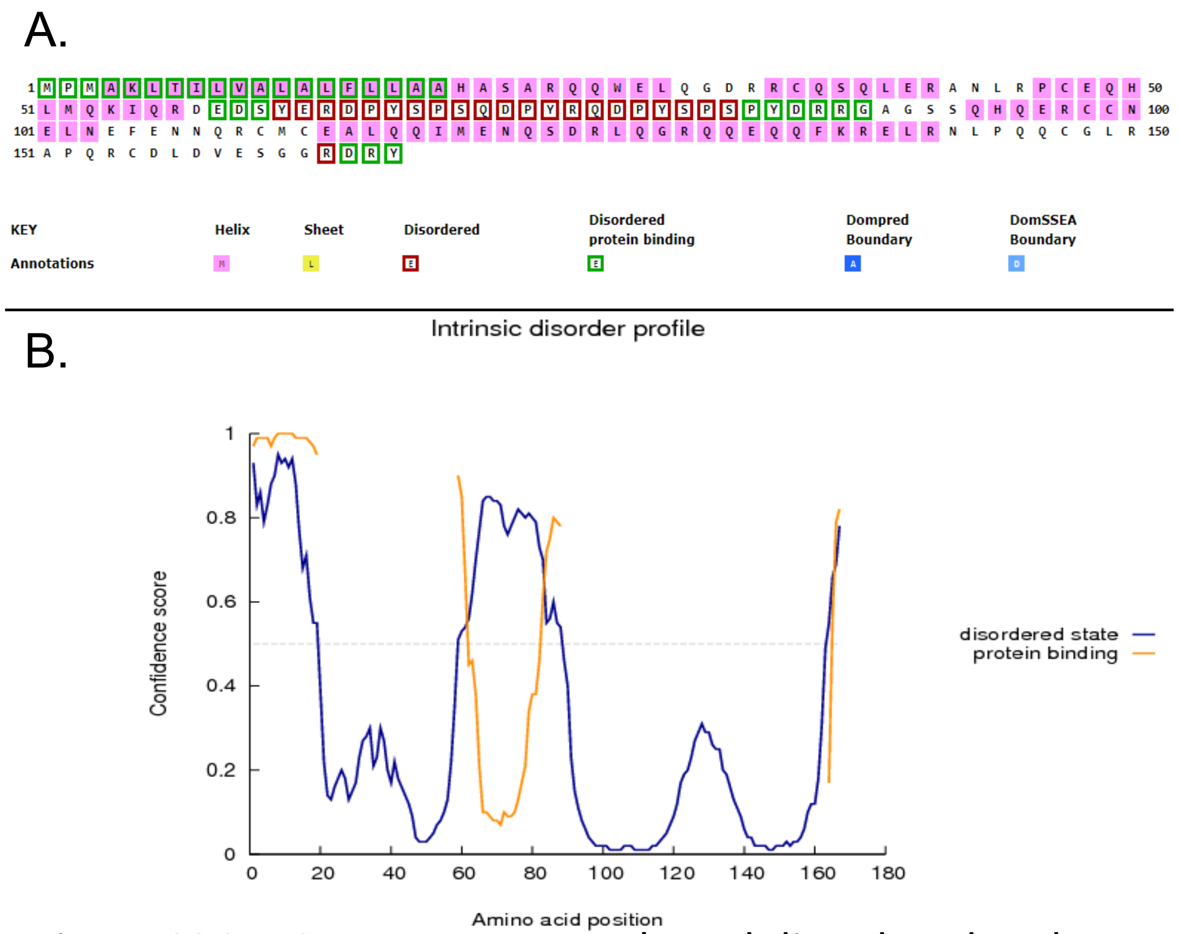

Figure S3Q. PSIPRED sequence based disordered and disordered protein binding results for *A. kretschmeri* Ara h 2. (A) shows the residues predicted to be disordered, involved in a helix, and to bind other proteins (e.g. IgE). (B) highlights the intrinsic disordered profile per amino acid position that align with key epitope regions of Ara h 2. See figure legends for coloring descriptions.

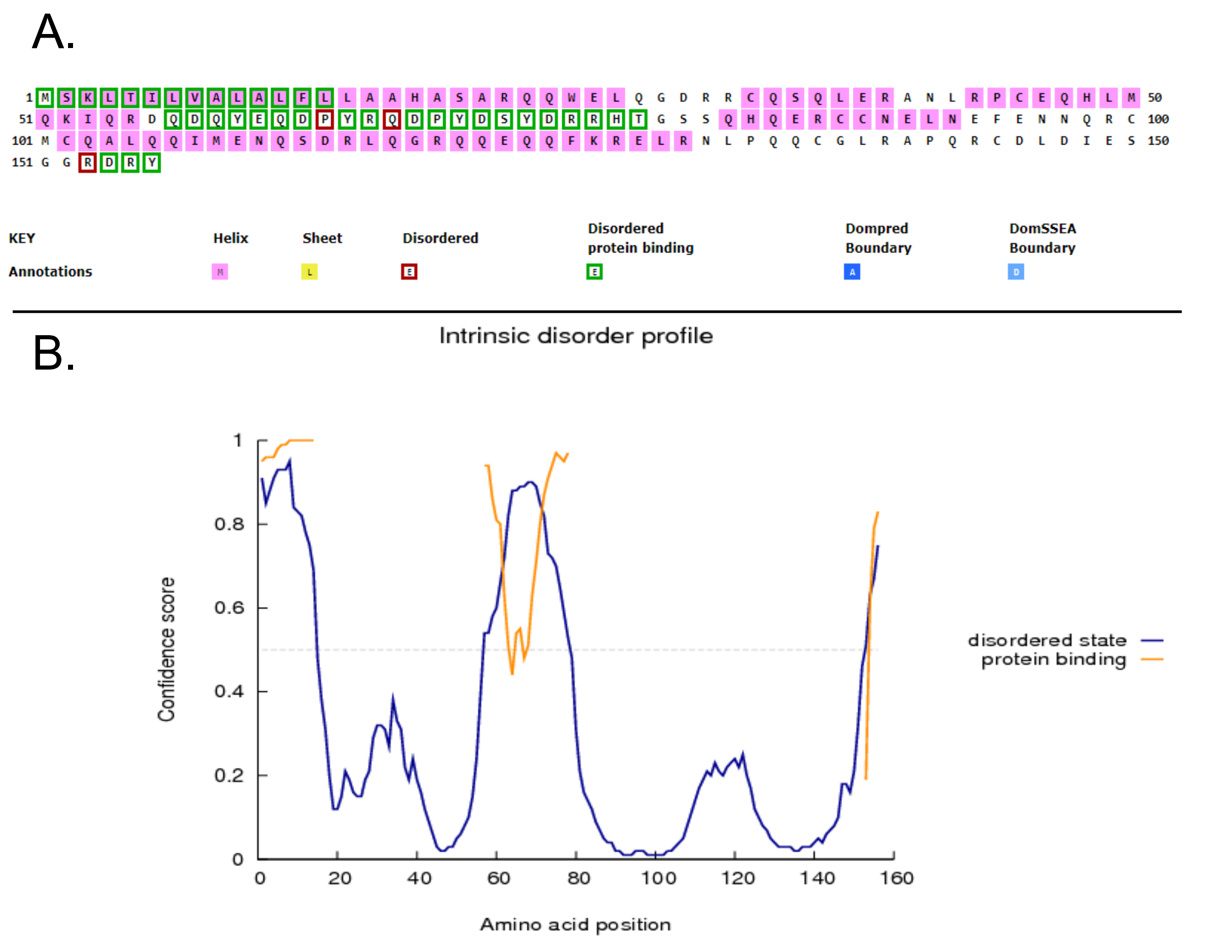

Figure S3R. PSIPRED sequence based disordered and disordered protein binding results for *A. macedo* Ara h 2. (A) shows the residues predicted to be disordered, involved in a helix, and to bind other proteins (e.g. IgE). (B) highlights the intrinsic disordered profile per amino acid position that align with key epitope regions of Ara h 2. See figure legends for coloring descriptions.

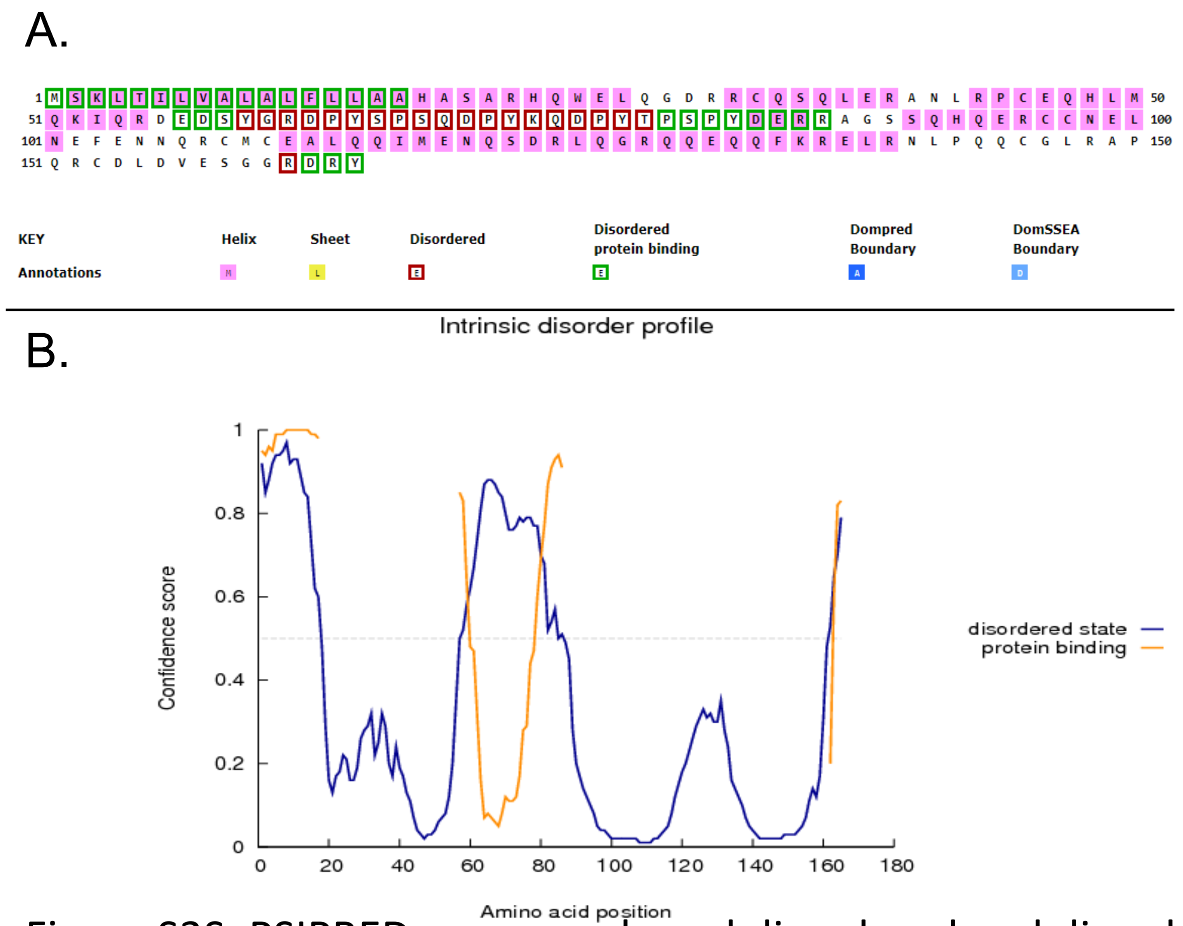

Figure S3S. PSIPRED sequence based disordered and disordered protein binding results for *A. batizocoi* Ara h 2. (A) shows the residues predicted to be disordered, involved in a helix, and to bind other proteins (e.g. IgE). (B) highlights the intrinsic disordered profile per amino acid position that align with key epitope regions of Ara h 2. See figure legends for coloring descriptions.

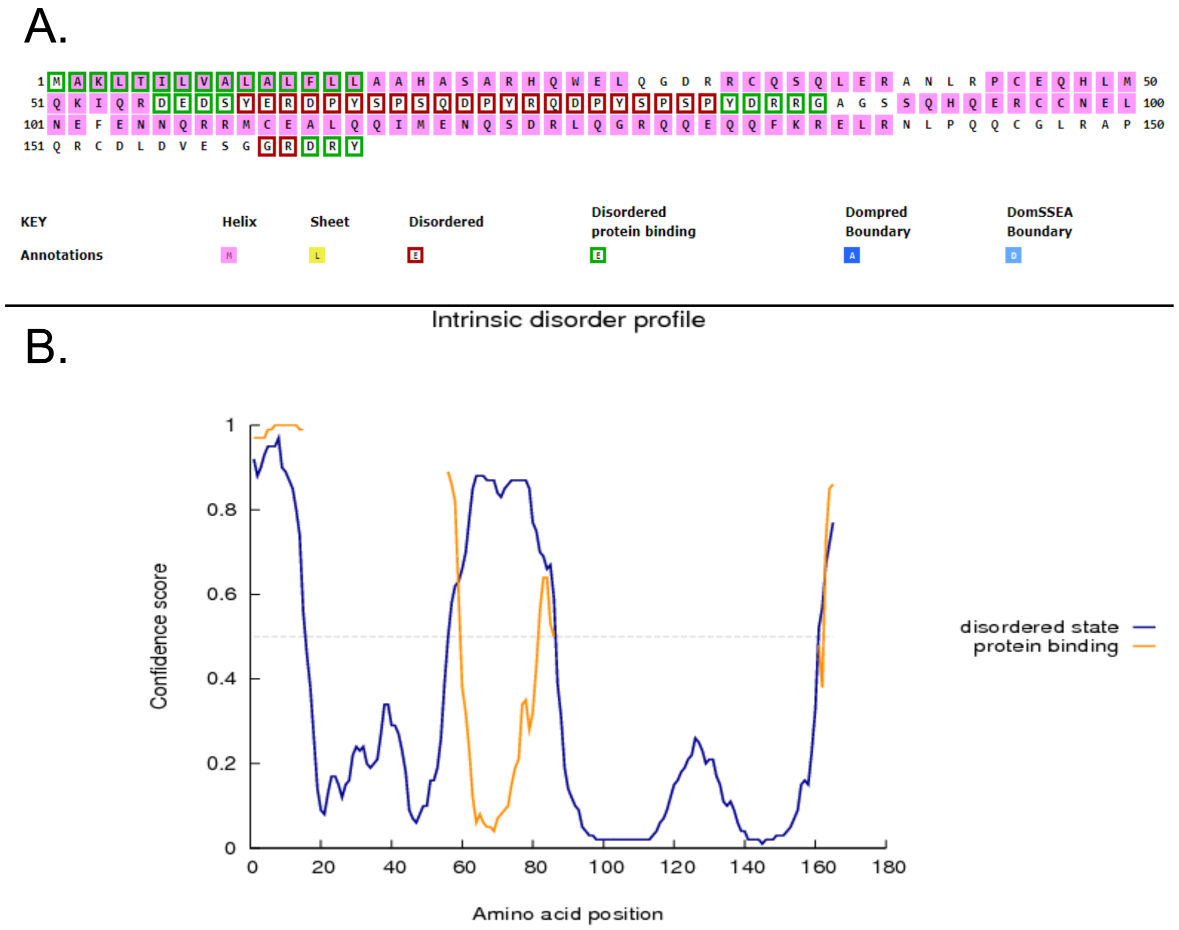

Figure S3T. PSIPRED sequence based disordered and disordered protein binding results for *A. lutescens* Ara h 2. (A) shows the residues predicted to be disordered, involved in a helix, and to bind other proteins (e.g. IgE). (B) highlights the intrinsic disordered profile per amino acid position that align with key epitope regions of Ara h 2. ,See figure legends for coloring descriptions.

A.

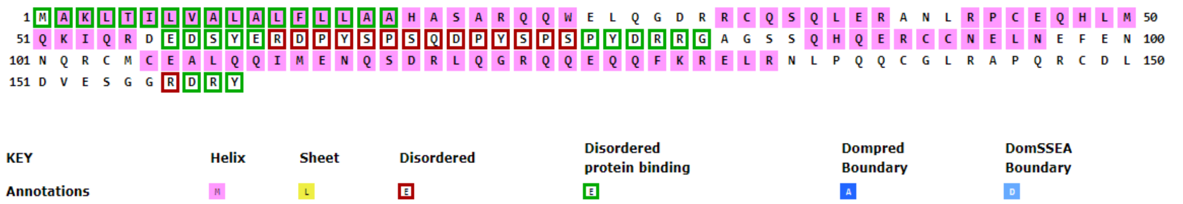

B.

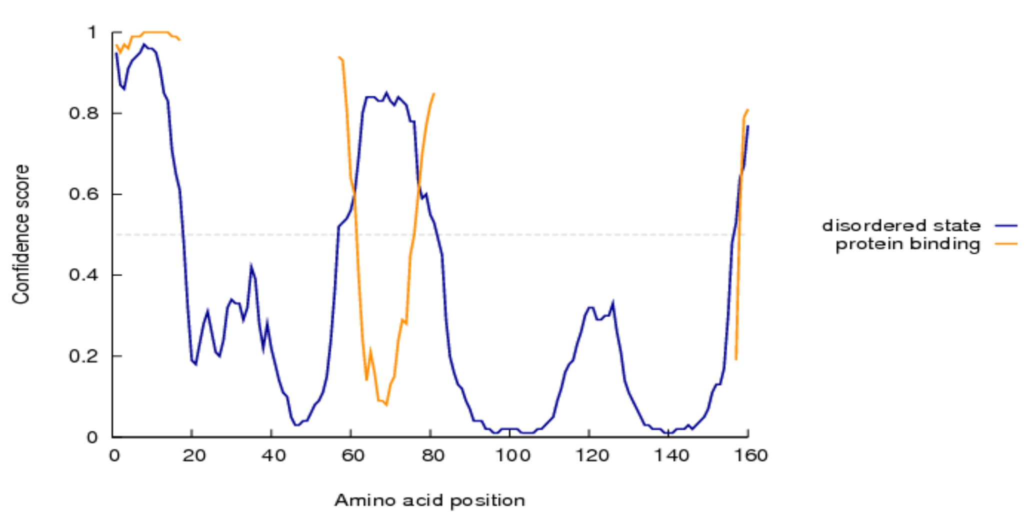

Figure S3U. PSIPRED sequence based disordered and disordered protein binding results for *A. villosulicarpa* Ara h 2. (A) shows the residues predicted to be disordered, involved in a helix, and to bind other proteins (e.g. IgE). (B) highlights the intrinsic disordered profile per amino acid position that align with key epitope regions of Ara h 2. See figure legends for coloring descriptions.

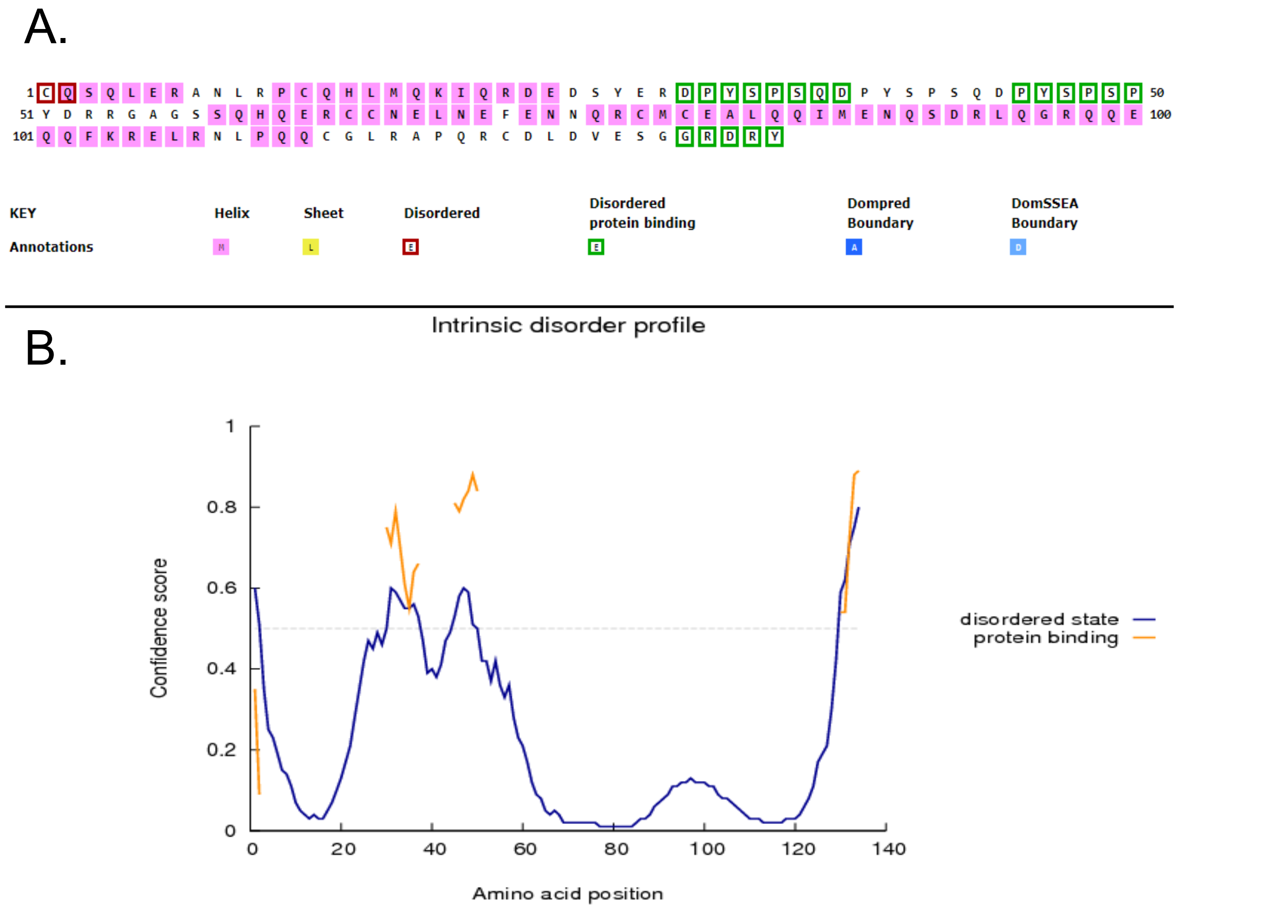

Figure S3V. PSIPRED sequence based disordered and disordered protein binding results for *A. helodes* Ara h 2. (A) shows the residues predicted to be disordered, involved in a helix, and to bind other proteins (e.g. IgE). (B) highlights the intrinsic disordered profile per amino acid position that align with key epitope regions of Ara h 2. See figure legends for coloring descriptions.

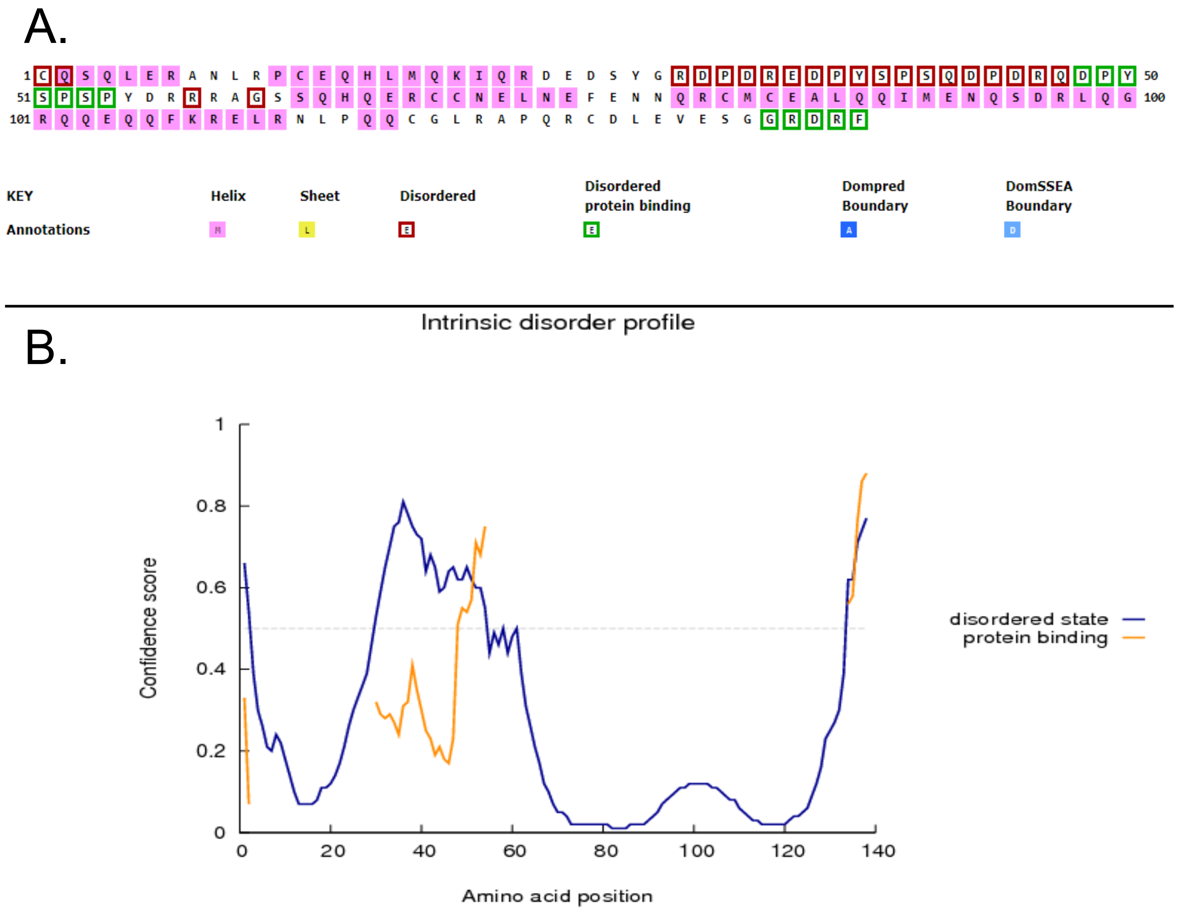

Figure S3W. PSIPRED sequence based disordered and disordered protein binding results for *A. trinitensis* Ara h 2. (A) shows the residues predicted to be disordered, involved in a helix, and to bind other proteins (e.g. IgE). (B) highlights the intrinsic disordered profile per amino acid position that align with key epitope regions of Ara h 2. See figure legends for coloring descriptions.
